# Structural basis for N-glycopeptide preference in an infant-derived *Bifidobacterium bifidum* PNGaseA

**DOI:** 10.1038/s41467-026-78972-1

**Published:** 2026-11-02

**Authors:** Cassie R. Bakshani, Simon G. Caulton, Victor Taleb, Taiwo O. Ojuri, Keith Coughlan, Alejandro Montesa, Alejandro Pérez-Latorre, Pedro Merino, Radoslaw P. Kozak, Sara Çakaj, Francesca Bottacini, Andrew L. Lovering, Douwe van Sinderen, Ramón Hurtado-Guerrero, Lucy I. Crouch

**Affiliations:** 1 Department of Microbes, Infection and Microbiomes, School of Infection, Inflammation and Immunology, College of Medicine and Health, Institute of Microbiology and Infection, University of Birmingham, Birmingham, UK; 2 School of Biosciences, University of Birmingham, Birmingham, UK; 3 Institute of Biocomputation and Physics of Complex Systems (BIFI), University of Zaragoza, Mariano Esquillor s/n, Campus Rio Ebro, Edificio I+D, Zaragoza, Spain; 4 APC Microbiome Ireland and School of Microbiology, University College Cork, Cork, Ireland; 5 Department of Biological Sciences, Munster Technological University, Cork, Ireland; 6 Department of Organic Chemistry. Faculty of Sciences, University of Zaragoza, Campus San Francisco, Zaragoza, Spain; 7 Ludger Ltd, Culham Campus, Abingdon, UK; 8 Copenhagen Center for Glycomics/Glycocalyx, Department of Cellular and Molecular Medicine, University of Copenhagen, Copenhagen, Denmark; 9 Fundación ARAID, Zaragoza, Spain

**Keywords:** Bacterial structural biology, X-ray crystallography

## Abstract

N-glycans are abundant nutrient sources for human gut bacteria, which deploy peptide: N-glycosidases to release them from glycoproteins. Breast milk is comprised of a high proportion of N-glycosylated bioactive proteins that can be accessed by infant colonic microbes. Here, we biochemically and structurally characterize a PNGaseA-family enzyme from infant-associated *Bifidobacterium bifidum* LMG13195. This enzyme acts on a broad range of N-glycan structures but shows a marked preference for N-glycans attached to glycopeptides rather than to denatured or native proteins, highlighting a specialized route for N-glycan acquisition in infant-associated *Bifidobacterium* species. Structures of the apo enzyme, an unliganded E236Q mutant, and a D107N–E236Q complex with a G2-peptide reveal a canonical two-β-sandwich PNGase core plus an additional ten-strand β-sheet cradle and are the first structures of this superfamily. Comparison of these states, combined with molecular dynamics simulations, indicates that glycopeptide binding is coupled to β-sheet cradle-loop movements that close against β-sandwich 1 over the peptide C terminus. The complex defines recognition of the two core GlcNAc residues and the central mannose, reveals that peptide contacts are concentrated around the +2 Thr residue of the NXT sequon, and positions the scissile amide at the active site. These findings, together with mutational and computational analyses, provide a structural basis for N-glycopeptide preference by linking glycopeptide binding to cradle closure and catalysis, thereby expanding the host-glycan nutrient landscape accessible to infant-gut *Bifidobacterium* species.

## Abstract (web summary)

Here, the authors show that a *Bifidobacterium* PNGaseA uses a β-sheet cradle to recognize N-glycopeptides, linking sequon recognition, cradle closure and catalysis during infant-gut glycan acquisition.

## Introduction

N-glycans are posttranslational modifications on proteins, particularly common on proteins secreted extracellularly, such as antibodies^1^. In the context of the human colonic microbiota, the prevalence of this N-glycosylation means that they are ubiquitously available for these microbes to use as a nutrient source. N-glycans are both host-produced and diet-derived (from both animal and plant sources). Furthermore, fungal yeasts produce large mannan N-glycan structures as a part of their cell wall, which are available to colonic microbes derived from yeast-fermented foods and mycobiota residing in the colon^2^.

There are now several studies characterising the enzymology of N-glycan degradation by human-associated microbes: *Bacteroides thetaiotomicron* and mammalian complex N-glycans^3^, *Bacteroides thetaiotomicron* and yeast mannan^2^, *Streptococcus pneumoniae* and both high-mannose and complex N-glycans^4,5^, *Phocaeicola massiliensis* and plant/insect N-glycans^6^, and *Bifidobacterium* species and high-mannose N-glycans^7^. There are three families of enzymes that remove the N-glycan from the peptide component and one of these is the Peptide:N-glycosidases (PNGases), which hydrolyse between the protein and the core GlcNAc^1^. The two other families are glycoside hydrolase (GH) families 18 and 85, whose members hydrolyse between the two core GlcNAc sugars to separate the glycan from the protein^1^.

Among PNGases and related glycoamidases, catalysis is not uniform, and several different mechanisms have been proposed. The cytoplasmic PNGases Png1/NGLY1, which act on misfolded glycoproteins during ER-associated degradation, belong to the transglutaminase-like superfamily and use a cysteine protease-type mechanism, with a Cys-His-Asp catalytic triad in which cysteine acts as the nucleophile^8^. By contrast, secreted PNGaseF uses an acidic-residue-based catalytic centre. Structural understanding of this class has been dominated by PNGaseF, whose crystal structures revealed a catalytic core formed by two β-sandwich domains and, in a complex with chitobiose, identified Asp60, Glu118 and Glu206 as key residues for catalysis and glycan recognition^9,10^. For comparison, the related glycosylasparaginases use a mechanistically distinct route in which a processed N-terminal threonine acts as both the base and the nucleophile, a hallmark of the N-terminal nucleophile (Ntn) hydrolase family^11^. This mechanism was previously argued to differ from that of PNGaseF^10^. However, because these PNGaseF studies did not capture a full N-glycopeptide substrate, it remained unclear how secreted PNGases recognize the peptide portion of the substrate. Critical aspects that remain unresolved are the position the glycosylated Asn in the catalytic site and how PNGases engage features of the canonical N-glycosylation sequon, N-X-S/T (where X is any residue except Pro). Notably, this sequon is also recognized by oligosaccharyltransferases^12^ and by soluble bacterial N-glycosyltransferases such as *Aggregatibacter aphrophilus* NGT^13^.

The infant colonic microbiota is dominated by species of *Bifidobacterium*, especially in breast-fed infants, at least partly due to their ability to directly or indirectly metabolise human milk oligosaccharides as their main carbohydrate and energy source^14^. However, N-glycans are also present in human milk and some species of *Bifidobacterium* have characterised GH18 and GH85 enzymes that are active against N-glycoproteins^15^. The presence of these enzymes in infant-associated species of *Bifidobacterium* indicates that N-glycans are also a source of nutrition. One notable observation showed growth of *B. longum* subsp. *infantis* ATCC 15697 on a mixture of complex N-glycans released from whey, including both sialylated and non-sialylated structures, of which the majority were degraded during growth^16^. In terms of high-mannose N-glycan breakdown, the full enzymatic machinery from a *Bifidobacterium longum* strain has been biochemically characterised^17^. Finally, N-glycan fragments have been observed in the faeces of breastfed infants, and the dominant bacteria were species of *Bifidobacterium*^18^.

The ability of *Bifidobacterium* species to breakdown N-glycans as a nutrient source is thus evident from available literature, yet the initiating steps and their structural basis remain poorly defined. To address this, we searched genomes of infant-associated *Bifidobacterium* species and identified a single putative PNGaseA-family enzyme in two *B. bifidum* strains, including *B. bifidum* LMG13195 (PNGaseA^Bif^). Biochemical analyses showed that PNGaseA^Bif^ accommodates a broad range of N-glycan structures but preferentially processes N-glycans on short glycopeptides rather than on denatured or native glycoproteins. To understand the basis of this preference, we combined biochemical characterization with structures of the apo enzyme and substrate-relevant mutant states, including a complex with a G2-peptide. Structurally, PNGaseA^Bif^ retains the canonical PNGase catalytic core seen in PNGaseF, comprising two β-sandwich domains, here designated β-sandwich 1 and β-sandwich 2, but also contains an additional ten-strand β-sheet cradle. These analyses reveal that glycopeptide binding is coupled to closure of the β-sheet cradle over the peptide region of the substrate through movements in β-sheet cradle loops that approach β-sandwich 1. Molecular dynamics simulations further support that this region is intrinsically mobile in the apo state, consistent with the open-to-closed transition observed upon substrate binding. Together, these findings establish a structural basis for the preference of PNGaseA^Bif^ for N-glycopeptide substrates, with β-sheet cradle closure creating a more shielded active-site environment than the comparatively solvent-exposed catalytic cleft of PNGaseF.

## Results

### A rare PNGaseA-family enzyme in infant-associated *B. bifidum*

To investigate N-glycan utilisation by species of human-associated *Bifidobacterium* species, InterPro, UniProt and IMG databases were screened for putative PNGase-encoding sequences, and this resulted in the identification of a predicted PNGase gene present in the genome of *Bifidobacterium bifidum* LMG13195 (the enzyme is denoted here as PNGaseA^Bif^, while its corresponding gene is referred to here as *pngaseA*^*bif*^)^19–21^. Intriguingly, PNGaseA^Bif^ belongs to the PNGaseA superfamily, which is not currently associated with nutrient acquisition in bacterial species^19^.

In the InterPro database, the current putative PNGaseA superfamily members are present in 1404, 710 and 94, eukaryotic, bacterial and archaeal species, respectively^19^. Within the eukaryotes, 1009 species are fungi (mainly Ascomycota and Basidiomycota), 311 species are flowering plants, while the remainder currently represent unclassified species. Within bacteria, 571 of the species with a putative PNGaseA-encoding sequence are Actinobacteria, where 444 of these are species of *Streptomyces*. The rest of the bacterial species represent a wide variety of microorganisms that are members of the Pseudomonadati kingdom. Of these bacterial sequences, there is only one additional putative PNGaseA enzyme (other than that specifying PNGaseA^Bif^) associated with a member of the *Bifidobacterium* genus, *Bifidobacterium leontopitheci* (A0A6I1GFR2) isolated from Golden-headed Lion Tamarin and Goeldi’s monkey^22^. However, this putative PNGaseA member elicits just 25 % protein sequence identity to PNGaseA^Bif^.

To investigate the genetic context of *pngaseA*^*bif*^ in the genome of *Bifidobacterium bifidum* LMG13195 and to assess why this gene is apparently absent from other members of this species, we analysed corresponding chromosomal regions in several *B. bifidum* strains from the NCBI genome database (Supplementary Table 1). Eighteen complete genomes were available at the time of this comparative analysis and revealed that *pngaseA*^*bif*^ is part of a highly variable region flanked by two conserved regions (Supplementary Figs. 1, 2). In *B. bifidum* LMG13195, this variable region contains the PNGaseA^Bif^ gene and various transposase-encoding genes, suggesting that *pngaseA*^*bif*^ was acquired by horizontal gene transfer. Overall, eight different types of inserts were detected in this variable region for these eighteen genomes (Supplementary Fig. 2). Through this analysis, a second *B. bifidum* strain (Bg41221_3D10), isolated from a human infant in Bangladesh in a recent project, has a very similar gene pattern in this variable region, including a *pngA* of high identity to that found in LMG13195^23^ (Supplementary Fig. 2).

**Fig. 1:**
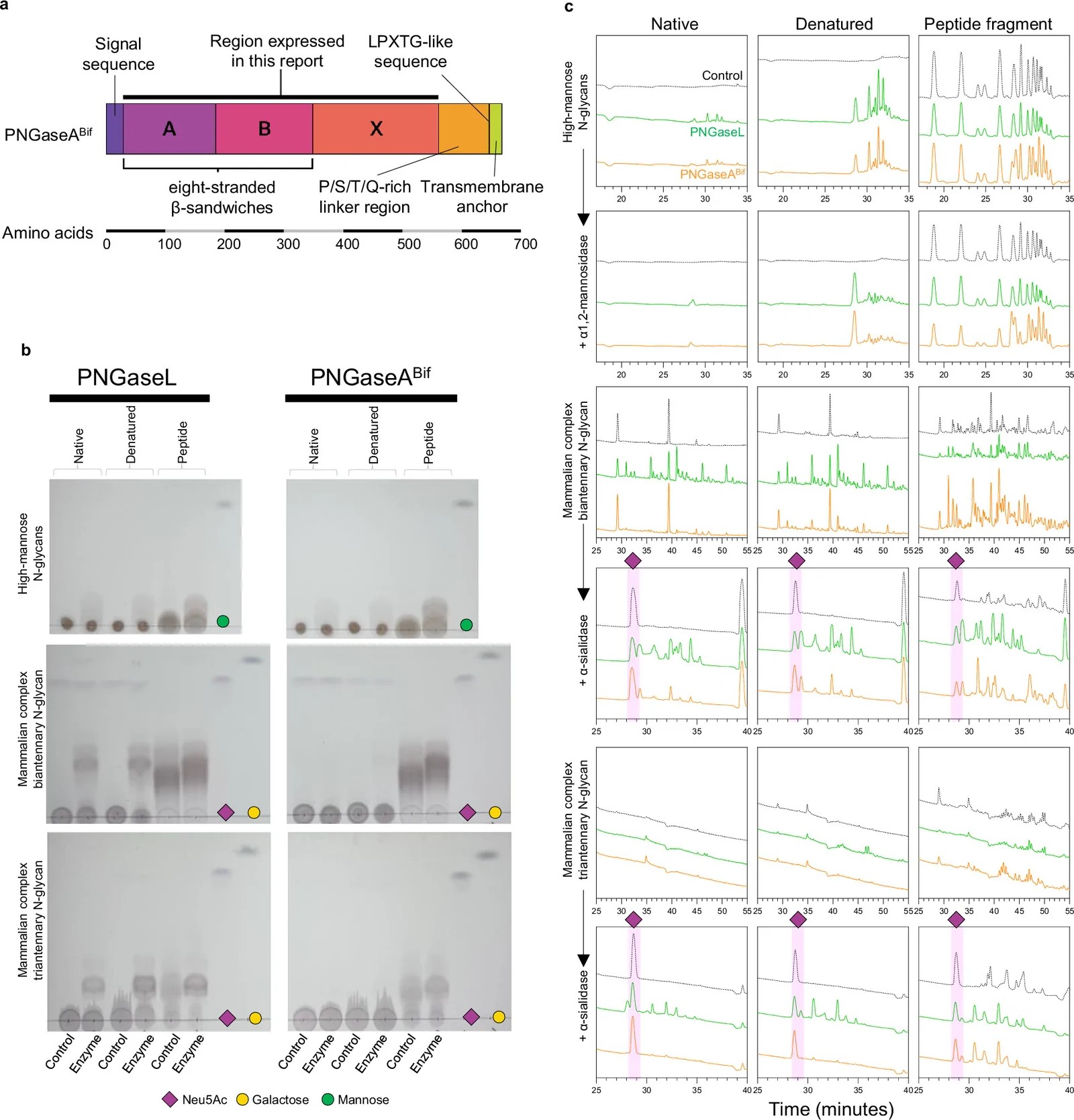
PNGaseA^Bif^ domain structure and activity against different glycoprotein and glycopeptide substrates. **a** The two eight-stranded β-sandwiches predicted to comprise the catalytic module are labelled A and B. The section after this, labelled X, was not assigned any annotation by databases. Towards the C-terminus of the protein a region of low complexity was highlighted by searches and is comprised of predominantly proline, threonine, serine and glutamine, which suggests this area is a linker region. The C-terminus is predicted to be a transmembrane region, suggesting that the enzyme is anchored to the cell surface of *B. bifidum*. The black line represents the section cloned in this study. **b** Recombinantly-expressed PNGaseL and PNGaseA^Bif^ were tested against glycoprotein substrates with different types of N-glycans and with the protein in the native form, pre-treated with boiling (denatured) and with the protein mostly digested away (peptide) and the results assessed by TLC. **c** The specificity of PNGaseA^Bif^ was further analysed using HPAEC-PAD. Additional assays were also included to probe specificity towards N-glycan structures with the non-reducing end capping sugars removed. To do this, the high-mannose N-glycan and complex N-glycan substrates were pretreated with an α1,2-mannosidase (BT3990) and a broad-acting α-sialidase (BT0455), respectively. Full data sets are presented in Supplementary Figs. 3–5). Source data are provided in the Source Data file. Assays were carried out for three biological replicates and the samples were run on the HPAEC-PAD once.

Initial analysis of the PNGaseA^Bif^ amino acid sequence revealed several characteristics (Fig. 1). Sequence alignments of PNGaseA^Bif^ to PNGaseF superfamily representatives highlighted the likely eight-stranded β-sandwiches in the first N-terminal half of the PNGaseA^Bif^ sequence. There is a large region after this which had no homology to the PNGaseF representatives, labelled ‘X’ and then a proline/serine/glycine/glutamine-rich region that is likely a linker. There is then a LPXTG-like motif, which may be a site for sortase-dependent anchoring to the cell wall, and finally a predicted transmembrane region at the C-terminus^24^. The signal sequence is predicted to be a Sec signal peptide (SPI), indicating that PNGaseA^Bif^ is produced by *Bifidobacterium bifidum* LMG13195 as an extracellular, membrane-anchored enzyme^25^.

### PNGaseA^Bif^ preferentially cleaves N-glycans from glycopeptides

Previously characterised members of the PNGaseA superfamily exhibit a preference for N-glycans that are covalently linked to short peptide rather than native proteins^26^. Therefore, assays were designed to test specificity towards both the N-glycan and protein components of these substrates. This was achieved, using three glycoprotein substrates with different types of N-glycosylation and preparing the substrates in three different ways to test the impact of the protein component of the substrate. These different preparations were – no pre-treatment (native), boiling (denatured) and proteolytic digestion with the highly proteolytic coral pathogen, *Vibrio coralliilyticus*^27^, to remove most of the protein. In addition to this, the high-mannose N-glycan and complex N-glycan substrates were also pretreated with an α1,2-mannosidase (BT3990) and a broad-acting α-sialidase (BT0455), respectively, to trim down these substrates from the non-reducing end and fully explore potential activity (Fig. 1 and Supplementary Figs. 3-5)^2,28^. PNGaseA^Bif^ was determined to possess activity primarily towards N-glycans attached to just a peptide, some activity against the denatured substrates, and no activity against the native version. PNGaseL was included as the positive control and activity was observed under all conditions^29^.

To analyse the released glycans from these assays in more detail, high-performance anion exchange chromatography with pulsed amperometric detection (HPAEC-PAD; Fig. 1) was used. PNGaseA^Bif^ released high mannose N-glycans of a variety of sizes (Man5-8). For the glycopeptide substrate, the high background means that the peaks corresponding to the different glycans are not clearly identifiable, however, the peak corresponding to Man5 has been highlighted. Furthermore, the chromatograms for the samples where PNGaseA^Bif^ was included are different to the control, whereas the PNGaseL chromatograms look akin to the control. The data suggest that the addition of an α1,2-mannosidase, under these assay conditions, does not affect the efficiency of the control or PNGaseA^Bif^. The HPAEC-PAD data from assays against glycoproteins with mammalian complex N-glycans also support the conclusions drawn from the TLCs; the activity observed for PNGaseA^Bif^ when the N-glycan is attached to a peptide fragment is considerably higher. The addition of α-sialidase, under these assay conditions, does not appear to affect the efficiency of the control or PNGaseA^Bif^.

**Fig. 2:**
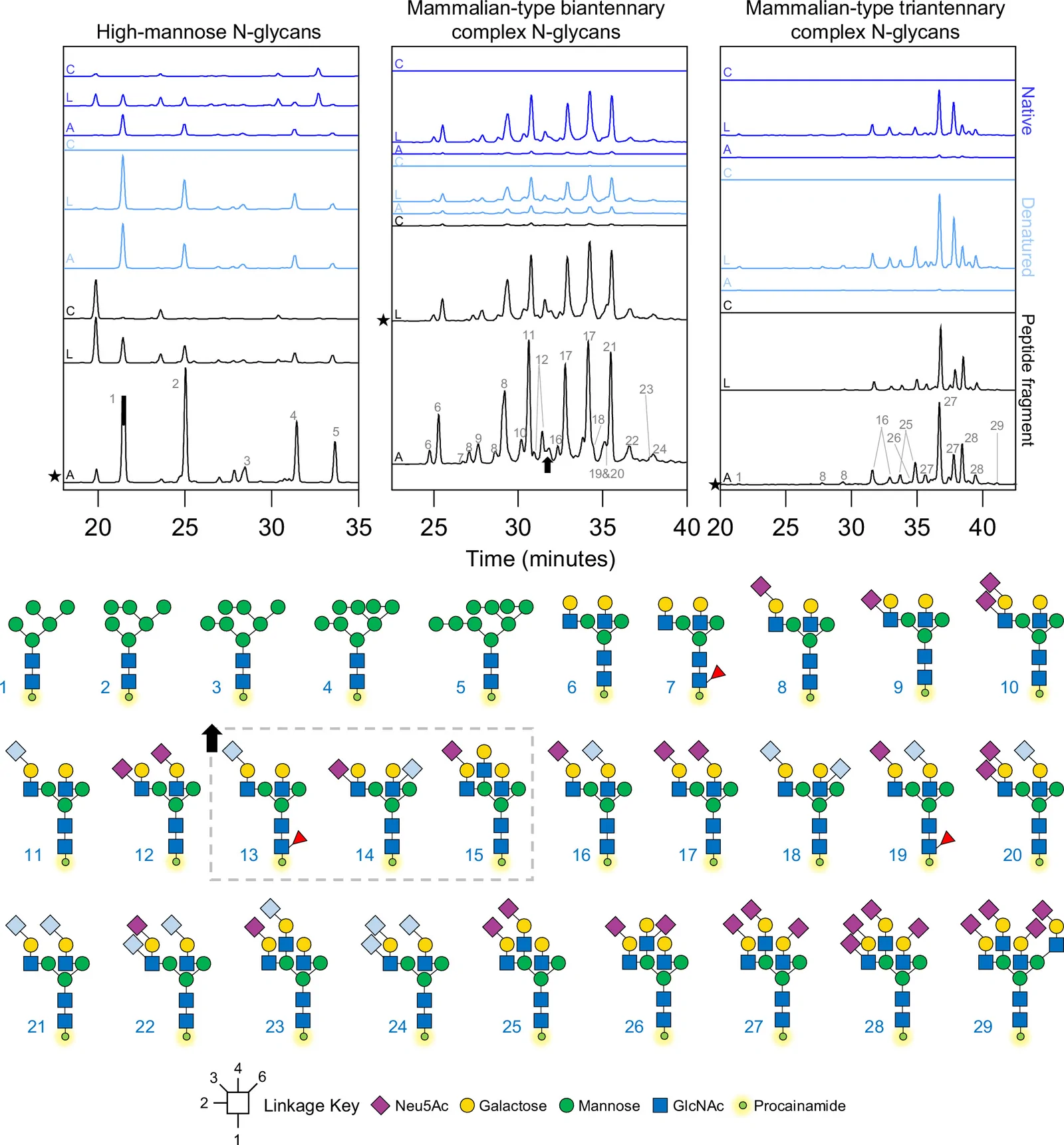
Assessing the specificity of PNGaseA^Bif^. Glycans released by PNGase were labelled with procainamide and analysed by LC-FLD-ESI-MS. C, L, and A stand for Control, PNGaseL, and PNGaseA^Bif^, respectively. The numbered peaks correspond to the structures of the glycans provided below the chromatograms. The structures of the glycans were determined using MS/MS data (Supplementary Figs. 6, 7). The samples with chromatograms labelled with stars had to be diluted by half relative to the other samples to obtain fluorescence signals that were not over saturated. The fluorescence signals reflect the concentration of N-glycans in the original samples, however, the labelling and clean up processes may lead to a loss of some sample. Therefore, this should be taken into consideration when interpretating the results. Despite this, these data strongly support the conclusions about substrate preference of PNGaseA^Bif^. Source data are provided in the Source Data file and the LC-FLD-ESI-MS data collection was carried out once.

To investigate the specificity of PNGaseA^Bif^ in more detail, products from the assays were labelled with the fluorophore procainamide and analysed by liquid chromatography-fluorescence detection-electrospray-mass spectrometry (LC-FLD-ESI-MS; Fig. 2). The structures of the N-glycans were determined using MS/MS (Supplementary Figs. 6 and 7). The data show that PNGaseA^Bif^ releases the same N-glycan structures as the PNGaseL positive control, which include core fucosylation, tri- and tetraantennary branching, and sialylated antennae. Quantitatively, however, the comparison between the two enzymes depended on N-glycan type, with PNGaseA^Bif^ releasing less N-glycan than PNGaseL from the complex substrates but more than PNGaseL from the high-mannose substrate. Time course assays with PNGaseA^Bif^ and PNGaseL also strongly support the substrate specificity conclusions (Supplementary Figs. 8-10).

To assess the influence of α-fucosylation of the core GlcNAc on activity, we also used the plant-derived and insect-derived glycoproteins (horseradish peroxidase and phospholipase A_2_ from honeybee venom, respectively) as substrates (Supplementary Fig. 11). It was only possible to carry out these experiments under native and denatured conditions due to the nature of the proteins (not enough to test bacterial growth and subsequent purification). PNGaseA^Bif^ was able to release glycans from the horseradish peroxidase, suggesting that core α1,3-fucose can be accommodated in the active site. However, it was not able to release any glycans from the phospholipase A_2_. As both glycoproteins carry core α1,3-fucose, this difference is unlikely to be explained by the fucose modification alone and may instead reflect an influence of the underlying protein or peptide environment on N-glycan release. A comparable dependence has been reported for FUT8, whose core α1,6-fucosylation of N-glycans is regulated not only by the glycan structure but also by the protein environment^30^. Activity against plant and insect type N-glycans has been observed previously for both PNGaseA and PNGaseF superfamily members^1^.

### PNGaseA^Bif^ possesses a unique β-sheet cradle structure

**Fig. 3:**
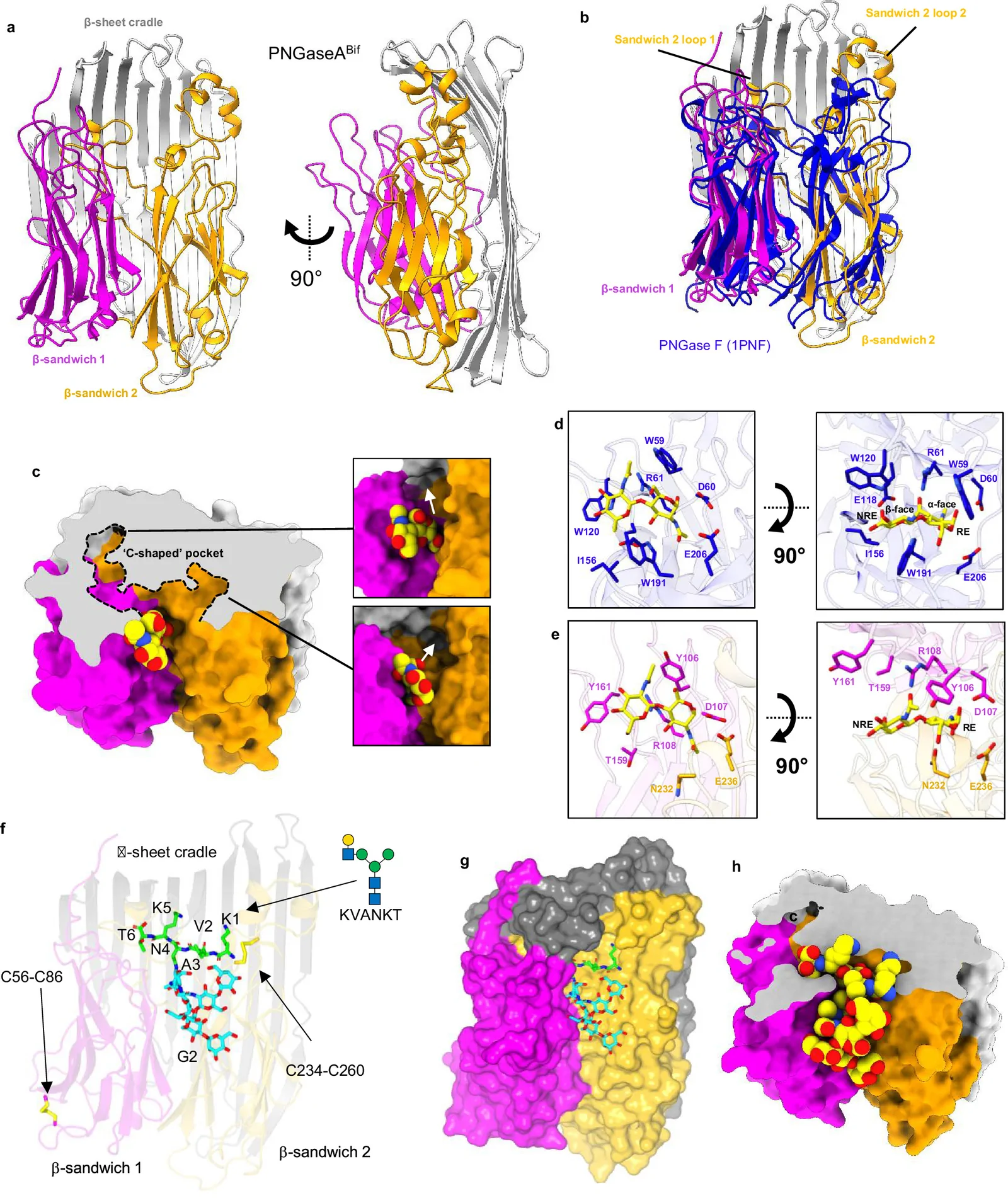
The PNGaseA^Bif^ structure has a β-sheet cradle that drives substrate specificity. **a** A cartoon representation of the PNGaseA^Bif^ structure shown from two different angles with the β-sandwiches coloured in magenta and gold and the β-sheet cradle in grey. **b** An overlay of the eight-stranded β-sandwich catalytic domains from PNGaseA^Bif^ and PNGaseF (1PNF, blue). **c** A cross-section of the active site to show the C-shape pocket that likely accommodates short peptides (dotted line) and two closeup views of the active site, looking into the two pockets where the short peptides will likely be accommodated. **d** The active site of PNGaseF 1PNF with chitobiose (yellow) shown from two different angles. **e** The active site of PNGaseA^Bif^ with the chitobiose (yellow) from 1PNF overlaid. **f** Overall cartoon representation of the PNGaseA^Bif^ D107N–E236Q–G2-peptide complex, with β-sandwich 1 shown in magenta, β-sandwich 2 in gold, and the β-sheet cradle in grey. The glycan is shown as cyan sticks and the peptide moiety (K1–T6) as green sticks; disulfide bonds C56–C86 and C234–C260 are shown in yellow. **g** Surface representation showing the ligand bound in a central cleft formed between the catalytic core and the β-sheet cradle. **h** A cross-section of the active site of the double mutant to show the substrate sitting in the C-shape pocket.

To investigate the structural basis for the unusual specificity displayed by PNGaseA^Bif^ we determined the apo wild-type crystal structure to 1.9 Å (Fig. 3 and Supplementary Table 2, Supplementary Fig. 12). The PNGaseA^Bif^ catalytic module comprises two eight-stranded β-sandwich domains and this overlays well with the solved structures from the PNGaseF superfamily. To demonstrate this, superimposition of the crystal structure of archetypal PNGaseF from *Elizabethkingia meningoseptica* (1PNF) and PNGaseA^Bif^ shows an RMSD of 4.8 over 248 residues. Strikingly, however, in the PNGaseA^Bif^ structure there is an additional large ten-strand β-sheet closely associated with this core catalytic module which cradles the entirety of one side of the catalytic module. This is absent in all other solved PNGase structures^6,10,31^. A magnesium ion was observed in the active site of the apo structure, but its position within the substrate-binding region, and its absence in the glycopeptide-bound complex described below, indicate that it is a crystallization artifact. (Supplementary Information and Supplementary Fig. 13).

### The β-sheet cradle creates a C-shaped pocket and drives specificity for the substrate niche

Analysis of the area around the active site provides insights into why PNGaseA^Bif^ prefers N-glycans on a peptide. Either side of where the reducing end of the core GlcNAc would be positioned are two long pockets that together form a “C” shape (Fig. 3). In comparison to this, the PNGaseF structure is very open around the catalytic site, likely allowing it to accommodate N-glycans attached to larger protein components (Supplementary Fig. 12). An electrostatic overlay was applied to the PNGaseA^Bif^ apo structure to visualise the charge of the surface (Supplementary Fig. 12). Interestingly, one of the pockets is highly negatively charged and the entrance to the other is highly positively charged. We predicted that a structure with these features would support the accommodation of the charged amino- and carboxy- termini of a peptide and support the overall proposal that the function of the C-shaped pocket is to accommodate a peptide. We were able to confirm and explore the details of this through subsequent structures with ligand and simulations.

### Generation of the first PNGase-glycopeptide structure

**Fig. 4:**
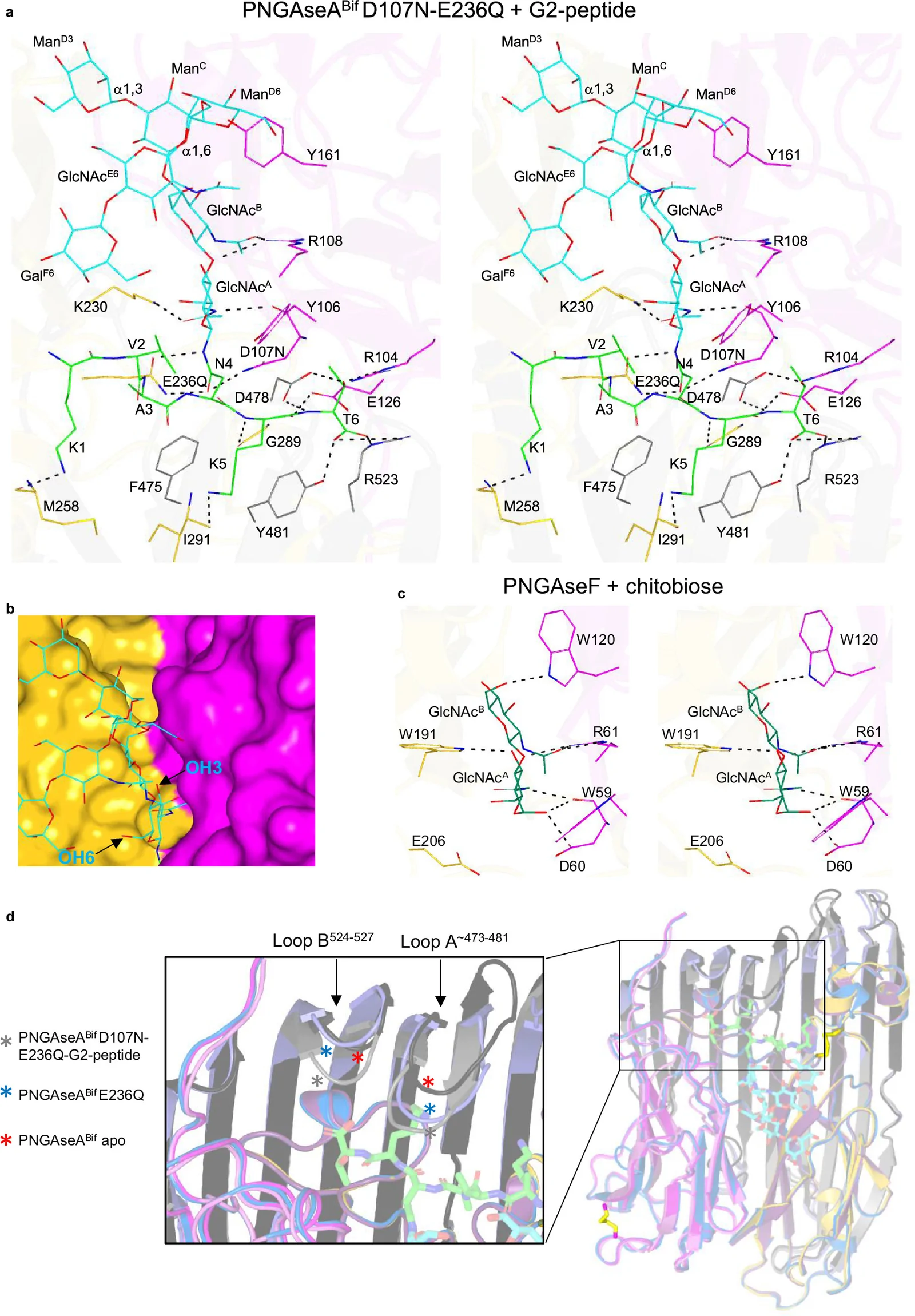
Details of the G2-peptide binding site and structural-snapshots of the N-Glycopeptide-induced β-sheet cradle closure. **a** Stereo close-up view of the active site in the PNGaseA^Bif^ D107N–E236Q–G2-peptide complex. Selected enzyme residues are shown as sticks, and black dashed lines indicate hydrogen bonds. Recognition is centred on the chitobiose core and the central mannose, together with peptide contacts around the glycosylated Asn and the Thr residue at the +2 position. Colours are as in Fig. 3. **b** Surface representation of the PNGaseA^Bif^ binding site showing that the OH3 and OH6 groups of the innermost GlcNAc remain solvent-exposed. The surface is coloured as in Fig. 3, highlighting the open environment around the chitobiose core and its capacity to accommodate core substitutions without major steric clash. **c** Stereo close-up comparison with PNGaseF bound to chitobiose, highlighting the corresponding binding site for the two core GlcNAc residues. Equivalent PNGaseF residues involved in chitobiose recognition are shown for comparison, revealing conservation of key interactions with the chitobiose core together with differences in the surrounding binding-site architecture. **d** Superposition of apo PNGaseA^Bif^, the unliganded E236Q mutant and the D107N/E236Q–G2-peptide complex, revealing ligand-induced conformational changes. The inset highlights the movements of cradle loop A (residues ~473–481) and loop B (residues 524–527) upon glycopeptide binding.

To begin to define the molecular basis by which PNGaseA^Bif^ recognizes N-glycopeptides through a substrate-enzyme complex, we generated a single and a double mutant based on previous investigations into the active site residues of PNGaseF (Fig. 3 and Supplementary Information). Through this we produced a ligand-free E236Q single mutant (29JO) and a D107N–E236Q double mutant crystallized with a glycopeptide substrate (29JQ; Fig. 4). The ligand used for these experiments was a short glycopeptide KVANKT, in which Asn4 carries a G2 N-glycan, that is, a biantennary complex-type N-glycan terminating in two galactose residues. MALDI-TOF analysis showed that the E236Q single mutant retained partial activity, generating product ions consistent with deglycosylated KVADKT detected as protonated and sodiated species (m/z ~ 661.7 and 683.7), whereas the D107N–E236Q double mutant was dominated by intact G2-peptide (m/z ~ 2306.6), identifying the latter as the appropriate construct for trapping a substrate-bound state (Supplementary Information and Supplementary Fig. 14).

The PNGaseA^Bif^-D107N–E236Q–G2-peptide complex is the first PNGase structure to be crystallised with a glycopeptide and provides the first insights into the recognition of both glycan and peptide by the PNGase superfamilies (Fig. 3 & 4, Supplementary Fig. 15). Furthermore, the comparison of the three structures presented here, demonstrates that the D107N and E236Q substitutions neutralize the catalytic pair while largely preserving the geometry of the side chains flanking the scissile amide, thereby enabling capture of a substrate-relevant bound state. Clear electron density for the bound G2-peptide was observed, allowing detailed placement of the ligand in the active site; however, density was not observed for the GlcNAc and terminal galactose on the α1,3 arm (Supplementary Fig. 15). Notably, in this complex, the C-terminal region of the peptide is markedly buried within the positively charged cleft formed between the catalytic core and the β-sheet cradle, suggesting that substrate engagement extends beyond glycan recognition and involves substantial enclosure of the peptide moiety (Fig. 4 and Supplementary Fig. 15). A surface representation of PNGaseF with the PNGaseA^Bif^-bound G2-peptide superposed shows that the peptide would remain substantially more solvent-exposed than in PNGaseA^Bif^, providing a structural explanation for why PNGaseF is better suited to accommodate N-glycans in larger glycoprotein contexts, whereas PNGaseA^Bif^ is adapted for N-glycopeptide recognition (Supplementary Fig. 16).

### The chitobiose core and central mannose are the key glycan components recognised

Analysis of the substrate-enzyme complex reveals that recognition of the glycan is dominated by the chitobiose core and the central mannose (Fig. 4). The innermost GlcNAc (A^G2^) is anchored by contacts from both backbone and side-chain atoms of the enzyme. In particular, the backbone of residue Asn107 contacts the acetamide nitrogen of A^G2^, whereas the side chain of Lys230 contacts the acetamide oxygen and the side chain of Arg108 contacts OH4 of the same sugar. A^G2^ is further stabilized by a CH–π interaction with the side chain aromatic ring of Tyr106. MD simulations of the complex between PNGaseA^Bif^ and the G2-peptide, after reverting D107N and E236Q mutations, reveal that Asp107 also forms stable interactions with A^G2^ acetamide nitrogen (Supplementary Fig. 16). The second GlcNAc (B^G2^) is recognized mainly through the side chain of Arg108, which contacts its acetamide group, and by an additional CH–π interaction with the side chain aromatic ring of Tyr161. Interactions between B^G2^ and Arg108 are also maintained in MD simulations, while interactions with Tyr161 are substituted by Tyr106 (Supplementary Fig. 16). Tyr161 also packs against the central mannose (C^G2^), further stabilizing the conserved glycan core. By contrast, the more distal sugar moieties do not interact with the enzyme and are solvent-exposed (Fig. 4). Additionally, OH3 and OH6 groups of A^G2^ remain accessible to solvent, indicating that substitutions at these positions, including core α1,6-fucose, can likely be accommodated without major steric clash. Consistent with this, modelling of core α1,6-fucose and core α1,3-fucose, the latter a hallmark of plant N-glycans and also common in insect N-glycans^32^, places both fucose moieties in a solvent-exposed position without obvious interference with the protein (Supplementary Fig. 17). Together, these observations indicate that glycan recognition by PNGaseA^Bif^ is concentrated on the conserved chitobiose core and central mannose, while the surrounding region remains structurally permissive to substitution.

### Recognition of the peptide is predominantly driven by the asparagine and + 2 threonine

Recognition of the peptide is concentrated around the glycosylated Asn and, especially, the +2 Thr of the NXT sequon present in the N-glycopeptide (Fig. 4 and Supplementary Fig. 15). In the captured complex, the side chain of Gln236 forms hydrogen bonds with both the side-chain amide nitrogen and side chain carbonyl oxygen of Asn4, thereby positioning the scissile amide at the catalytic centre. On the opposite side, the side chain amide of Asn107 contacts the side-chain carbonyl oxygen of Asn4, likely preserving the geometry of the catalytic pair in the mutant complex. Additional recognition involves the peptide backbone around Lys5: the side chain of Glu126 contacts the backbone carbonyl oxygen of Lys5, the side-chain amine of Lys5 forms a hydrogen bond with the backbone carbonyl oxygen of Ile291, whereas the backbone carbonyl of Gly289 contacts the backbone amide nitrogen of Lys5. The N-terminal region of the peptide is also contacted by the backbone carbonyl of Met258, which interacts with the side-chain of Lys1. The most extensive peptide recognition is centred on Thr6, the +2 residue of the sequon. Here, the side chain of Asp478 forms one hydrogen bond with the backbone amide nitrogen of Thr6 and a second with its side-chain hydroxyl group. The side chain of Arg104 also contacts the side-chain hydroxyl of Thr6. In addition, the side chain of Arg523 forms hydrogen bonds with the backbone carbonyl oxygen and the terminal carboxylate of Thr6, while the side chain hydroxyl of Tyr481 also contacts the terminal carboxylate. Tyr106 further contributes through a CH-π interaction with the Thr6 side-chain methyl group. Together, these interactions indicate that PNGaseA^Bif^ does not simply bind a glycan-bearing peptide but selectively reads the Asn and +2 Thr residues of the NXT sequon in the captured complex.

### The accommodation of core α-fucosylated plant and insect N-glycans

Comparison with PNGaseF highlights both the conserved and distinctive features of glycan recognition (Figs. 3 and 4, and Supplementary Fig. 12). In PNGaseF, the chitobiose core is recognized by Asp60, Arg61, Trp59 and Trp120, whereas the equivalent recognition platform in PNGaseA^Bif^ is formed by Asn107, Arg108, Tyr106 and Tyr161. Thus, the basic mode of chitobiose recognition is conserved between the two enzymes, even though PNGaseA^Bif^ uses tyrosines rather than tryptophans for the two principal CH–π interactions. A key difference lies near the second GlcNAc, where PNGaseF contains Glu118, which has been implicated in excluding N-glycans carrying core α1,3-fucose^31^, whereas PNGaseA^Bif^ contains Thr159. The smaller threonine side chain creates a more open environment around the OH6 of B^G2^ and the OH3/OH6 region of A^G2^, providing a structural explanation for why PNGaseA^Bif^ can accommodate core-fucosylated substrates. This is consistent with our biochemical observation that PNGaseA^Bif^ releases N-glycans from horseradish peroxidase, whose glycans predominantly carry core α1,3-fucose decorations.

Structural models generated with Protenix of PNGase^Bif‘^ in complex with G2-peptides carrying core α1,3- or α1,6-fucose are also consistent with this interpretation, as the fucose is accommodated without steric clashes in the active-site region. In both models fucose remains largely solvent-exposed, with only limited contacts, in the case of α1,3-fucose, with Lys230 (Supplementary Fig. 17). On the opposite side of the disaccharide, PNGaseA^Bif^ is also more open than PNGaseF. Whereas PNGaseF contains residues that pin the chitobiose core into the active site, PNGaseA^Bif^ lacks the equivalent constraining environment, further supporting its ability to accommodate differently decorated cores. As noted above, PNGaseF lacks the additional β-sheet cradle, and the superposed G2-peptide therefore remains much more solvent-exposed (Supplementary Fig. 15), consistent with its more open substrate-binding architecture. These structural and biochemical data show that PNGaseA^Bif^ can accommodate core α1,3- and α1,6-fucosylated N-glycans because the binding site remains open around the chitobiose core while preserving core glycan recognition.

### N-Glycopeptide-induced β-sheet cradle closure in PNGaseA^Bif^ is supported by molecular dynamics simulations

Comparison of the three crystal structures of PNGaseA^Bif^ shows that the most prominent conformational differences are localized around the substrate-binding cleft. In the apo and ligand-free E236Q structures, the region between the catalytic core and the β-sheet cradle remains relatively open. In contrast, in the D107N–E236Q–G2-peptide complex, glycopeptide binding is associated with closure of the β-sheet cradle toward β-sandwich 1, generating a more enclosed binding site over the peptide portion of the substrate (Fig. 4 and Supplementary Fig. 15). This transition is driven primarily by movements in two β-sheet cradle segments, loop A (~residues 473–481) and loop B (residues 524–527), which shift toward the bound peptide and define a glycopeptide-induced cradle-closure mechanism. Furthermore, B-factors in the apo structure suggest that the cradle loops near the active site are dynamic, consistent with a binding region that can open and close to accommodate substrates of different sizes (Supplementary Fig. 18).

To further examine this open-to-closed transition, we performed molecular dynamics simulations on the wild-type enzyme in complex with the G2-peptide and after ligand removal. Whereas the peptide-bound enzyme remained in a more compact state, removal of the G2-peptide led to retraction of the cradle loops toward a more open conformation, consistent with the structural snapshots provided by the apo and E236Q structures (Supplementary Fig. 19). The closed state is further stabilized by contacts between the β-sheet cradle and β-sandwich 1 around the peptide-binding region (Supplementary Fig. 15). In particular, accommodation of the C-terminal portion of the peptide appears to promote additional direct interactions between these two domains. For example, the side chain of Asp478 in the cradle forms a hydrogen bond with the hydroxyl group of Tyr166 in β-sandwich 1, whereas the side chain of Arg104 in β-sandwich 1 forms a hydrogen bond with the side-chain carbonyl group of Asn479 in the β-sheet cradle. Together, the three crystallographic states, apo, ligand-free E236Q and the D107N–E236Q–G2-peptide complex, show the cradle in increasingly closed conformations, and the molecular dynamics simulations are consistent with this open-to-closed transition, supporting a glycopeptide-induced cradle-closure mechanism. Although this model rests on structural and computational evidence, direct mutation of the individual cradle contacts would provide independent validation and is a clear objective for future work.

The molecular dynamics simulations also support the peptide interactions observed in the structure and help explain how cradle closure tightens the C-shaped pocket. Interactions with the backbone carbonyl oxygen and terminal carboxylate of Thr6 are stabilized during dynamics, while interactions with its side chain are less frequently maintained (Supplementary Fig. 16). Thus, recognition of the +2 Thr involves both backbone and side chain features of the peptide and draws contributions from both β-sandwich 1 and the β-sheet cradle. These interactions explain why the C-terminal portion of the peptide becomes buried upon cradle closure and identify the +2 Thr as the dominant peptide recognition element in the captured complex. This is reminiscent of the importance of the +2 Ser/Thr residue in substrate recognition by oligosaccharyltransferases and soluble bacterial N-glycosyltransferases^12,13^. At the same time, the active-site cleft remains sufficiently open that longer peptide substrates could also be accommodated. This dynamic cradle-closing mechanism explains how glycopeptide binding encloses the peptide portion of the substrate and stabilizes the binding cleft of PNGaseA^Bif^.

### AlphaFold3 models indicate that a variety of protein sequences could be accommodated in the C-shaped pocket

To test the potential for the C-shaped pocket to accommodate a variety of peptides, AlphaFold3 was used to model different peptides (Supplementary Fig. 20). The results indicate that the negatively charged end of the pocket (formed by β-sandwich 2 loop 2) accommodates the N-terminus of the peptide and a maximum of three or four residues at the N-terminus would allow the correct positioning of an asparagine in the active site. Furthermore, the C-terminal part of the peptide was accommodated in the positively charged part of the C-shaped pocket and threaded through to the open-end of this pocket.

### Two catalytic mechanisms remain plausible for the PNGase families

**Fig. 5:**
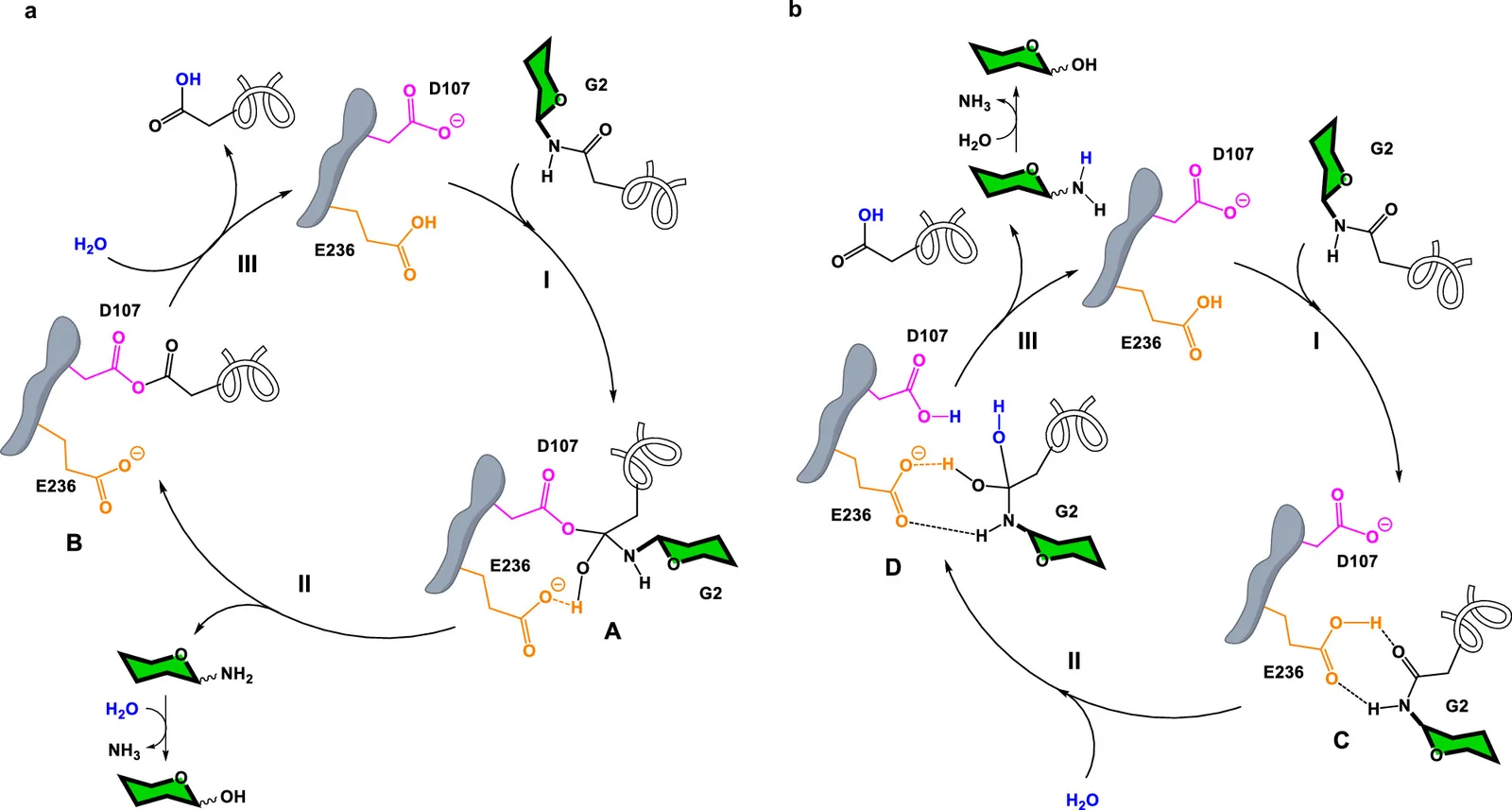
Proposed catalytic mechanisms for PNGaseA^Bif^-mediated N-glycan cleavage from N-glycopeptides. **a** In the direct-attack mechanism, D107 performs a nucleophilic attack on the side-chain amide carbonyl of the glycosylated Asn in the G2-peptide, generating tetrahedral intermediate **A** (step I). This intermediate subsequently evolves to form anhydride **B** (step II), thereby completing a classical carbonyl nucleophilic substitution process. The leaving glycosylamine is spontaneously hydrolysed in aqueous medium to yield the corresponding free carbohydrate. Finally, in step III, intermediate **B** undergoes hydrolysis in aqueous medium, releasing the peptide and restoring the enzyme to its initial state. **b** In the water-mediated mechanism, binding of the G2-peptide generates the enzyme–substrate complex **C** (step I). D107 acts as a general base to activate a water molecule for nucleophilic attack on the side-chain amide carbonyl, while E236 contributes to polarization of the scissile amide bond. This generates tetrahedral intermediate **D** (step II), which subsequently collapses to complete amide hydrolysis (step III), releasing the deamidated peptide and the glycosylamine. As in (**a**), the glycosylamine is spontaneously hydrolysed in aqueous medium to produce the free carbohydrate.

The substrate-bound complex also provides insight into the catalytic mechanism. The scissile Asn is positioned between residues 107 and 236, corresponding in the wild-type enzyme to Asp107 and Glu236. In the D107N–E236Q–G2-peptide complex, the OD1 atom of Asn107, which approximates the position of one Asp107 carboxylate oxygen after reversion to the wild-type residue, is 3.2 Å from the carbonyl carbon of the scissile amide. This distance places residue 107 within a range compatible with direct nucleophilic attack. Because the Asn and Gln substitutions are expected to preserve the overall hydrogen-bonding geometry of the native acidic residues, this mutant complex likely represents a reasonable approximation of the substrate-bound catalytic arrangement. Computational analysis using the Protoss server^33^, further suggests that, in the wild-type enzyme, Glu236 is protonated whereas Asp107 remains deprotonated, consistent with a division of labour in which Glu236 polarizes the scissile amide carbonyl and Asp107 carries out the key catalytic chemistry. To assess the structural stability of the substrate-bound complex and probe the participation of Asp107 in catalysis, MD simulations of 500 ns were performed on the PNGaseA^Bif^-G2-peptide complex after reverting the D107N and E236Q mutations to the wild-type residues. Backbone RMSD of the protein remained consistently stable across all replicas (Supplementary Fig. 10a). The distance between the Asp107 carboxylate oxygen and the carbonyl carbon of the scissile amide was monitored to test the catalytic competence of a direct nucleophilic attack (Supplementary Fig. 10b). Although this distance tends to increase during the early phases of the simulations, it transiently visits values of ~3–4 Å, within the expected range for this attack to occur (~3$\,{\mathrm{\AA}}$). Consistent with this interpretation, the E236Q substitution does not abolish activity, supporting a primary role for Glu236 in substrate polarization rather than direct catalytic turnover. In the most widely accepted scenario, Asp107 would act as a general base to activate a water molecule for nucleophilic attack on the amide carbonyl. However, because no catalytic water is observed in the present structure, an alternative mechanism in which Asp107 participates more directly in catalysis cannot be excluded. Thus, the current structural and computational data support two plausible catalytic scenarios (Fig. 5), both involving polarization of the substrate amide by Glu236 and a central catalytic role for Asp107, while leaving the precise chemical pathway to be resolved by future ab initio/MD studies and experimental validation.

### The β-sheet cradle is predicted to be conserved throughout the PNGaseA superfamily

To assess the conservation of these structural features across the family, we analysed sequence conservation using ConSurf (Supplementary Fig. 21). The highest conservation maps to the active site and to two regions flanking it where the β-sheet cradle meets the catalytic module. On one side, this conserved patch contains two cysteines that do not form a disulphide bond in the present structure (Supplementary Fig. 22), whereas on the other side it contains two highly conserved arginine residues that generate the positive charge at the entrance to one of the pockets. Notably, the C-shaped double pocket of PNGaseA^Bif^ is formed at the interface between the catalytic module and the β-sheet cradle, suggesting that the presence of this sheet in other putative structures may be associated with a preference for N-glycans attached to short peptides. Consistent with this idea, structural models of PNGaseA homologues from fungi, bacteria, archaea, oomycetes and plants predict that both the catalytic module, composed of two eight-stranded β-sandwiches, and the large β-sheet cradle are broadly conserved features across the family (Supplementary Fig. 23). Differences are limited to local variations, including a large extra loop in the *Candida viswanathii* structure, an incomplete cradle in the *Cordyceps confragosa* structure, and a small additional module in the putative PNGaseA from *Bifidobacterium leontopitheci*. Together, these analyses suggest that the β-sheet cradle is a conserved family feature likely linked to N-glycopeptide recognition.

### Carbohydrate-Active enZyme conservation across *B. bifidum* strains

The presence of a gene encoding a PNGaseA appears to be relatively uncommon among members of the *Bifidobacterium* genus from the searches carried out during this work. To understand the wider biological context of where PNGaseA^Bif^ fits into the glycan breakdown capacity of *B. bifidum* LMG13195, the conservation of other Carbohydrate-Active enZyme (CAZyme) families was assessed across different strains with different origins of isolation (Supplementary Fig. 24). The presence of putative GH family members, their frequency within a genome, and the conservation of different GH enzymes across different strains was assessed for host-associated CAZyme families (Supplementary Table 3). Overall, the frequency of a given GH family is consistent across strains, especially for host-associated enzyme families. The most striking aspect of this analysis was the very high conservation of CAZymes between strains for the different CAZyme primary sequences (Supplementary Table 3). Those enzymes that were assessed had no less than 90 % identity.

The highest represented GH family is 13, with between six and ten putative enzymes. These enzymes likely degrade starch and other α-glucan polysaccharides, in addition to roles in energy storage within the cell. No GH13 enzymes from *B. bifidum* are currently characterised. The second most prevalent family is GH20 with a consistent four enzymes across strains and >90 % identity in protein sequence. This family often plays a critical role in host glycan degradation in human commensal microbes, and, for example, it is the largest represented GH family in the mucinophilic human colonic commensal *Akkermansia mucinophila*^34^. The GH20 family members typically have β-HexNAc’ase activities and those that have been characterised for *B. bifidum* show distinct specificities^35–38^. For the other host-associated GH family members, there is typically only a single highly conserved enzyme, for example GH29, GH89, GH95 and GH110.

### A model of host glycan breakdown by *B. bifidum* LMG13195

**Fig. 6:**
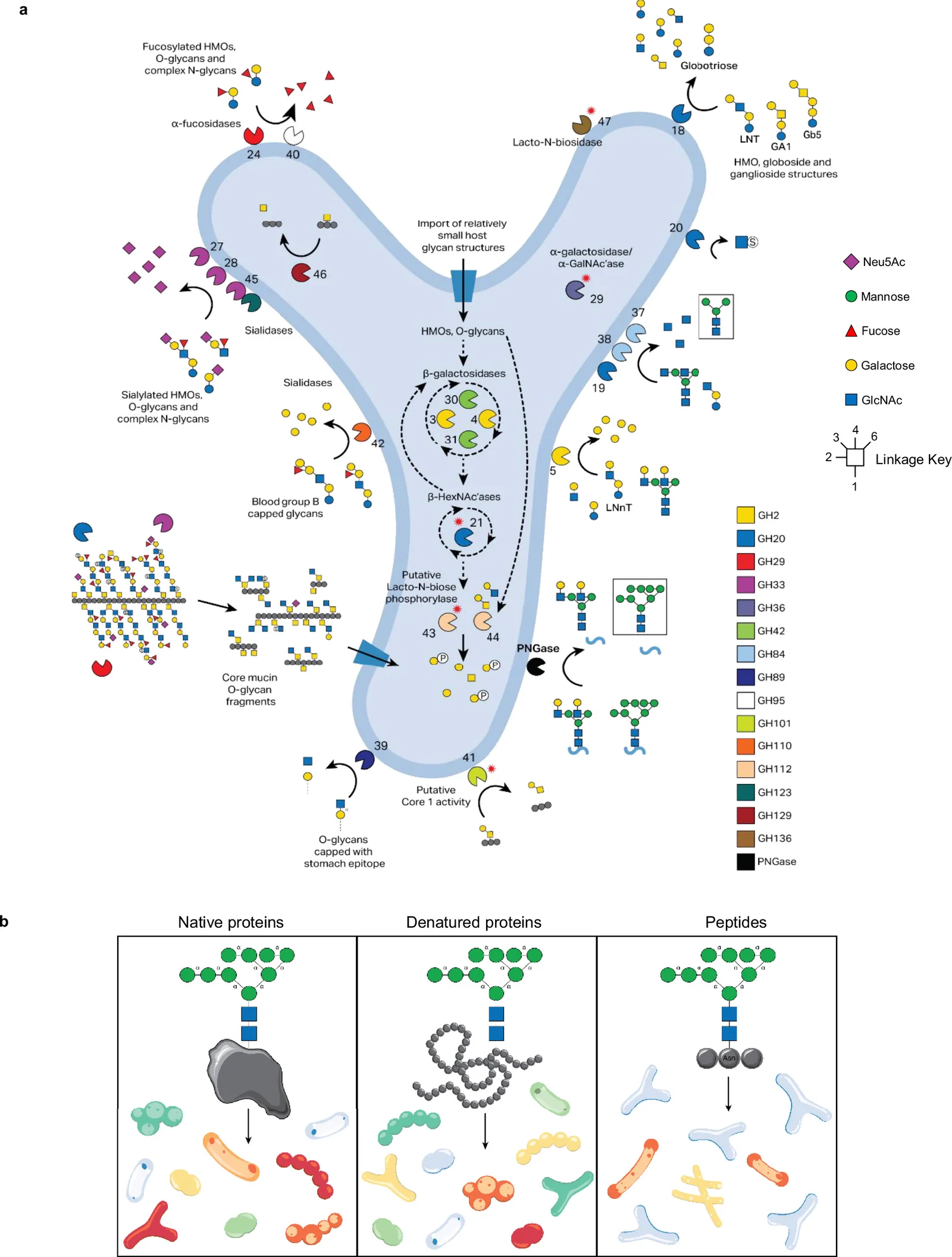
A model for the breakdown of host glycans by *Bifidobacterium bifidum* LMG13195. **a** This model includes the breakdown of human milk oligosaccharide structures, O-glycans, N-glycans and complex glycosphingolipid structures. The information is predominantly taken from the literature, the localisation follows DeepLocPro, and the red twelve-pointed stars indicate where biochemical characterisations are currently unknown. The enzymes are coloured according to their family and further information is available in Supplementary Table 3. **b** A model for how the state of the protein component of a glycoprotein drives enzyme specificity and, therefore, directs nutrition to different microbial species. Microbial species expressing PNGaseA-type enzymes will likely be primarily be accessing N-glycans on peptides (right panel). Whereas N-glycans linked to denatured proteins (central panel) or native proteins (left panel) will likely be accessed by microbes expressing PNGaseF superfamily members or GH18/85 family members, respectively.

To construct a model detailing how *B. bifidum* LMG13195 breaks down different host glycans, we used a combination of cellular localisation prediction and current literature (Fig. 6 and Supplementary Table 3). *B. bifidum* strains have many of their CAZymes predicted to be on the cell surface, and these likely act to trim down large host glycans to substrates that can be imported for further breakdown by CAZymes residing intracellularly. A range of both exo- and endo-acting CAZymes are predicted to be on the surface.

In terms of exo-acting enzymes, there are three α-sialidases (GH33), two α-fucosidases (GH29 and GH95), an α-galactosidase (GH110), a β-galactosidase (GH2), four β-HexNAc’ases (two GH20 and two GH84), a α1,4-GlcNAc’ase (GH89), and a recently characterised β-GalNAc’ase (GH123) predicted to be on the surface^39^. This high number of activities compartmentalised to outside the cell is notable and may reflect the import machinery playing a selective role here. Many of these enzymes have had some level of specificity defined, which means that a fairly accurate glycan utilisation model can be proposed for the CAZymology taking place on the surface of *B. bifidum*. The three CAZymes predicted to act first will be the GH33, GH89, GH110 enzymes that will remove α-linked sialic acid, α1,4-GlcNAc, and blood group B α-galactose capping decorations^40–44^, respectively. Sialic acid decorates many different host glycans and blood group B is present in 0-30 % of the population, depending on genetics. The α1,4-GlcNAc decoration is common on stomach-produced mucins that travel into the colonic environment continuously. Then, the two GH29 and GH95 enzymes have been shown to act on α1,3/4- and α1,2-linkages, respectively, and human milk oligosaccharides, N-glycans and O-glycans are all commonly decorated with α-fucose^45,46^. The GH2 β-galactosidase has been characterised as having β1,4 specificity and demonstrated to act on Lacto-N-*neo*tetraose, lactose and N-glycans^47^. The one GH20 (BBJK_00776/BbhI) and two GH84 family members have been shown to have β-GlcNAc’ase activity against a wide variety of host substrates, including against N-glycans^36,37^. The other GH20 (BBJK_03032/BbhII) has specificity for 6S-GlcNAc common to mucins^38^. Finally, the GH123 characterised as having β-GalNAc activity against Sda antigen structures common in the colonic environment and common to ganglioside and globoside structures, but also present in O- and N-glycans^39^.

The endo-acting CAZymes predicted to be on the surface of the cell include a characterised Lacto-N-biosidase from the GH20 family and a putative GH136 family member. The GH20 can remove β1,3 disaccharides from Lacto-N-tetraose, ganglioside GA1 and globoside Gb5^35^. The products are likely then imported for further degradation, although the globotriose product from the Gb5 may be further broken down by the GH110. The substrates for these endo-acting enzymes are common human milk oligosaccharide and O-glycan structures. A putative GH101 is also predicted to be present, and this family is characterised predominantly to remove core 1 Galβ1,3GalNAc disaccharides from mucin glycoproteins^48,49^. Finally, in the case of *B. bifidum* LMG13195, the PNGaseA is predicted to also be on the surface and target N-glycans on short peptides which can then be sequentially broken down by the GH2 and GH84 enzymes. *B. bifidum* genomes do not encode GH families with α-mannosidase activities, so it is hypothesised that the core parts of these N-glycans are not accessed as a nutrient source but are instead cross-fed to other members of the microbiota.

Once the host glycans are imported into the cell, it is hypothesised that they can be broken down further using the four β-galactosidase (2xGH2 and 2xGH42) and uncharacterised GH20^36–38,47^. There are also two putative GH112 Lacto-N-biose phosphorylases that likely act in parallel. These two GH112 enzymes have ~ 85 % identity between them and both are highly conserved over the strains assessed. A GH129 is also predicted to be inside the cell and has been characterised as removing GalNAc from mucin glycopeptides^50^. Finally, a GH36 is also predicted to be localised intracellularly and this family has been shown to have α-galactosidase/GalNAc’ase activities. It would be logical that this enzyme is an α-GalNAc’ase, used to break-down blood group A structures, but specificity is still to be defined^34^.

## Discussion

The colonisation of the infant colon by species of *Bifidobacterium* plays a vital and holistic role in the development of a healthy gut microbiota. A full mechanistic understanding of how these microbes colonise so successfully, particularly in the presence of breast milk, has yet to be fully elucidated. One of the main factors driving successful colonisation are the human milk oligosaccharides found in breast milk, but other factors, including N-glycans, also influence this process^51^.

In this study, we characterise a PNGaseA superfamily member from *B. bifidum* LMG13195 and show that it preferentially acts on N-glycans attached to short peptides rather than to denatured or native glycoproteins. At the same time, PNGaseA^Bif^ accommodates a broad selection of different N-glycan structures, including high-mannose, mammalian complex, and simple plant N-glycans with core α1,3-fucose. Within the broader host-glycan utilisation network of *B. bifidum* LMG13195 (Fig. 6), PNGaseA^Bif^ therefore emerges as one component of a wider glycan-foraging strategy that extends beyond human milk oligosaccharides to O-glycans, N-glycans and glycosphingolipids. Importantly, our biochemical data show that access to N-glycans is influenced not only by glycan composition, but also by the physical state of the protein carrier. In this context, the model in Fig. 6 supports the idea that cradle-bearing PNGaseA enzymes are best suited to N-glycopeptides, whereas denatured or native glycoprotein substrates are more likely to be processed by other N-glycan-releasing enzyme classes. In the case of colonic-dwelling *B. bifidum*, the protein component of a glycoprotein would likely first be degraded either by host digestive enzymes or by proteolytic members of the microbiota, thereby generating N-glycopeptides that can then serve as substrates for PNGaseA^Bif^.

The major advance of this work is that PNGaseA^Bif^ defines a structurally and functionally distinct class within the PNGaseA superfamily. Rather than acting as a generic PNGaseA that merely tolerates a broader glycan repertoire, PNGaseA^Bif^ is specialized for N-glycopeptide recognition. This specialization is encoded by the β-sheet cradle, which emerges here as the defining architectural feature of this class. The cradle is not a passive appendage to the catalytic core but an active structural element that, together with the catalytic module, forms a C-shaped binding environment which partially encloses the peptide portion of the substrate and couples binding to cradle closure. This architecture does not simply broaden substrate tolerance but provides a structural basis for selective recognition of peptide-linked N-glycans. The structure thus explains why PNGaseA^Bif^ prefers N-glycopeptides over larger glycoprotein substrates and supports the idea that cradle-bearing PNGaseA enzymes represent a dedicated N-glycopeptide-processing class.

The substrate-bound complex further shows that recognition is not driven primarily by distal glycan features, but by the conserved chitobiose core together with specific reading of the peptide context. Most notably, recognition is concentrated around the glycosylated Asn and the +2 Thr residue of the bound N-glycopeptide. Since the bound glycopeptide carries a Thr at the +2 position, our data define recognition of an NXT motif, leaving open whether Ser-containing NXS sequons are recognised in the same way. In the captured complex, the +2 Thr makes the most extensive peptide contacts, involving residues from both β-sandwich 1 and the β-sheet cradle. This indicates that PNGaseA^Bif^ does not simply recognize a glycan on a peptide, but instead reads key elements of the canonical N-glycosylation sequon itself. This suggests that the same NXS/T logic that guides N-glycan installation by oligosaccharyltransferases and soluble bacterial N-glycosyltransferases may also be exploited here during N-glycan removal, although this is based on a single NXT glycopeptide and would need additional substrates to confirm for NXS sequons. Cradle-bearing PNGaseA enzymes therefore appear adapted not only to glycopeptides in general, but specifically to N-glycopeptides carrying sequon features that can be productively engaged in the active site.

The structural data also provide a mechanistic framework for catalysis. The trapped D107N–E236Q complex places the scissile Asn between the two catalytic residues and, together with the computational analysis, supports a division of labour in which Glu236 polarizes the scissile amide carbonyl whereas Asp107 carries out the key catalytic chemistry. Two mechanistic scenarios remain plausible. In the most likely pathway, Asp107 acts as a general base to activate a water molecule for nucleophilic attack on the amide carbonyl, while Glu236 enhances electrophilicity of the scissile bond through amide polarization. However, because no catalytic water is observed in the present structure, an alternative route in which Asp107 participates more directly in catalysis cannot yet be excluded. Taken together, the available data favour a water-mediated mechanism in which Asp107 acts as the principal catalytic base and Glu236 primarily contributes by polarizing the substrate amide, while still leaving open the possibility of a more direct catalytic role for Asp107. This establishes a much more precise mechanistic framework for PNGaseA catalysis than was previously available.

Comparison with PNGaseF reinforces the idea that PNGases have evolved distinct architectural solutions for different substrate niches. PNGaseF retains the conserved core logic of chitobiose recognition but lacks the β-sheet cradle and presents a more open substrate-binding environment, consistent with processing N-glycans in larger glycoprotein contexts. By contrast, PNGaseA^Bif^ combines a conserved catalytic platform with cradle-mediated peptide recognition and active-site enclosure, thereby adapting the enzyme to N-glycopeptide substrates. These comparisons support a model in which distinct classes of N-glycan-releasing enzymes are matched to distinct substrate states, with cradle-bearing PNGaseA enzymes occupying the N-glycopeptide niche.

Finally, a glycan utilisation model for *Bifidobacterium bifidum* LMG13195 was constructed and provides a broader view of how this species may access host-derived glycoconjugates in the infant gut. This model underlines what is known about the glycobiology of this species and what remains to be understood. Overall, our findings indicate that N-glycan acquisition in infant-associated *Bifidobacterium* is governed not only by glycan composition, but also by the structural context in which the glycan is presented. Within this broader host-glycan foraging network, PNGaseA^Bif^ represents a specialized solution for N-glycopeptide processing in which the β-sheet cradle enables selective recognition, peptide enclosure and deglycosylation of peptide-linked substrates. Together, these results define a cradle-bearing class of PNGaseA enzymes that couples sequon recognition, active-site closure and catalysis, thereby expanding the nutrient landscape accessible to infant gut *Bifidobacterium* species.

## Methods

### Bioinformatics

Initially, annotated PNGase enzymes were collated using Interpro, Uniprot and IMG databases^19–21^. This provided two candidates from *Bifidobacterium bifidum* LMG13195 and *Bifidobacterium leontopitheci*. Due to the presence of a PNGase being relatively uncommon to species of *Bifidobacterium*, the genetic context was then investigated. To analyse the variable region containing the gene encoding PNGaseA^Bif^ (*pngaseA*^*bif*^) across multiple *B. bifidum* genomes, the genbank files for 18 complete *Bifidobacterium bifidum* strains were downloaded from the National Centre for Biotechnology Information (NCBI) website. The initial search was filtered to include only complete genomes, resulting in the selection of the 18 genomes included in the analysis (accessed on 4th July 2025). The downloaded genbank files were uploaded to artemis (v16.0.5) and gene clusters were manually curated by searching for the actetate kinase and phosphoketalase genes, which flank the desired cluster. Once located, gene clusters were saved as a genbank file and uploaded to cagecat clinker1 for analysis^52^. The output graph was then refined to show only the unique clusters based on visual similarity and the number of strains found to contain each cluster was recorded on the figure. A detailed summary of all genomes analysed can be found in Supplementary Table 3. AlphaFold3 was used to model interactions between PNGaseA^Bif^ and different peptides^53^.

Putative signal sequences were identified using DeepLocPro 1.0^25^. The identities between different protein sequences were determined using Clustal Omega using full sequences^54^. The CAZy database was used to assess the GH families present in different *B. bifidum* strains^55^.

### Cloning and recombinant expression

All recombinant proteins were heterologously expressed in *Escherichia coli*. PNGaseA^Bif^ was produced using two construct/strain combinations. In one, the gene *pngaseA*^*bif*^ was amplified from genomic DNA and cloned into pET28b (Novagen) using primers ATTATACATATGGATAACACAATCGCCGGGCATGCCG ATTATACTCGAGCGATGTCTGAGCTGAAGTATGCTTAATCACC and restriction sites NdeI and XhoI. In the other, codon-optimised DNA encoding PNGaseA^Bif^ and its mutants was synthesised for expression in *E. coli* and cloned into pMALC5x. The pET28b-derived PNGaseA^Bif^ was used for enzymatic and kinetic assays and to obtain the apo structure, whereas pMALC5x-derived wild-type and mutant proteins were used for enzymatic, kinetic and structural studies.

For the pET28b construct, *pngaseA*^*bif*^ was cloned using the NdeI and XhoI restriction sites and transformed into *E. coli* TUNER cells (Novagen). Cells were grown in Luria–Bertani (LB) medium supplemented with 50 μg/mL kanamycin at 37 °C to mid-exponential phase, cooled to 16 °C, induced with 0.2 mM isopropyl β-D-1-thiogalactopyranoside (IPTG), and incubated overnight at 16 °C with shaking. Cells were harvested and recombinant His-tagged proteins were purified from clarified extracts by immobilised metal affinity chromatography (IMAC) using 20 mM Tris-HCl, 300 mM NaCl, pH 8.0. BT3990 and BT0455 from *Bacteroides thetaiotaomicron* and PNGaseL from *Flavobacterium akiainvivens* were expressed and purified using the same protocol^2,28,29^.

For the pMALC5x construct, codon-optimised DNA sequences encoding PNGaseA^Bif^and its mutants were synthesised by Tsingke (China) with 5′ SalI and 3′ EcoRI sites followed by a stop codon. PNGaseA^Bif^ was cloned into pMALC5x to generate pMALC5x-12His-TEV-*pngaseA*^*Bif*^, containing a Tobacco Etch Virus (TEV) protease cleavage site between MBP and the protein of interest. Mutants were generated by site-directed mutagenesis from this construct. Plasmids were transformed into *E. coli* BL21(DE3) cells and grown at 37 °C in 2×TY medium supplemented with 100 μg/mL ampicillin. At an OD600 of 0.6–0.8, expression was induced with 1 mM IPTG and cultures were incubated for 16 h at 18 °C. Cells were harvested, resuspended in buffer A (25 mM Tris-HCl, pH 7.5, 500 mM NaCl, 10 mM imidazole), and clarified lysates were loaded onto a HisTrap column (GE Healthcare). Proteins were eluted with an imidazole gradient and dialysed into buffer C (25 mM Tris-HCl, pH 7.5, 150 mM NaCl). The affinity tag was removed by incubation with TEV protease at a 1:50 TEV:protein ratio for 20 h at 18 °C, and TEV protease and the cleaved His-MBP tag were removed by a second HisTrap step. For crystallographic studies, PNGaseA^Bif^ E236Q and PNGaseA^Bif^ D107N–E236Q were further purified by size-exclusion chromatography on a HiLoad 26/60 Superdex 75 column (GE Healthcare) equilibrated in buffer C. Proteins were concentrated using Amicon Ultra-15 centrifugal filters.

Protein purity and apparent molecular mass were assessed by SDS–PAGE using precast 4–16% gradient gels (Bio-Rad) stained with Coomassie Brilliant Blue (ReadyBlue® Protein Gel Stain, Sigma-Aldrich). Protein concentrations were determined from absorbance at 280 nm using theoretical molar extinction coefficients. For PNGaseA^Bif^ and its mutants, ε^280^ was 69,790–70,040 M^−1^ cm^−1^.

### Substrates

Bovine RNaseB, α1acid glycoprotein, fetuin and horseradish peroxidase were all purchased from sigma (R7884, G3643, F2379, and P6782). The G2 peptide for crystallography was purchased from TCI.

### N-glycopeptide substrate preparation

Denatured forms of the substrates were prepared by boiling for 10 minutes in a heat block. Partially degraded substrates were prepared by first feeding the substrate to *Vibrio coralliilyticus* ATCC BAA-450, which only utilises the polypeptide, leaving the N-glycan attached to peptide fragments. *Vibrio coralliilyticus* ATCC BAA-450 was grown from glycerol stocks in marine broth (Difco) at 30 °C with shaking at 180 rpm. The following day, cells (1:100 dilution) were transferred to 5 mL of 1:1 2X marine M9 (M9 supplemented with 15 g/L NaCl) and 2X substrate (20 mg/mL) and grown at 30 °C with shaking at 180 rpm for 24 h. Cultures were then centrifuged at 4500 rpm for 5 min to pellet cells. The resultant supernatant was retained, filter sterilised and the fragmented glycoprotein was purified using HyperSep™ Hypercarb™ SPE Cartridges (Thermo Fisher Scientific).

### Enzyme assays

Screening for activity was carried out against bovine RNaseB (R7884, Sigma-Aldrich), bovine α1acid glycoprotein (G3643, Sigma-Aldrich) and bovine fetuin (F2379, Sigma-Aldrich) in native, denatured and partially degraded/digested forms. Horseradish peroxidase (77332, Sigma-Aldrich) in native and denatured forms was also used. Assays were carried out in 20 mM 4-morpholinepropanesulfonic acid (MOPS), pH 7.0, at 37 °C, with a final glycoprotein concentration of 10 mg/mL. The final enzyme concentration was 5 μM. The SDS–PAGE gels were precast 4-16 % gradient (Bio-Rad) and either stained with Coomassie Brilliant Blue (ReadyBlue® Protein Gel Stain, Sigma) to visualise total protein or Schiff’s Fuchsin (Pierce Glycoprotein Staining Kit) to visualise just glycoprotein. Uncropped gel images are shown in Supplementary Fig. 25.

### Thin-layer chromatography

9 μL of recombinant enzyme and whole cell assays were spotted onto silica plates. The plates were resolved in running buffer containing butanol/acetic acid/water in a 2:1:1 and 1:1:1 ratio for high-mannose N-glycan substrates and complex N-glycan substrates, respectively. They were resolved twice, with drying in between, and stained using a diphenylamine-aniline-phosphoric acid stain^56^. Uncropped TLC images are shown in Supplementary Fig. 25.

### High-performance anion exchange chromatography-pulsed amperometric detection (HPAEC-PAD)

To analyse N-glycan release, samples were separated using a CarboPac PA300-4µm anion exchange column with a PA300G-4 µm guard using a Dionex ICS-6000 (Thermo Fisher) and detected using PAD. The flow was 0.25 mL min^−1^ and the buffers were A: water, B: 200 mM NaOH, C: 50 mM NaOH and 25 mM NaOAc, and D: 100 mM NaOH and 250 mM NaOAc. The elution conditions were 0-4 mins 80% A, 19.5 % B and 2% C, 4–20 mins had a linear increase of B and C to 20 and 60 %, respectively. This was followed by 20-50 mins where buffer D was increased linearly to 100 % and remained at that level from 50–60 min to clean the column. Software used was the Chromeleon Chromatography Data System. All data were obtained by diluting assays 1/10 in sterile MilliQ H_2_O prior to injection.

### MALDI-TOF mass spectrometry

The glycopeptide G2-peptide was incubated overnight at 37 °C at a final concentration of 0.5 mM with 50 µM of either PNGaseA^Bif^, PNGaseA^Bif^_E236Q or PNGaseA^Bif^_D107N-E236Q in 25 mM Tris-HCl, pH 7.5, 150 mM NaCl, in a final reaction volume of 100 µL. Reactions were terminated by heating at 100 °C and the products were analyzed by MALDI-TOF mass spectrometry using a Bruker NeoFlex instrument equipped with a SmartBeam™ Nd:YAG laser (~355 nm) and controlled by FlexControl 3.3 software (Bruker Daltonics). 2,5-Dihydroxybenzoic acid (DHB; 5 mg/mL in 70:30 ACN:water containing 0.1% TFA) was used as the matrix. One microlitre of reaction mixture was spotted onto an MTP 384 polished steel MALDI plate and allowed to dry, after which 1 µL of matrix was added and dried before acquisition. G2-peptide alone was included as a control. Data were analyzed using FlexAnalysis 3.3 software (Bruker Daltonics). MALDI-TOF MS analyses were performed in one or two independent experiments per condition, depending on the sample (*n* = 1–2). A total of four conditions were analysed: G2-peptide alone and G2-peptide incubated with PNGaseA^Bif^ WT, E236Q, or D107N–E236Q. No biological replicates were performed. The intact G2-peptide, released glycan, and deglycosylated peptide peaks were assigned based on their expected molecular masses and the observed m/z values. MALDI-TOF MS analyses were performed in one or two independent experiments per condition, depending on the sample (*n* = 1–2). A total of four conditions were analysed: G2-peptide alone and G2-peptide incubated with PNGaseA^Bif^ WT, E236Q, or D107N–E236Q. No biological replicates were performed. The intact G2-peptide, released glycan, and deglycosylated peptide peaks were assigned based on their expected molecular masses and the observed m/z values.

### Crystallisation, data collection and structure determination

For the apo structure, PNGaseA^Bif^ was subjected to a second purification step after IMAC by size-exclusion chromatography on a Superdex 200 26/200 column (Cytiva) using an ÄKTA pure FPLC system (GE Healthcare Life Sciences) equilibrated in 25 mM HEPES, 200 mM NaCl, pH 8.0. Fractions containing pure protein, as assessed by SDS–PAGE, were pooled and concentrated. PNGaseA^Bif^ was initially screened using commercial kits, using a sitting drop vapour diffusion method and set up using a Mosquito liquid handling robot (TTP Labtech). The crystallisation experiments were composed of 0.1 μL of reservoir solution and 0.2 μL of protein (25 mg/mL). A second drop of 0.1 μL reservoir solution was also included. The trays were sealed and incubated at 18 °C. Crystal clusters first formed in PACT condition 0.2 M NaI, 0.1 M Bis-tris propane, 20 % PEG3350 (w/v), pH 7.5. The clusters were fragmented via centrifugation with Microseed beads in the same reservoir solution that they formed. The seed stock was soaked in 10 mM chitobiose and was used to set up a fresh PACT screen. The crystal that diffracted successfully was formed in PACT condition 46–0.1 M Tris pH 8.0, 20 % PEG 6000 (w/v), 0.2 M Magnesium chloride. For cryoprotection, an additional 25 % PEG400 (w/v) was added to the crystallisation liquor prior to flash-freezing crystals in liquid nitrogen. Data from the crystals were collected at the European Synchrotron Radiation Facility (ESRF) using the ID30B beamline. Data were processed using autoPROC and STARANISO. The structure was solved by molecular replacement using coordinates of PNGaseA^Bif^ model generated using Alpha-fold colab^57^. The structures were phased and refined using Phenix^58^. Manual building was carried out in COOT^59^. The structure was analysed and figures generated using Pymol^60^ and ChimeraX^61^. Consurf was used to assess conservation of residues over the PNGaseA^Bif^ structure^62^. Despite soaking with chitobiose, no ligand was present in the active site.

For the mutant structures, PNGaseA^Bif^ E236Q and PNGaseA^Bif^ D107N–E236Q were purified as described above. PNGaseA^Bif^ E236Q was concentrated to 15 mg/mL and crystals were obtained in 30% (w/v) PEG 8000 and 200 mM Ammonium Sulfate (Jena Bioscience). PNGaseA^Bif^ D107N–E236Q was concentrated to 15 mg/mL and co-crystallised with 2.5 mM G2-peptide (TCI Chemicals). Crystals were obtained in 30% (w/v) PEG 4000, 100 mM Tris-HCl, pH 8.5, 200 mM sodium acetate and 1 mM Anderson–Evans polyoxotungstate (Jena Bioscience). Crystals were cryoprotected in mother liquor supplemented with 20% glycerol and flash-cooled in liquid nitrogen. Diffraction data were collected on beamline BL13-XALOC at ALBA at a wavelength of 0.97 Å and a temperature of 100 K. Data were processed and scaled using XDS^63^ and CCP4^64^. Relevant statistics are given in Supplementary Table 2. The structure was solved by molecular replacement with Phaser^65^ using an AlphaFold model as search model^57^. Iterative cycles of manual rebuilding in COOT^59^. and refinement in REFMAC5^66^ were used to obtain the final model, which was validated with PROCHECK. Model statistics are summarised in Supplementary Table 2. The Ramachandran analysis of the final models gave 96.83%/2.89%/0.28% (favoured/allowed/outliers) for wild-type PNGaseA^Bif^, 95.3%/3.5%/1.1% for PNGaseA^Bif^ E236Q, and 90.3%/9.7%/0.0% for the PNGaseA^Bif^ D107N–E236Q–G2-peptide complex.

### Modelling in of short peptides

Enzyme-substrate complex structures were predicted using the AlphaFold 3 server. Amino acid sequences corresponding to the PNGaseABif sequence and either AANASAAAA, ANASAAAA, AAANASAA or AAANASAA were input for modelling. Predictions were generated using the default AlphaFold 3 server settings. Multiple predicted models were generated for each enzyme-substrate pair, and the resulting structures were assessed using the confidence metrics provided by AlphaFold 3, including predicted aligned error (PAE) and the pLDDT scores (Supplementary Fig. 26). The model with the highest metrics was selected for visualisation in ChimeraX.

### Molecular dynamics simulations

The starting point for molecular dynamics (MD) simulations conducted in this study was the crystal structure of PNGaseA^Bif^ D107N–E236Q in complex with G2-peptide (PDB ID 29JQ). Catalytic residues mutations (D107N and E236Q) were reverted using PyMOL. Protonation states of these residues were chosen using Protoss server^33^. The carboxylic group of E236 was considered to be protonated to keep important interactions with G2-peptide. The system was inserted in an orthorhombic box with a minimum distance of 15 $\mathring{\mathrm{A}}$ between the solute and the edges (box dimensions 112.496 × 87.922 × 97.185 ${\mathring{\mathrm{A}} }^{3}$). The box was solvated with TIP3P water molecules^67^ and sodium and chloride ions were added to neutralize the charge, resulting in a total of 80716 atoms of which 24169 are water molecules. Protein residues were described using Amber ff14SB force field^68^, while carbohydrates were described using GLYCAM06^69^. Both force fields have been demonstrated to accurately reproduce the structural and dynamic properties of carbohydrate acting enzymes in molecular dynamics simulations^32,70^. LEaP module of AmberTools^71^ was used to obtain topology and coordinate files for the MD simulations. Simulations were performed using Amber22^72^ with the CUDA version of the PMEMD module^73^. The solvated system was minimized in two steps: first the solvent was minimized with 5000 steps of steepest descent minimization and 5000 steps of conjugent gradient, with positional restraints on all atoms of the PNGaseA^Bif^ and the G2-peptide; and secondly, the entire system was minimized with 7500 steps of steepest descent and 7500 steps of conjugant gradient. Then, the system was heated to 300 K in the NVT ensemble using the Berendsen thermostat, with a time constant coupling of 1.0 ps for the coupling and 50 kcal mol^−1^ Å^−^^2^ positional restraints applied to heavy atoms of PNGaseA^BIf^ and the G2-peptide. Restraints were decreased to 5 kcal mol^-1^ Å^−^^2^ gradually. After that, the systems were equilibrated during 1000 ps in the NPT ensemble using the Berendsen thermostat and barostat to maintain the system at 300 K and 1 atm. Three replicas of 500 ns were run with a time step of 2 fs in the NPT ensemble with the Langevin thermostat and the Berendsen barostat. The SHAKE algorithm^74^ was used to constrain all bonds that involve hydrogen atoms. Molecular dynamics simulations for the apo structure of PNGaseA^Bif^ were performed following the same protocol after removal of the G2-peptide from PDB ID 29JQ. Trajectory analysis was performed using CPPTRAJ^75^.

### 3D structure modelling

Three-dimensional structures of wild-type PNGaseA^Bif^ in complex with core α1,6-fucosylated G2-peptide and core α1,3-fucosylated G2-peptide were predicted using Protenix^76^ web server (https://protenix-server.com/add-prediction).

### Glycan labelling

Released N-glycans were fluorescently labelled by reductive amination procainamide as using LudgerTagTM Procainamide Glycan Labelling Kit (LT-KPROC-24)^77^. Briefly, samples in 10 μL of pure water were incubated for 60 min at 65 °C with procainamide labelling solution. The procainamide labelled samples were cleaned-up using a LudgerClean procainamide plate (LC-PROC-96). The purified procainamide labelled N-glycans were eluted with pure water (200 μL). The samples were dried by vacuum centrifugation and resuspended in pure water (150 μL) for further analysis.

### LC-FLD-ESI-MS

Procainamide labelled samples were analysed by LC-FLD-ESI-MS using an ACQUITY UPLC® BEH-Glycan 1.7 µm, 2.1 × 150 mm column at 40 °C on a Thermo Scientific UltiMate 3000 UPLC instrument with a fluorescence detector (λex = 310 nm, λem = 370) controlled by HyStar software version 3.2. Gradient conditions were: 0 to 53.5 min, 24 to 49 % A at a flow rate of 0.4 mL/min; 53.5 to 55.5 min, 49 to 100% A, 0.4 to 0.2 mL/ml; 55.5 to 57.5 min, 100% A at 0.2 mL/min; 57.5–59.5 min, 100 to 24% A at 0.2 mL/min; 59.5 to 65.5 min, 24% A at 0.2 mL/min; 65.5 to 66.5 min, 24% A from 0.2 to 0.4 mL/min; 66.5 to 70 min 24% A at 0.4 mL/min. Solvent A was 50 mM ammonium formate pH 4.4 made from Ludger Stock Buffer (LS-N-BUFFX40); Solvent B was acetonitrile (Acetonitrile 190 far UV/gradient quality; Romil #H049). Samples were injected in 24% aqueous/76% acetonitrile; injection volume 20 µL. The UPLC system was coupled on-line to an AmaZon Speed ETD electrospray mass spectrometer (Bruker Daltonics, Bremen, Germany) with the following settings: source temperature 180 °C, gas flow 10 L/min; Capillary voltage 4500 V; ICC target 100,000; maximum accumulation time 50 ms; rolling average 5; number of precursors ions selected 3, release after 0.2 min; Positive ion mode; Scan mode: enhanced resolution; mass range scanned, 400–1700. A glucose homopolymer ladder labelled with procainamide (Ludger; CPROC-GHP-30) was used as a system suitability standard as well as an external calibration standard for GU allocation. ESI-MS and MS/MS data analysis was performed using Bruker Compass DataAnalysis V4.1 software and GlycoWorkbench software^78^.

### AlphaFold models of PNGaseA family members

Models were downloaded from https://www.ebi.ac.uk/interpro and figures made directly from that information. Models were generated with the full sequence (See Source Data and Supplementary Figs. 27 and 28).

### Statistics and reproducibility

No statistical method was used to predetermine sample size. No data were excluded from the analyses. The experiments were not randomized. The investigators were not blinded to allocation during experiments and outcome assessment.

### Reporting summary

Further information on research design is available in the Nature Portfolio Reporting Summary linked to this article.

## Supplementary information

Supplementary Information

Supplementary Dataset 1

Supplementary Dataset 2

Reporting Summary

Transparent Peer Review file

## Source data

Source Data

## Data Availability

The atomic coordinates and structure factors have been deposited in the Protein Data Bank (ID code 9RZW (apo structure), 29JO (E236Q mutant structure) and 29JQ (D107N–E236Q mutant-G2-peptide complex). The corresponding PDB DOIs are https://doi.org/10.2210/pdb9RZW/pdb, https://doi.org/10.2210/pdb29JO/pdb, and https://doi.org/10.2210/pdb29JQ/pdb. Raw data for the molecular dynamic simulations can be found in 10.5281/zenodo.20627294. Protenix models can be found in Supplementary Data items. Source data are provided with this paper.
