# Supplementary material for: Structural basis for N-glycopeptide preference in an infant-derived Bifidobacterium bifidum PNGaseA
Source: Nat Commun. 2026 Nov 2;17:11119. doi: 10.1038/s41467-026-78972-1 (PMC13728245; doi:10.1038/s41467-026-78972-1)
Supplement: Supplementary file 1 [file 41467_2026_78972_MOESM1_ESM.pdf]

Supplementary information

Structural basis for N-glycopeptide preference in an infant-derived *Bifidobacterium bifidum* PNGaseA

Cassie R. Bakshani[1], Simon G. Caulton[2], Victor Taleb[3], Taiwo O. Ojuri[1], Keith Coughlan[4,5], Alejandro Montesa[3,6], Alejandro Pérez-Latorre[3], Pedro Merino[3,6], Radoslaw P. Kozak[7], Sara Çakaj[1], Francesca Bottacini[4,5], Andrew L. Lovering[2], Douwe van Sinderen[4], Ramón Hurtado-Guerrero[3,8,9]✉ ,and Lucy I. Crouch[1]✉

1. Department of Microbes, Infection and Microbiomes, School of Infection, Inflammation and Immunology, College of Medicine and Health, Institute of Microbiology and Infection, University of Birmingham, Birmingham, B15 2TT, UK.
2. School of Biosciences, University of Birmingham, Birmingham, B15 2TT, UK.
3. Institute of Biocomputation and Physics of Complex Systems (BIFI), University of Zaragoza, Mariano Esquillor s/n, Campus Rio Ebro, Edificio I+D, Zaragoza, Spain.
4. APC Microbiome Ireland and School of Microbiology, University College Cork, Western Road, Cork, Ireland.
5. Department of Biological Sciences, Munster Technological University Cork, Ireland.
6. Department of Organic Chemistry. Faculty of Sciences, University of Zaragoza, Campus San Francisco, 50009, Zaragoza, Spain
7. Ludger Ltd, Culham Campus, OX14 3EB, Abingdon, UK.
8. Copenhagen Center for Glycomics/Glycocalyx, Department of Cellular and Molecular Medicine, University of Copenhagen, Copenhagen, Denmark.
9. Fundación ARAID, Zaragoza, Spain.

✉Address correspondence to l.i.crouch@bham.ac.uk and rhurtado@bifi.es

a

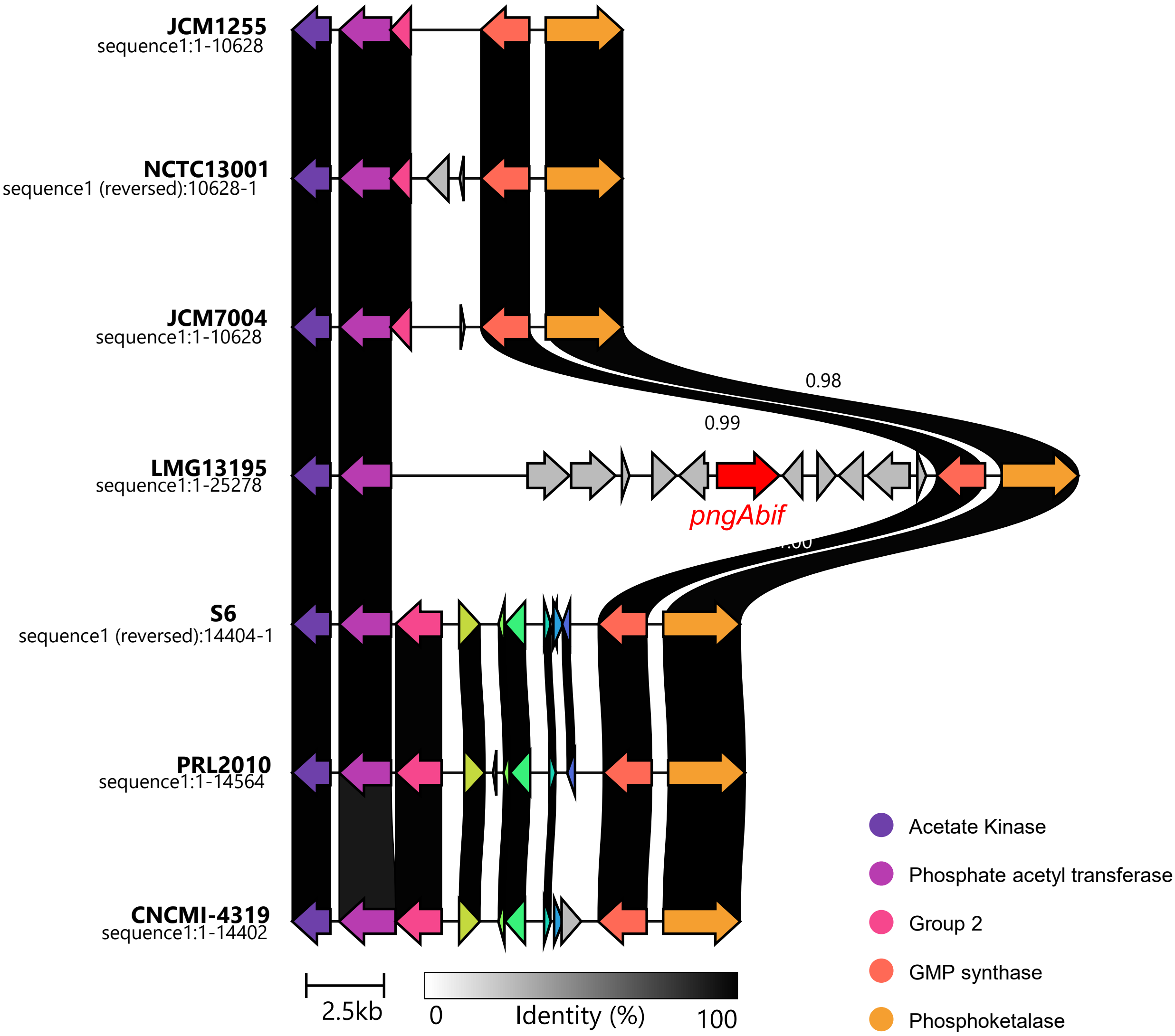

Supplementary Figure 1. Genetic context of *pngAbif.* The gene cluster where *pngAbif* is found (red) in LMG13195 is shown in comparison to other *B. bifidum* strains. Out of the eighteen complete genomes, eight different proposed gene clusters could be identified. The conservation of the different genes is displayed through the greyscale mapping between the genes, which indicates near 100 % identity between all genes assesses. The flanking genes for these gene clusters consist of acetate kinase and a phosphate acetyl transferase (purples), typically on the left flank, as well as a GMP synthase and phosphoketalase (oranges), typically on the right flank. Inversions of this gene cluster are possible, and one such inverted cluster can be observed in the PRI 1 gene cluster, which was re-orientated for purpose of illustration here.

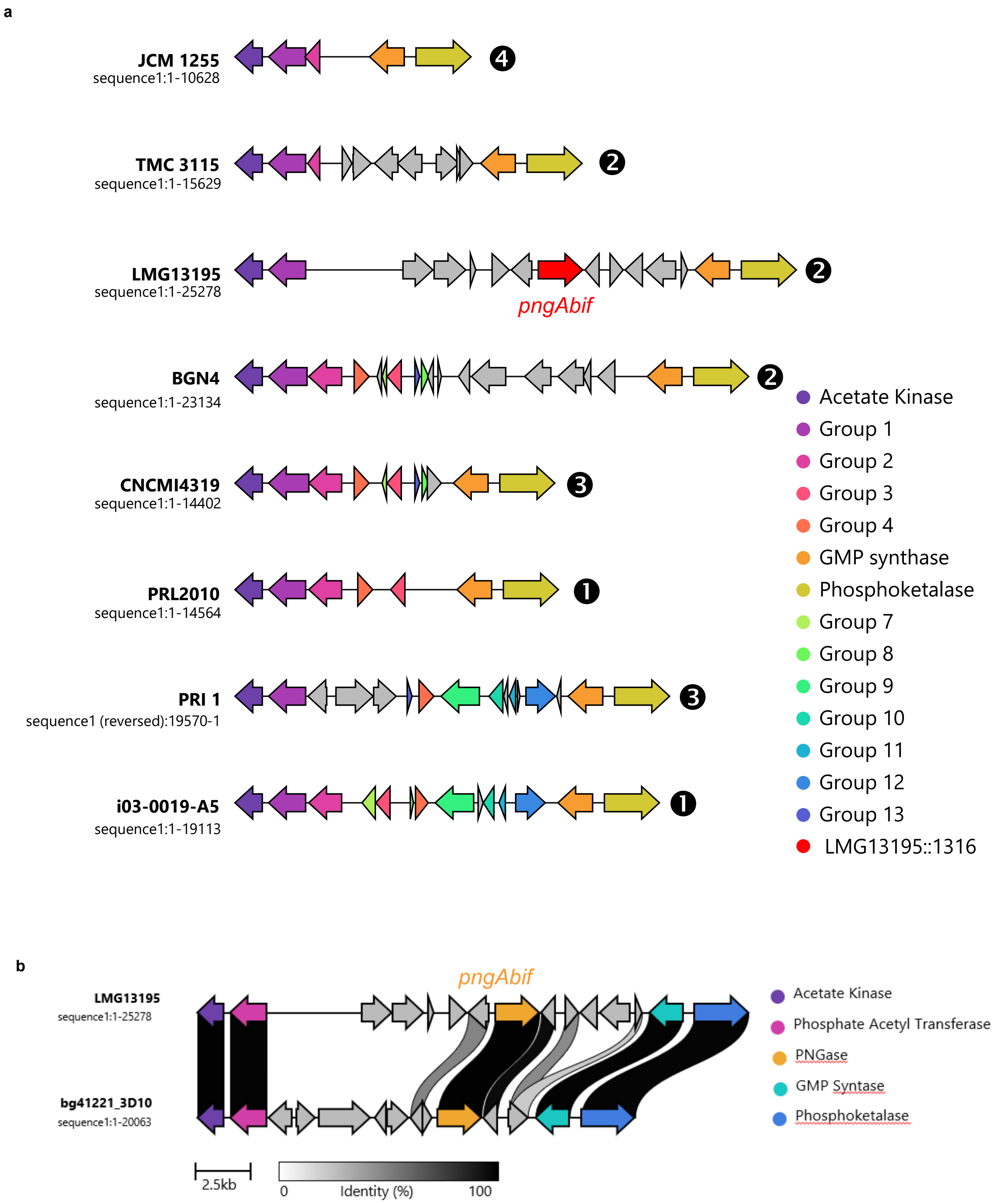

Supplementary Figure 2. Variable region in *Bifidobacterium bifidum* genomes. a) Out of the eighteen complete genomes, eight different proposed gene clusters could be identified. The *pngAbif* is highlighted in red and homologues are indicated by matching colours. The number of times a particular genome structure was observed is indicated by the white numbers in black circles. b) A comparison of the two *B. bifidum* genomes encoding genes for putative PNGaseA enzymes. There is some variation between the two, but *pngAbif* is highly conserved.

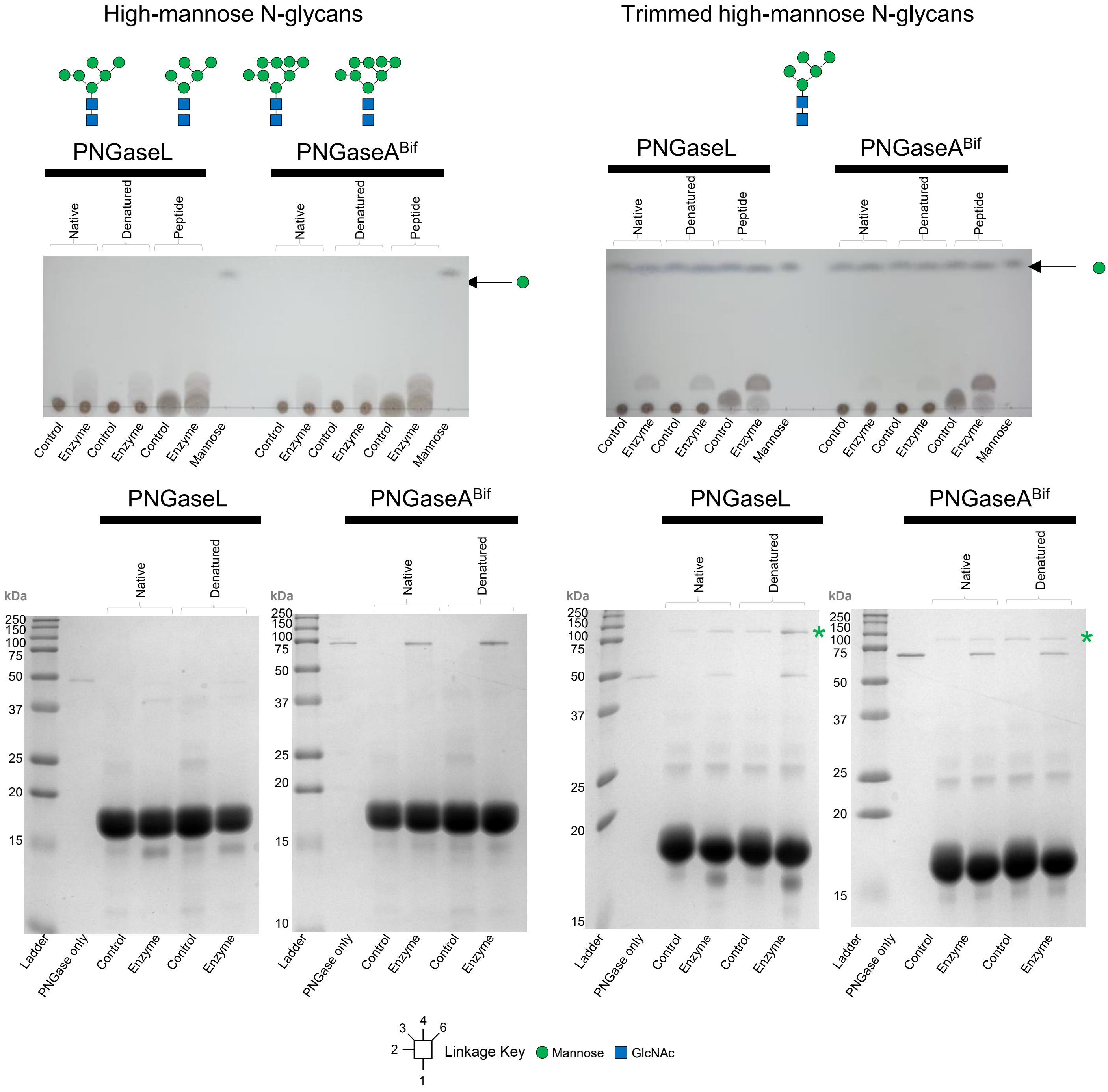

Supplementary Figure 3. Biochemical Characterisation of PNGaseA^Bif against high-mannose N-glycans. Bovine RNaseB was used to assess the specificity of PNGaseA^Bif (left) for high-mannose N-glycans and PNGaseL was used as a positive control (right). The peptide was provided to the PNGase enzymes in three forms – native, denatured (boiling), and peptide (*Vibrio coralliilyticus* treated). The impact of removing the α1,2-linked mannose from the non-reducing ends of the glycans was also investigated using BT3990 from *Bacteroides thetaiotaomicron*. The N-glycan release was assessed through thin layer chromatography (top) and change in glycoprotein size was monitored through SDS-PAGE with staining for protein (middle) PNGaseL and PNGaseA^Bif have molecular weights of 50.6 and 60.9 kDa, respectively. BT3990 has a molecular weight of 86 kDa (green asterisk).

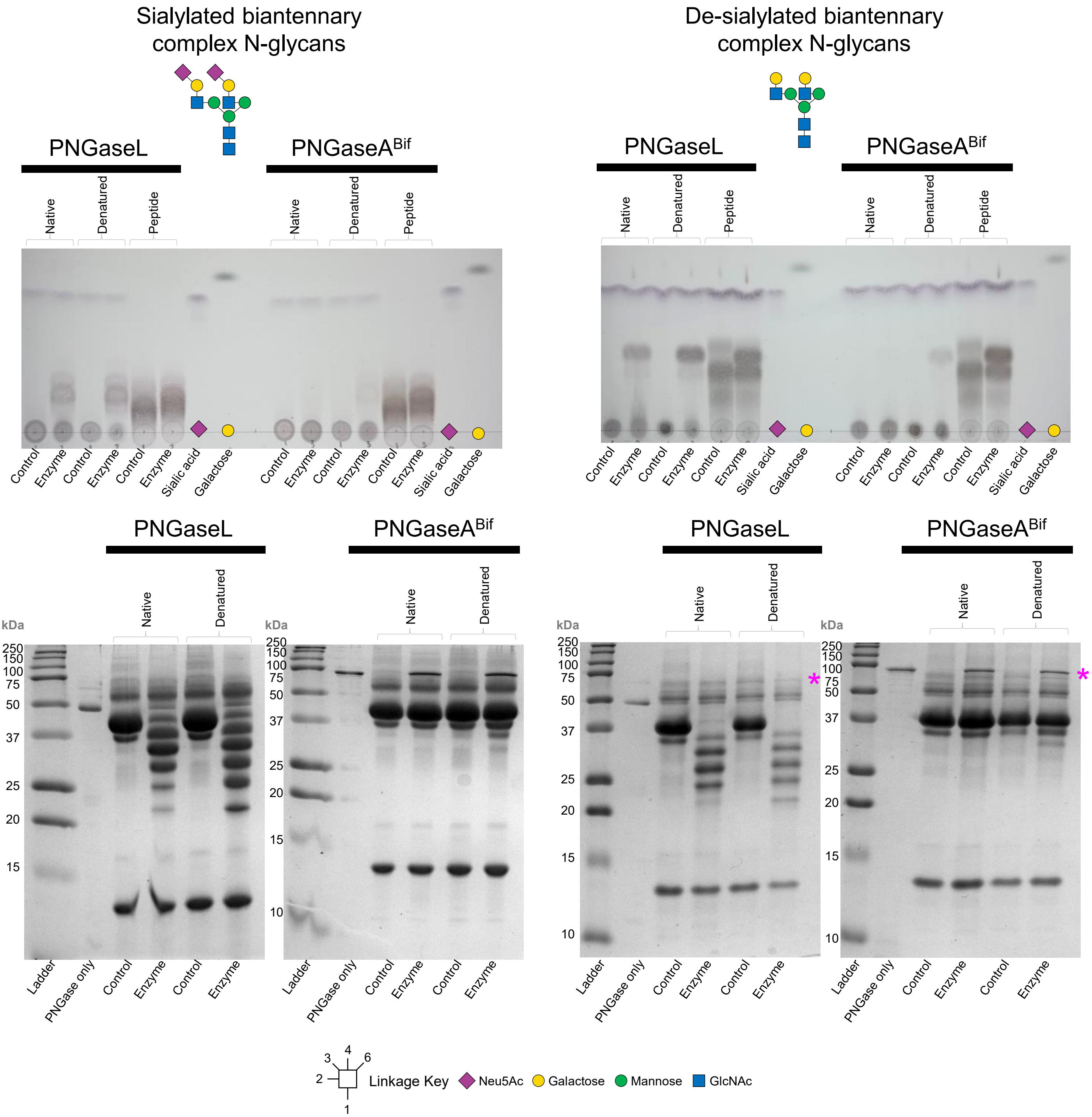

Supplementary Figure 4. Biochemical Characterisation of PNGaseA^{Bif} against mammalian biantennary complex N-glycans. Bovine α1acid glycoprotein was used to assess the specificity of PNGaseA^{Bif} for mammalian biantennary complex N-glycans and PNGaseL was used as a positive control. The protein component of the substrate was provided to the PNGase enzymes in three forms – native, denatured (boiling), and peptide (*Vibrio coralliilyticus* treated). The impact of removing the α-linked sialic acid from the non-reducing ends of the glycans was also investigated using BT0455 from *Bacteroides thetaiotaomicron* (right). The N-glycan release was assessed through thin layer chromatography (top) and change in glycoprotein size was monitored through SDS-PAGE with staining for protein (bottom). PNGaseL and PNGaseA^{Bif} have molecular weights of 50.6 and 60.9 kDa, respectively. BT0455 has a molecular weight of 6.6 kDa (fuchsia asterisk). Assays were carried out for three biological replicates.

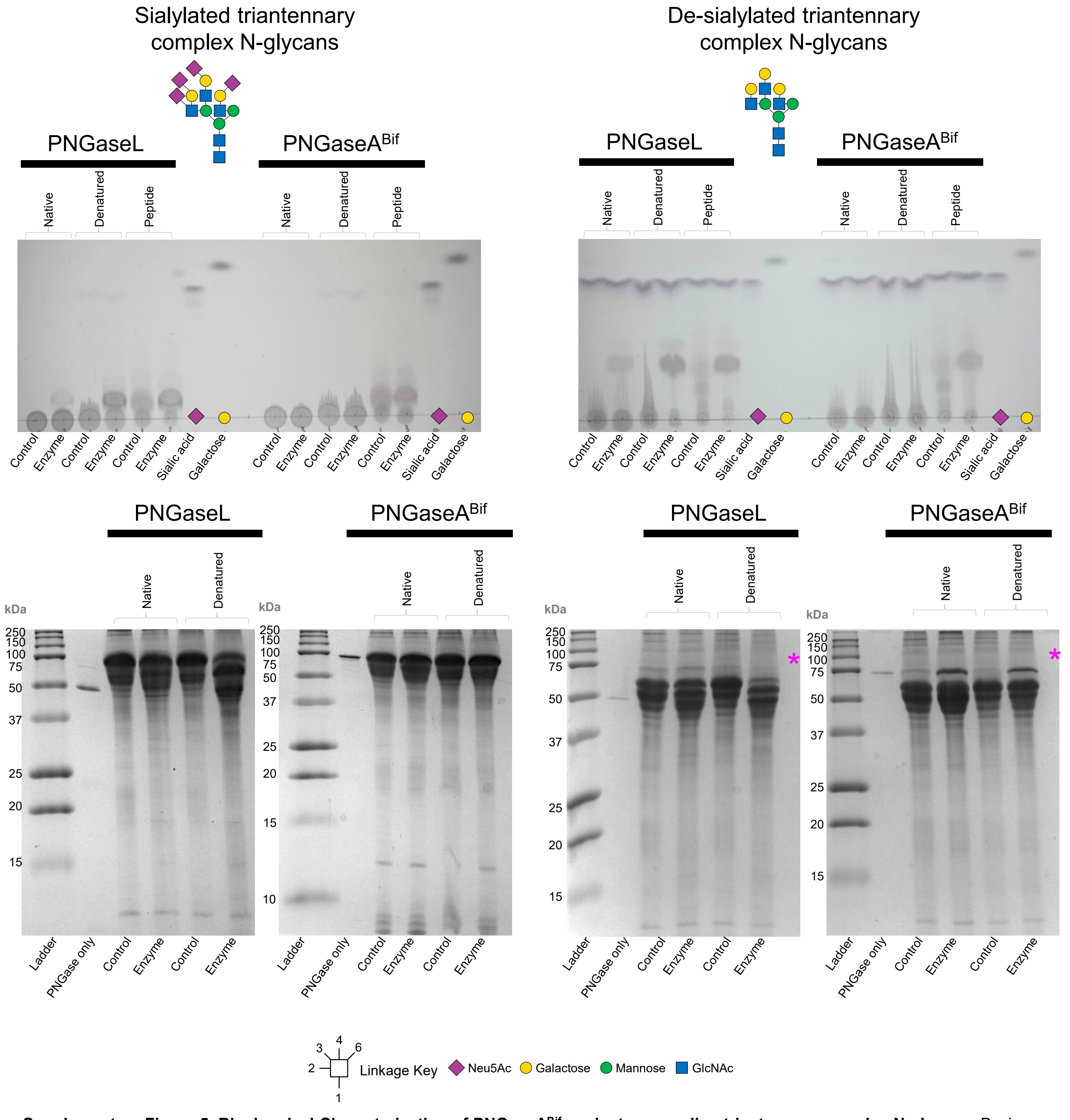

Supplementary Figure 5. Biochemical Characterisation of PNGaseA^{Bif} against mammalian triantennary complex N-glycans. Bovine fetuin was used to assess the specificity of PNGaseA^{Bif} for mammalian triantennary complex N-glycans and PNGaseL was used as a positive control. The protein component of the substrate was provided to the PNGase enzymes in three forms – native, denatured (boiling), and peptide (*Vibrio coralliilyticus* treated). The impact of removing the α-linked sialic acid from the non-reducing ends of the glycans was also investigated using BT0455 from *Bacteroides thetaiotaomicron* (right). The N-glycan release was assessed through thin layer chromatography (top) and change in glycoprotein size was monitored through SDS-PAGE with staining for protein (bottom). PNGaseL and PNGaseA^{Bif} have molecular weights of 50.6 and 60.9 kDa, respectively. BT0455 has a molecular weight of 6.6 kDa (fuchsia asterisk). Assays were carried out for three biological replicates.

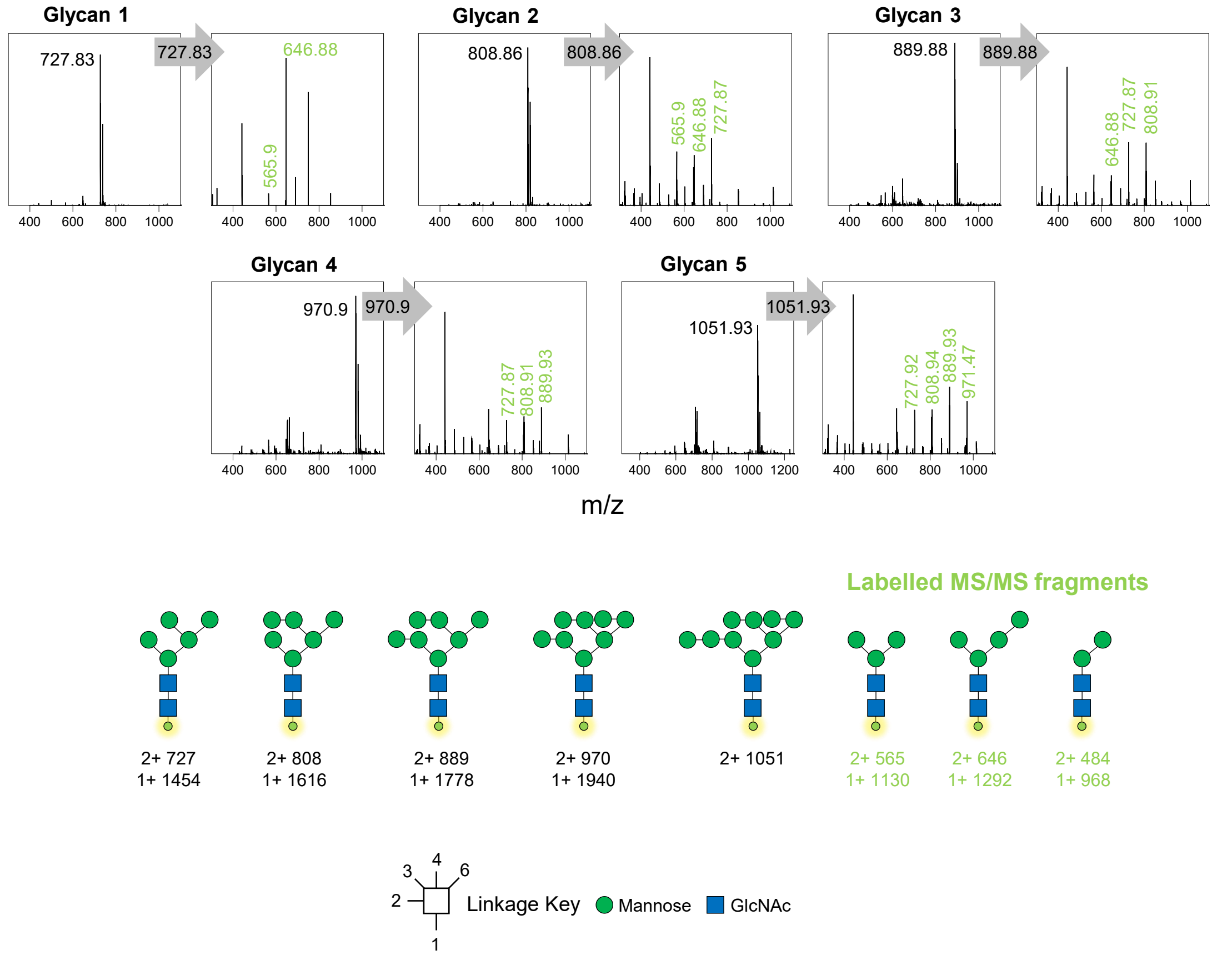

Supplementary Figure 6. Mass spectrometry data for the high-mannose N-glycans. Examples of mass spectra for each glycan from the assays in Figure 2. Mass spec of the different glycans (black) and the accompanying MS/MS data is shown to the right. The grey arrows connecting the panels indicate which MS peak the MS/MS data originates from. The green labelling indicates the masses corresponding to fragmentation ions with procainamide labelling. Source data are provided in the Source Data file.

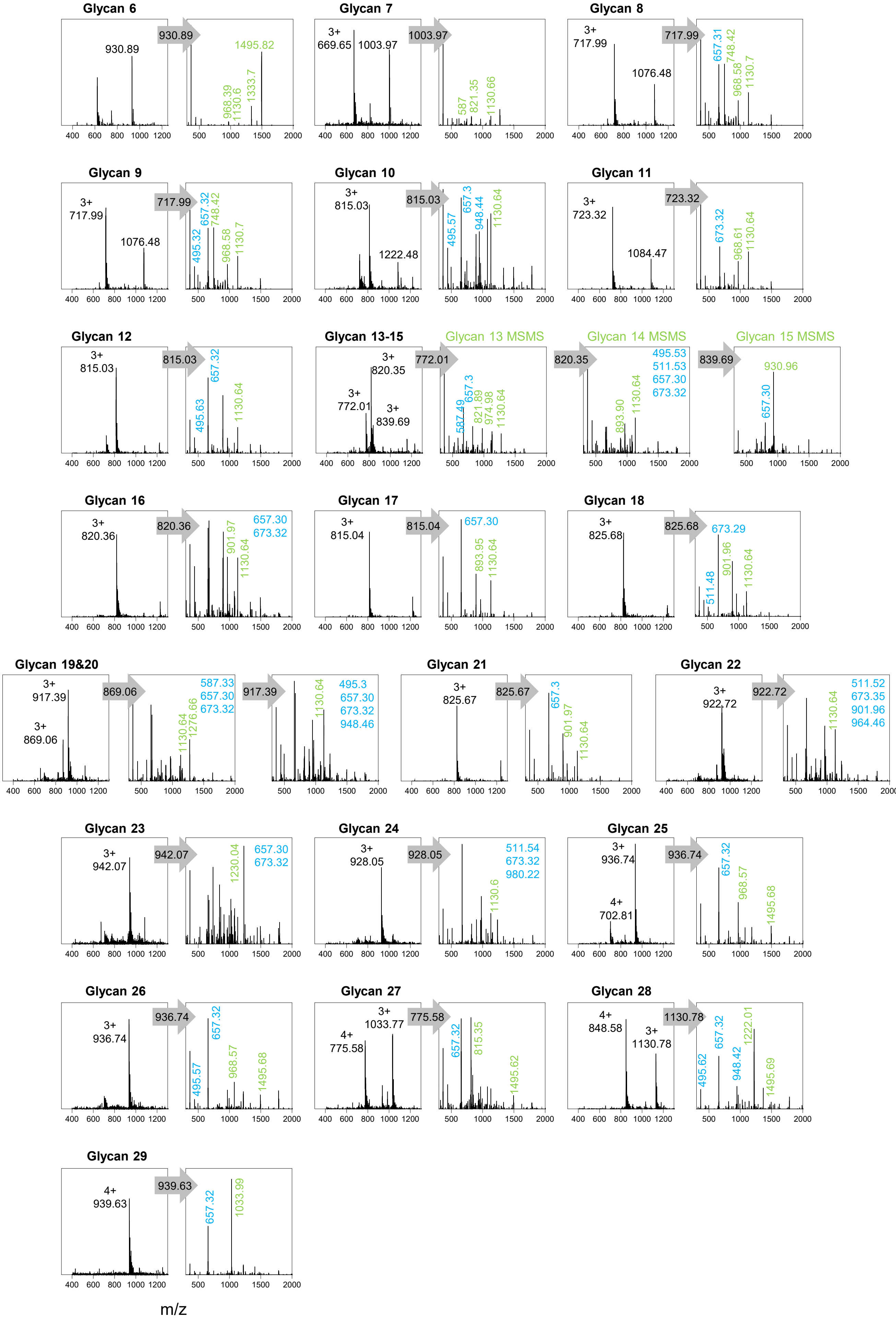

Glycan 6
930.89
930.89
1495.82
968.39
1130.6
1333.7
Glycan 7
3+
669.65
1003.97
1003.97
587
821.35
1130.66
Glycan 8
3+
717.99
1076.48
717.99
657.31
748.42
968.58
1130.7
Glycan 9
3+
717.99
1076.48
717.99
495.32
657.32
748.42
968.58
1130.7
Glycan 10
3+
815.03
1222.48
815.03
495.57
657.3
948.44
1130.64
Glycan 11
3+
723.32
1084.47
723.32
673.32
968.61
1130.64
Glycan 12
3+
815.03
815.03
495.63
657.32
1130.64
Glycan 13-15
3+
820.35
3+
772.01
3+
839.69
Glycan 13 MSMS
772.01
587.49
657.3
821.89
974.98
1130.64
Glycan 14 MSMS
820.35
495.53
511.53
657.30
673.32
893.90
1130.64
Glycan 15 MSMS
839.69
930.96
657.30
Glycan 16
3+
820.36
820.36
657.30
673.32
901.97
1130.64
Glycan 17
3+
815.04
815.04
657.30
893.95
1130.64
Glycan 18
3+
825.68
825.68
673.29
511.48
901.96
1130.64
Glycan 19&20
3+
917.39
3+
869.06
869.06
587.33
657.30
673.32
1130.64
1276.66
917.39
1130.64
495.3
657.30
673.32
948.46
Glycan 21
3+
825.67
825.67
657.3
901.97
1130.64
Glycan 22
3+
922.72
922.72
1130.64
511.52
673.35
901.96
964.46
Glycan 23
3+
942.07
942.07
1230.04
657.30
673.32
Glycan 24
3+
928.05
928.05
511.54
673.32
980.22
1130.6
Glycan 25
3+
936.74
4+
702.81
936.74
657.32
968.57
1495.68
Glycan 26
3+
936.74
936.74
495.57
657.32
968.57
1495.68
Glycan 27
3+
1033.77
4+
775.58
775.58
657.32
815.35
1495.62
Glycan 28
4+
848.58
3+
1130.78
1130.78
495.62
657.32
948.42
1222.01
1495.69
Glycan 29
4+
939.63
939.63
657.32
1033.99
400 600 800 1000 1200
500 1000 1500 2000
m/z

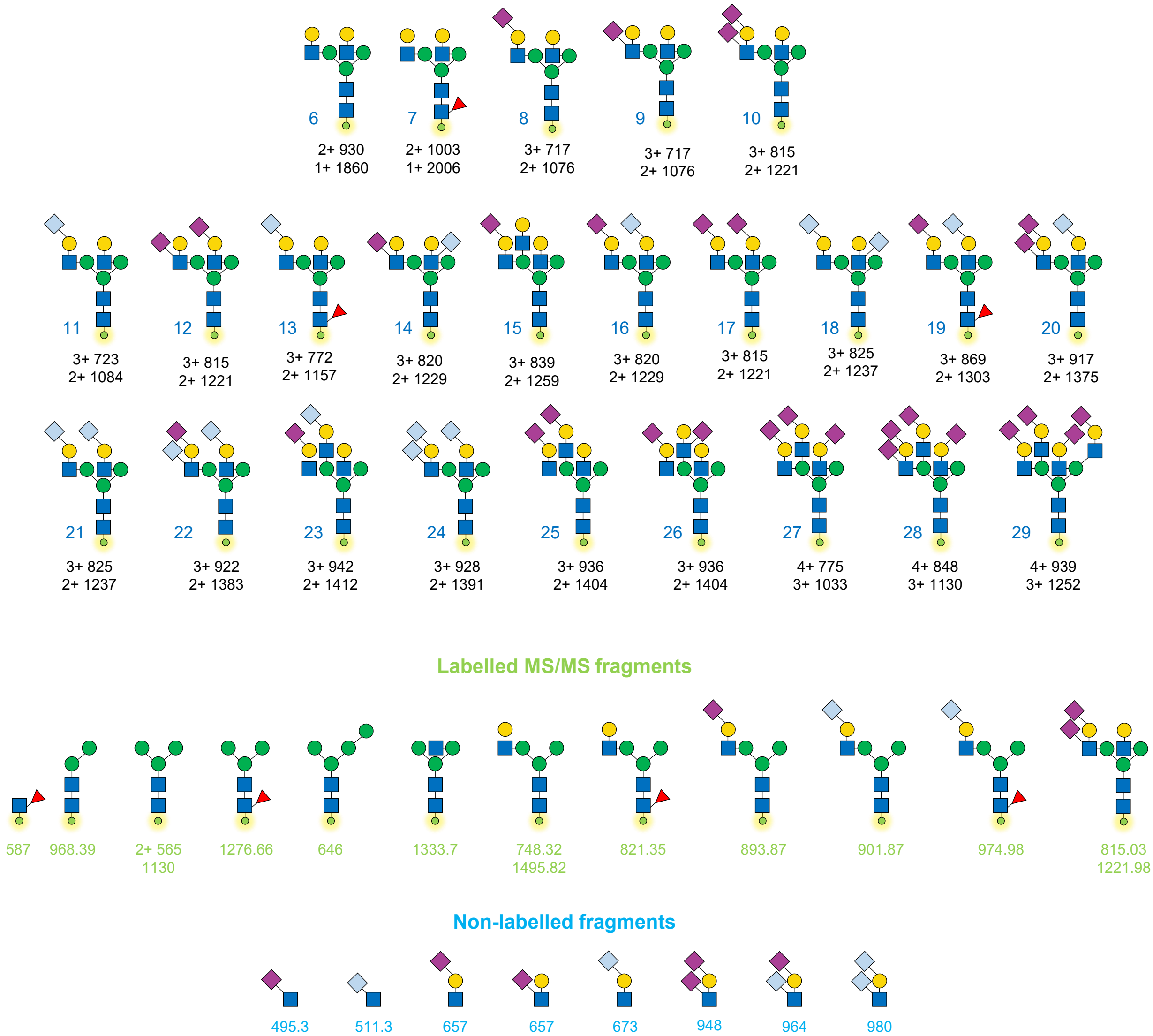

Supplementary Figure 7. Mass spectrometry data for the complex N-glycans. Examples of mass spectra for each glycan from the assays in Figure 2. Mass spec of the different glycans (black) and the accompanying MS/MS data is shown to the right (some examples have multiple glycans detected in one spectra). The grey arrows connecting the panels indicate which MS peak the MS/MS data originates from. The green labelling indicates the masses corresponding to fragmentation ions with procainamide labelling and the green labelling indicates the masses corresponding to fragmentation ions with no labelling. Source data are provided in the Source Data file.

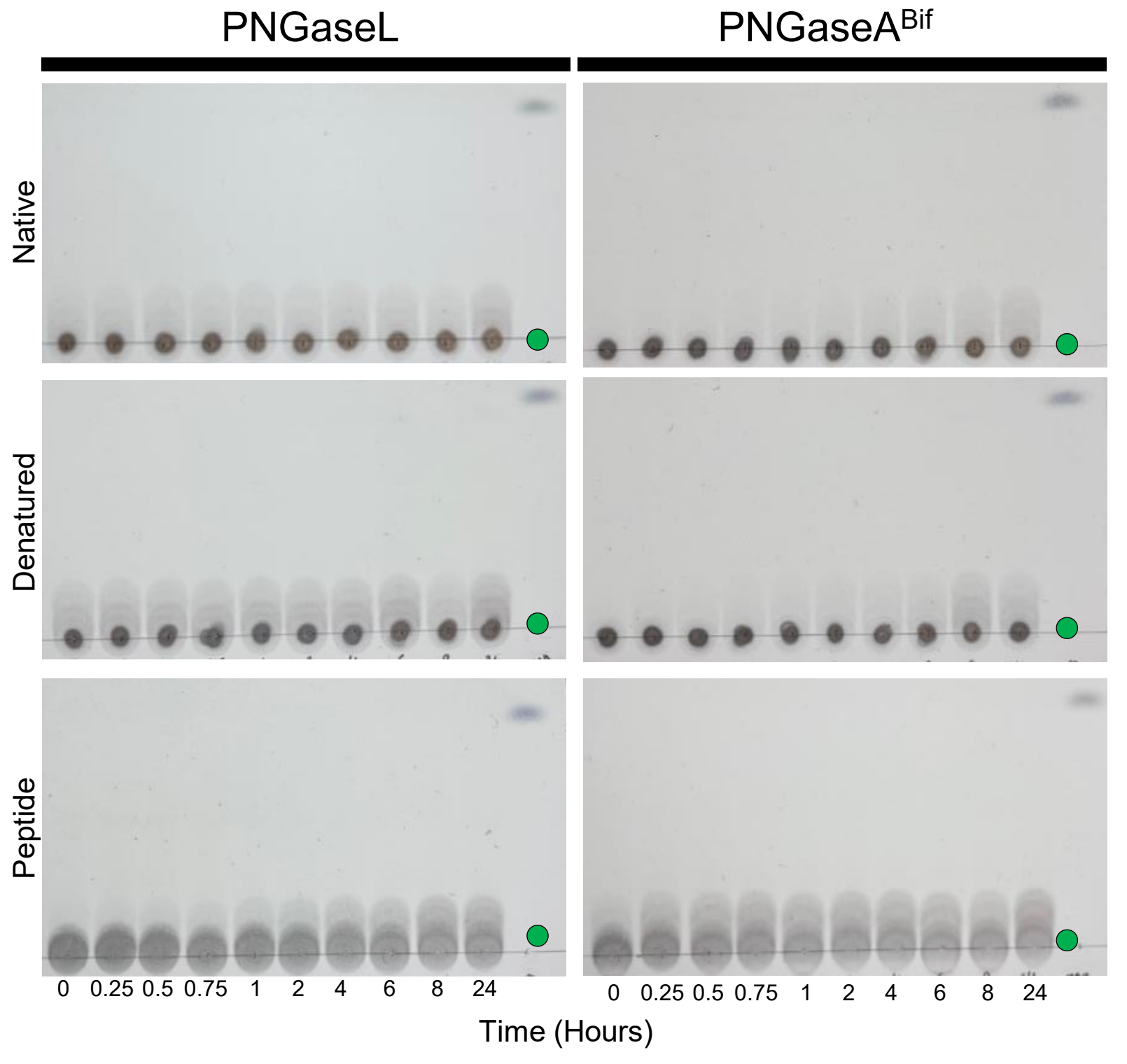

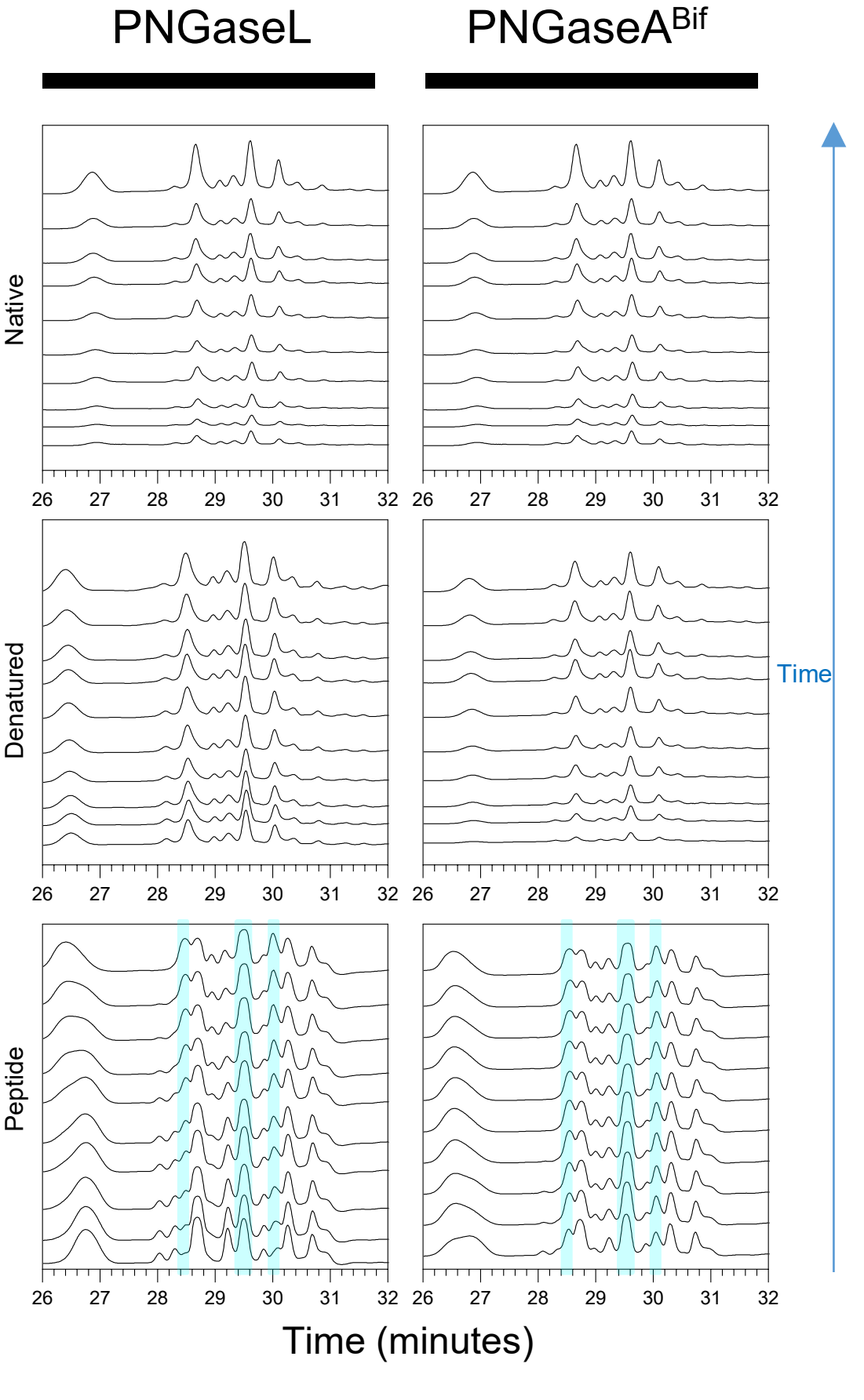

Supplementary Figure 8. Time course assays – high-mannose N-glycan. The release of high-mannose N-glycan from bovine RNaseB by PNGaseL and PNGaseA^{Bif} was monitored over time and compared three different conditions. Final concentration of enzymes was 1 µM. The results were visualised by TLC and then HPAEC-PAD. For the HPAEC-PAD, the released N-glycans for the peptide substrate are highlighted in blue. Source data are provided in the Source Data file. This experiment was carried out once.

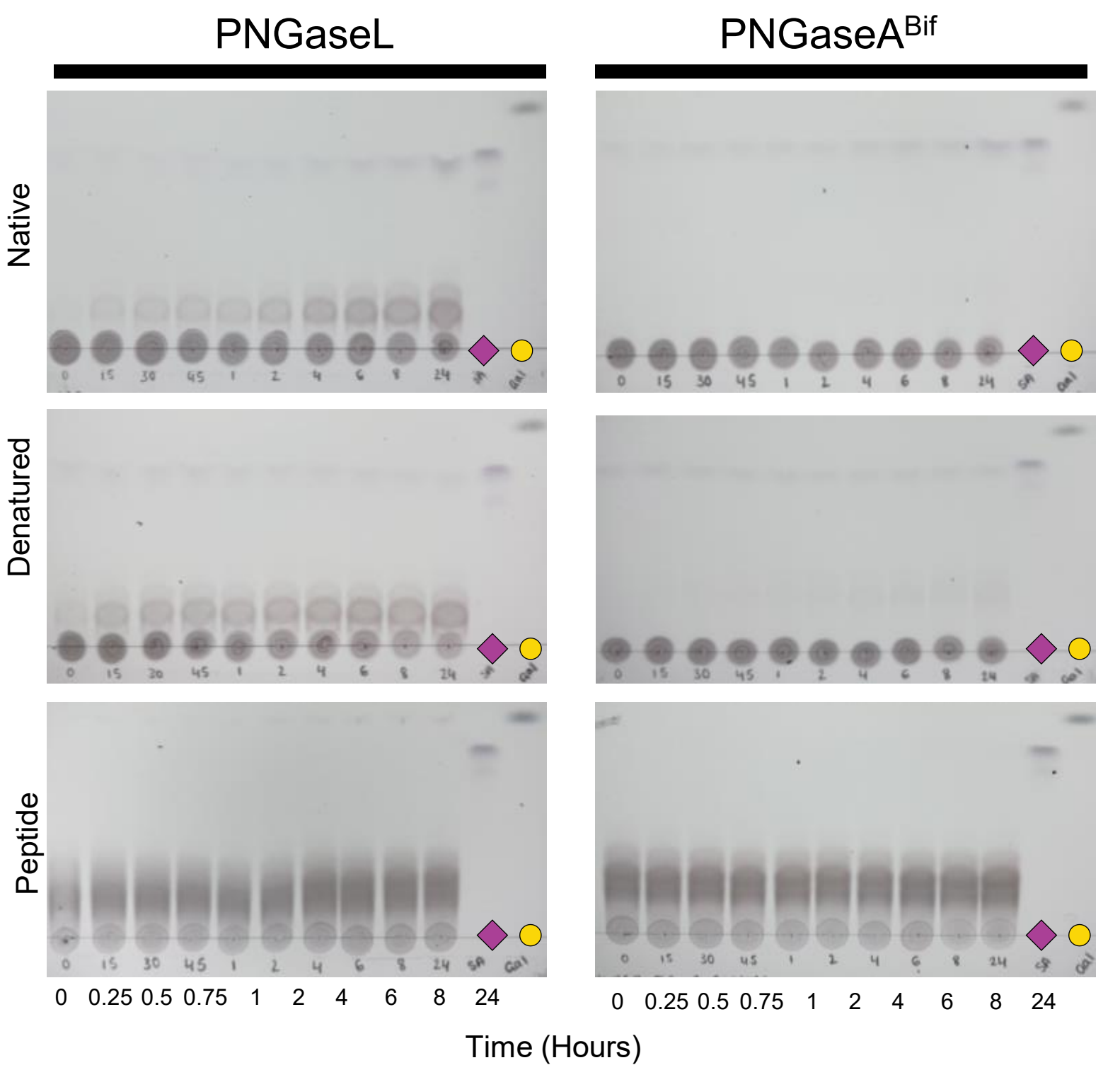

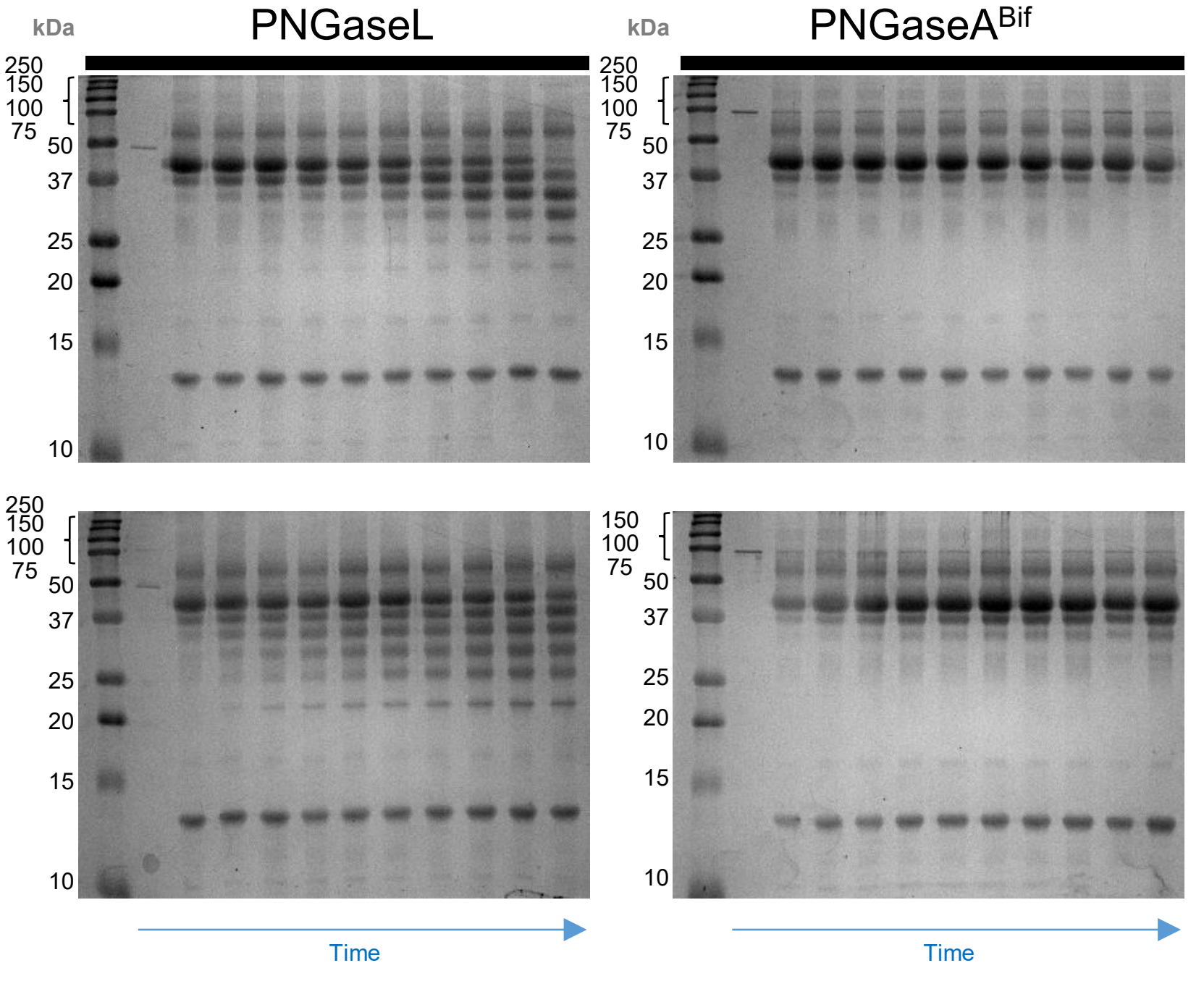

Supplementary Figure 9. Time course assays – mammalian biantennary complex N-glycan 1. The release of high-mannose N-glycan from bovine RNaseB by PNGaseL and PNGaseA^{Bif} was monitored over time and compared three different conditions. Concentration of enzymes was 1 μM. The results were visualised by TLC (top) and SDS-PAGE (bottom). This experiment was carried out once.

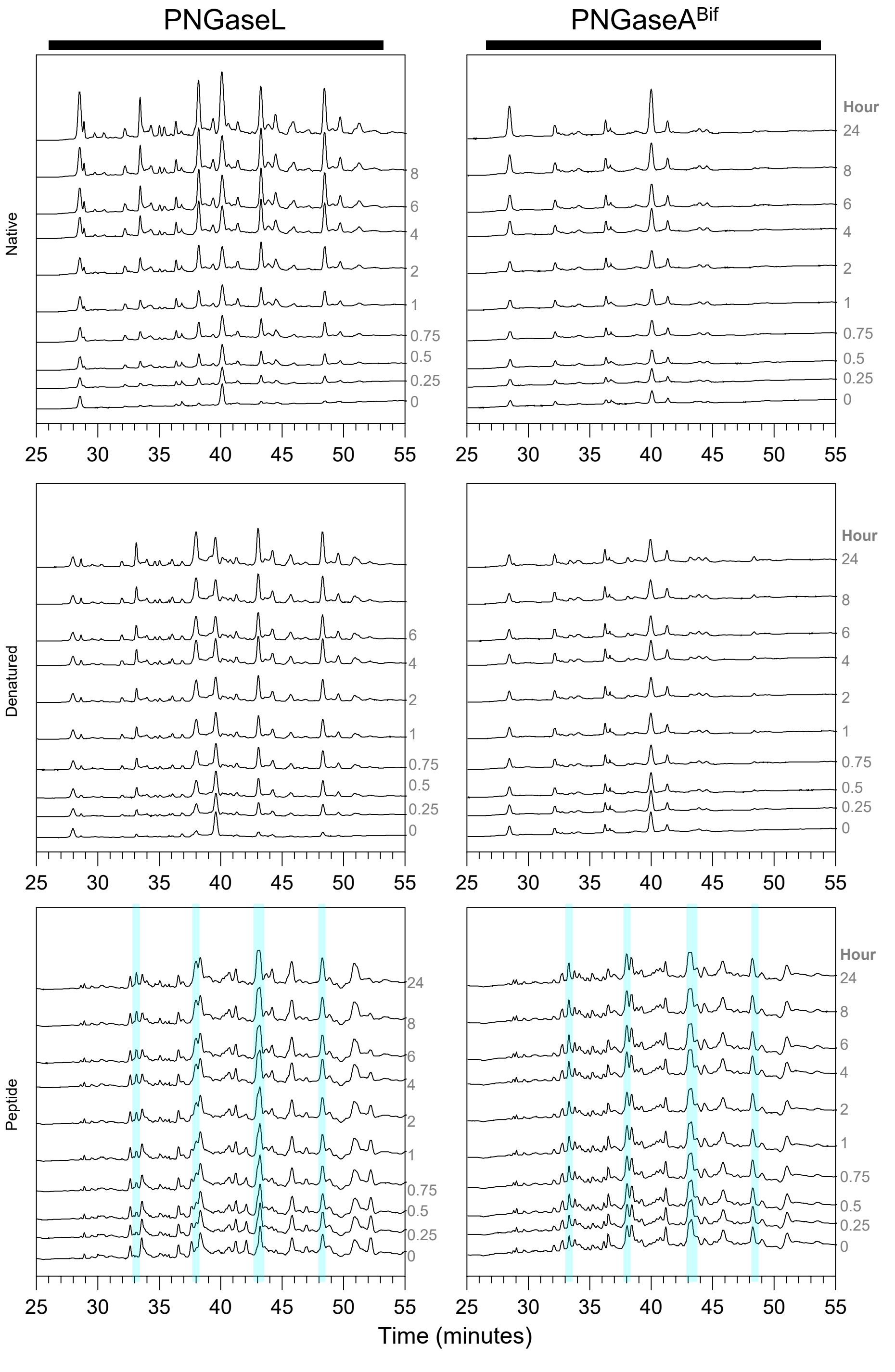

Supplementary Figure 10. Time course assays – mammalian biantennary complex N-glycan 2. The release of high-mannose N-glycan from bovine RNaseB by PNGaseL and PNGaseA^{Bif} was monitored over time and compared three different conditions. Concentration of enzymes was 1 μM. The results were visualised by HPAEC-PAD. The released N-glycans for the peptide substrate are highlighted in blue. Source data are provided in the Source Data file. This experiment was carried out once.

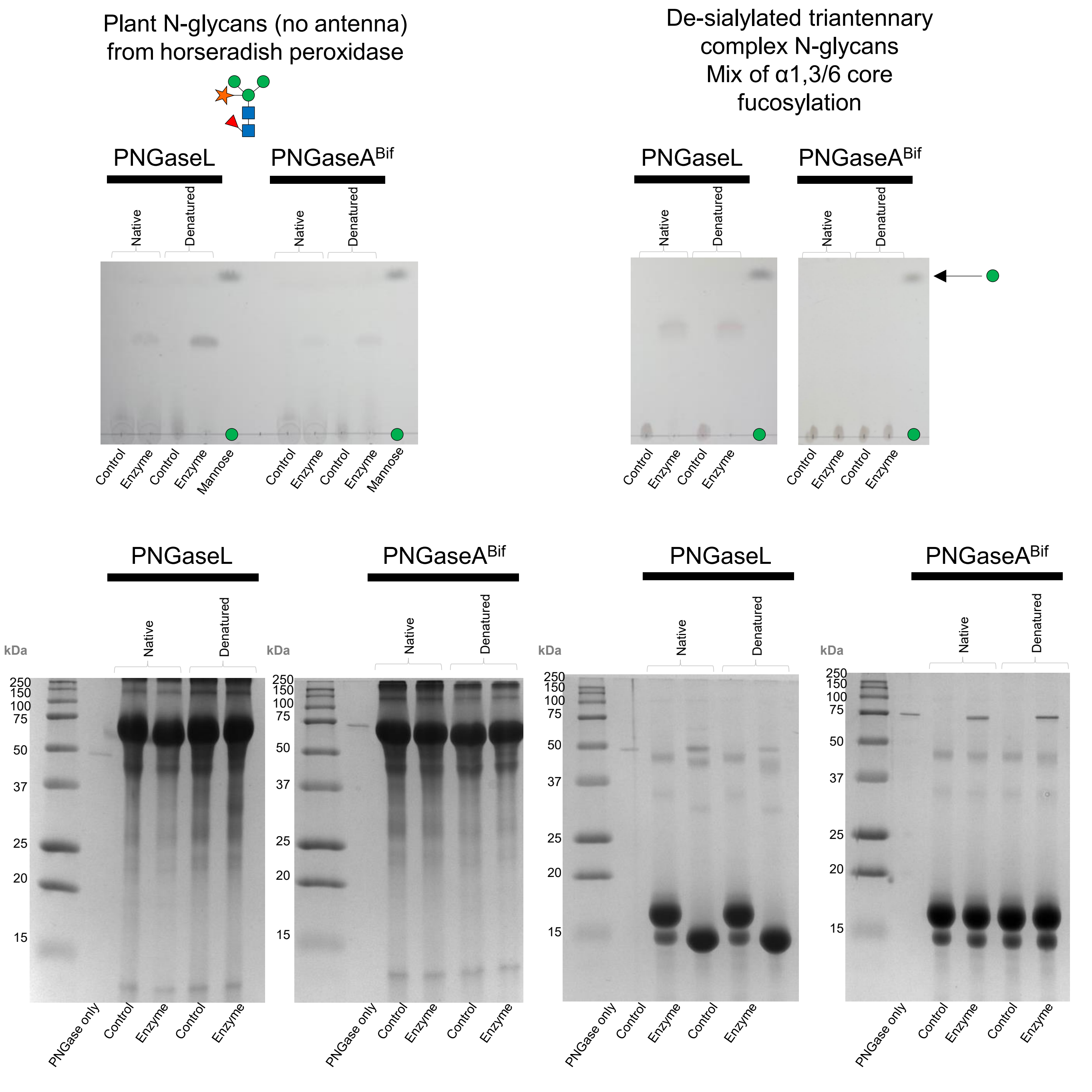

Supplementary Figure 11. Biochemical Characterisation of PNGaseA^{Bif} against plant- and insect-derived N-glycans. Horseradish peroxidase and phospholipaseA_2 from honeybee venom were used to assess the specificity of PNGaseA^{Bif} for mammalian triantennary complex N-glycans and PNGaseL was used as a positive control. The protein component of the substrate was provided to the PNGase enzymes in three forms – native, denatured (boiling), and peptide (*Vibrio coralliilyticus* treated). The N-glycan release was assessed through thin layer chromatography (top) and change in glycoprotein size was monitored through SDS-PAGE with staining for protein (bottom). PNGaseL and PNGaseA^{Bif} have molecular weights of 50.6 and 60.9 kDa, respectively. This experiment was carried out for two biological replicates.

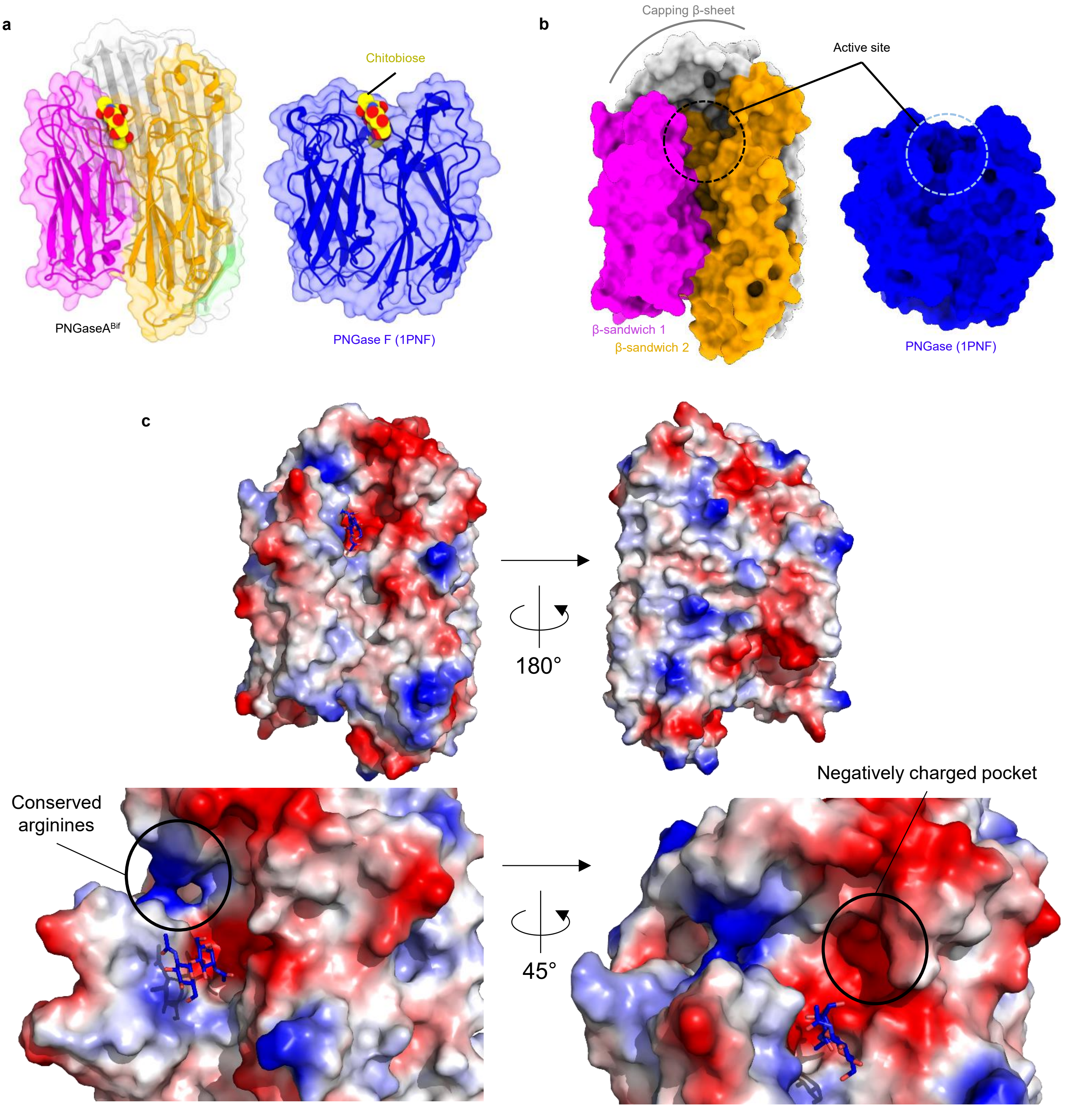

Supplementary Figure 12. Structure of PNGaseA^{Bif} in comparison to PNGaseF from *Elizabethkingia meningoseptica* a) Surface representations of PNGaseF (blue) and PNGaseA^{Bif} (pink/gold/silver) overlaid on top of a cartoon representation. Chitobiose from 1PNF is in yellow highlights the position of the active site in both enzymes. b) Surface representations of PNGaseA^{Bif} and PNGaseF to emphasise the different substrate-binding regions. c) The electrostatics of the surface of PNGaseA^{Bif} are shown, which highlights the two charged regions in the area that accommodates the peptide component of the substrate.

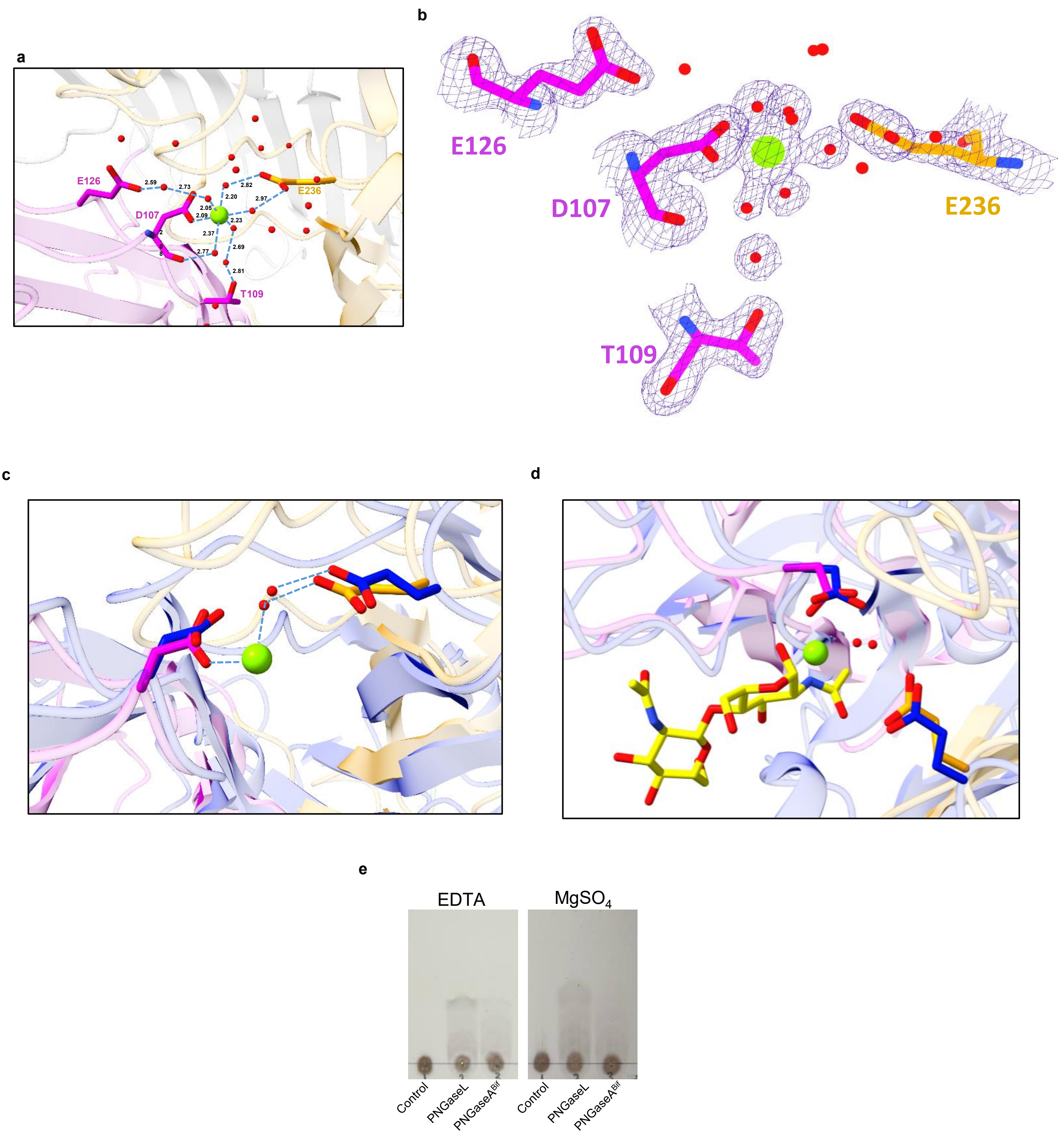

Supplementary Figure 13. Analysis of the Magnesium ion in the active site of the apo structure. a) Residues and water molecules within the active site of PNGaseA^{Bif} are shown. The Mg^{2+} ion (green) is coordinated by the carboxylate oxygen of the side chain and 5 waters in octahedral geometry. The 5 waters are then hydrogen bonded directly or indirectly to the side chains of glutamic acid 126, glutamic acid 236, threonine 109, and the backbone carbonyl of aspartic acid 107. Dashed lines are shown for direct and indirect interactions, with distances shown in Angstroms. b) The 2Fo-Fc electron density map (blue mesh) is shown at 1.5 σ within 1.5 Å distance of E126, D107, E236, T109 and waters involved in the coordination. The electron density strongly supports the octahedral geometry and accurate placement of the water molecules. c) Superposition of the conserved Asp and Glu residues shows a coordinating water modelled in the same position in the PNGaseF structure. d) Superposition of the chitobiose moity on the PNGaseA^{Bif} structure shows that the magnesium atom in the catalytic site is found in close proximity to where the reducing end of the glycan is expected to be. e) Assays against Bovine RNaseB was used to assess the requirement for metal of PNGaseA^{Bif} for high-mannose N-glycans. PNGaseL was used as a positive control. The results were assessed by thin layer chromatography.

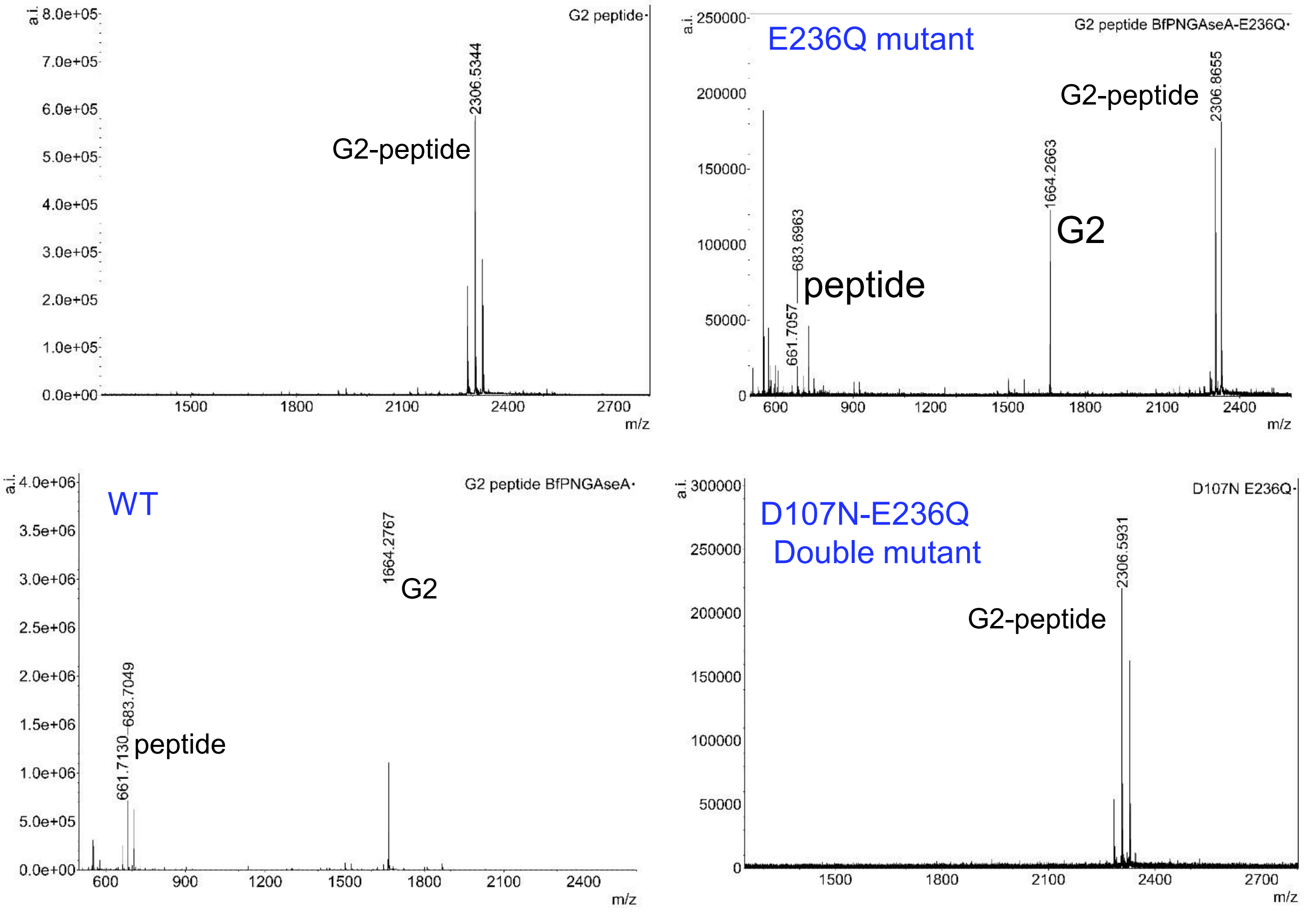

Supplementary Figure 14. MALDI-TOF analysis of G2-peptide processing by WT and mutant PNGaseA^{Bif}. Representative MALDI-TOF mass spectra of the G2-peptide substrate alone and after incubation with PNGaseABif WT, E236Q, or the D107N–E236Q double mutant. Peaks corresponding to the intact G2-peptide substrate, the released G2 glycan, and the deglycosylated peptide are indicated. Panel order follows the figure layout: top left, G2-peptide alone; top right, G2-peptide incubated with E236Q; bottom left, G2-peptide incubated with WT PNGaseABif; bottom right, G2-peptide incubated with the D107N–E236Q double mutant. MALDI-TOF MS analyses were performed in one or two independent experiments, depending on the sample. Source data are provided in the Source Data file.

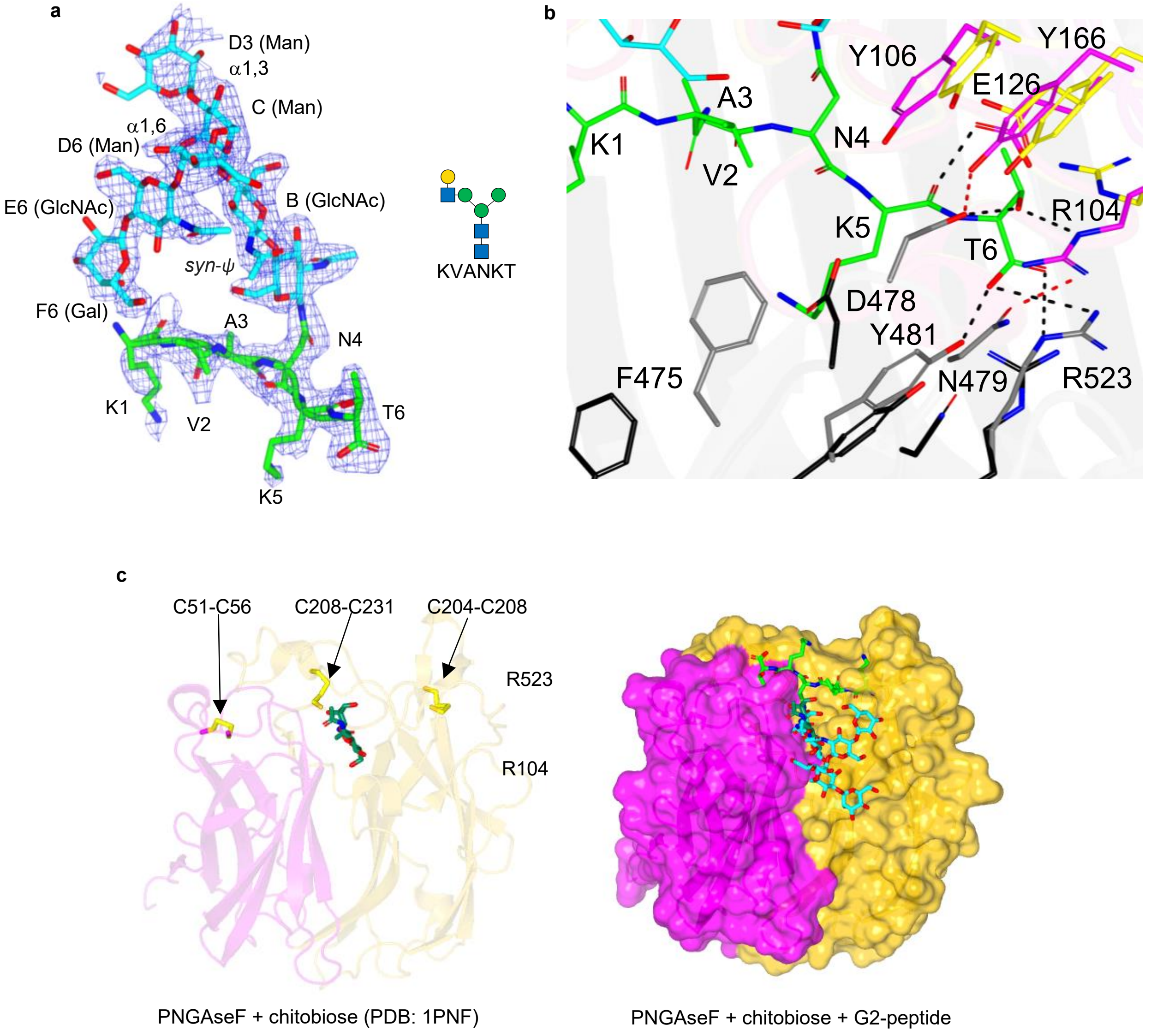

Supplementary Figure 15. Structural details of the G2-peptide substrate, the enzyme:substrate interactions in the active site, and comparison with PNGaseF. a) Electron density maps are Fo–Fc (blue), contoured at 2.2σ, for the bound G2-peptide. Labels for the resolved sugar units are shown. Following the arm-specific nomenclature, residues in the α1,3 arm are designated D3, E3 and F3, whereas the corresponding residues in the α1,6 arm are designated D6, E6 and F6. No electron density was observed for E3 and F3. The glycosylated Asn adopts a syn-ψ conformation, consistent with the preferred conformation of the chitobiose core. b) Close-up view of the protein–ligand interaction network in the active site. Residues from the apo structure (black and gold) and from the PNGaseABif D107N–E236Q–G2-peptide complex (grey and magenta) are overlaid to highlight local side-chain rearrangements associated with binding of the K1–T6 peptide segment and the glycan. Red dashed lines indicate hydrogen bonds involving residues from β-sandwich 1 and the β-sheet cradle, whereas black dashed lines indicate hydrogen bonds between the peptide and enzyme residues. c) Comparison with PNGaseF. Left, structure of PNGaseF bound to chitobiose (PDB: 1PNF). Right, PNGaseF displayed together with the G2-peptide for comparison, illustrating the more open peptide-binding region relative to PNGaseABif.

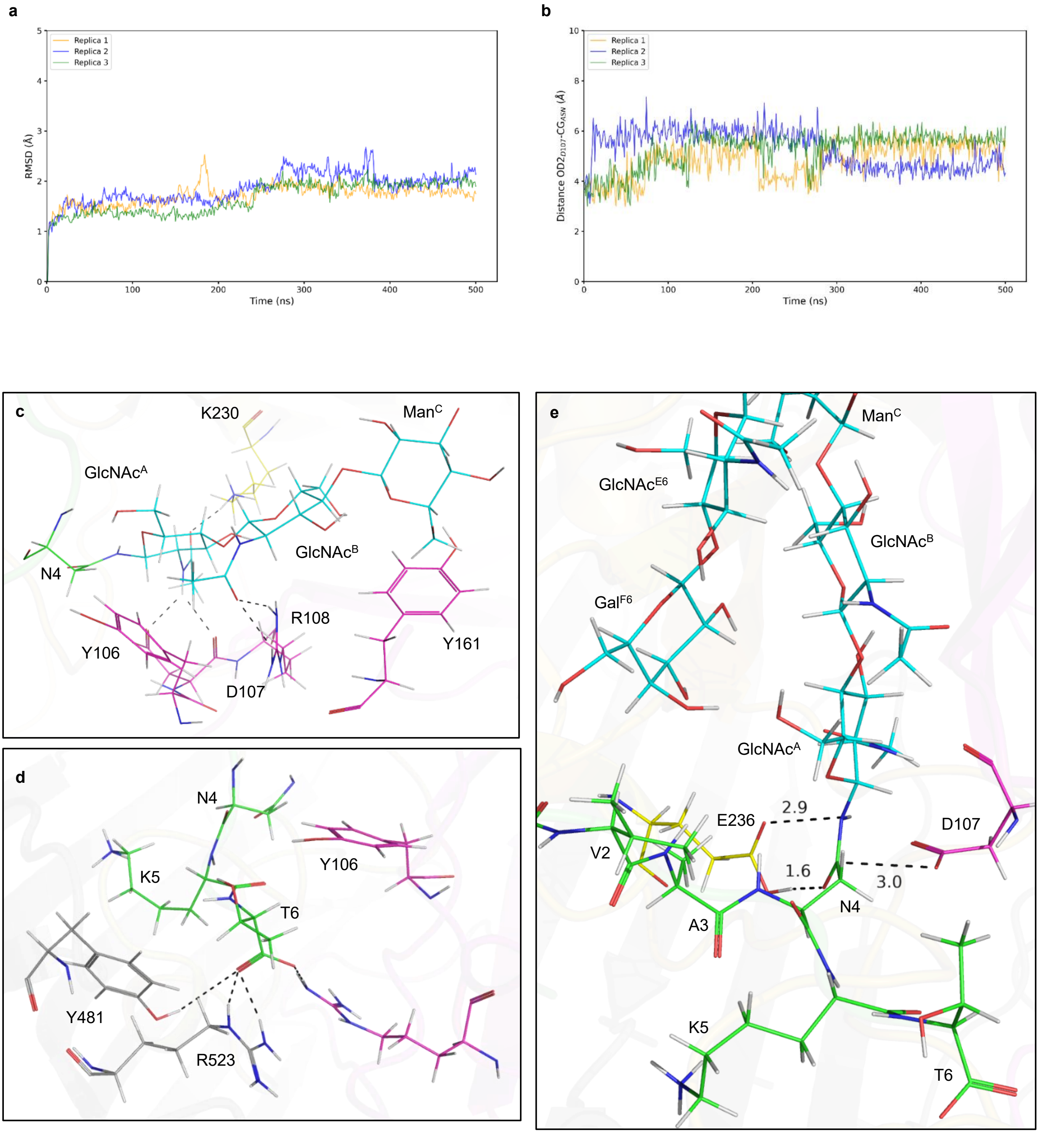

Supplementary Figure 16. Stability of PNGaseBif-G2-peptide complex during molecular dynamics simulations. Backbone RMSD (top, panel a) and distance between catalytic Asp107 and Asn4 carbonyl (bottom, panel b) for three independent molecular dynamics simulations of the complex over 500 ns. c) Snapshot from replica 1 showing main interactions of the chitobiose core with PNGaseBif. Colours are as in Fig. 4. d) Same snapshot showing main interactions of Thr6 from G2-peptide with PNGaseBif. Colours are as in Fig 4. e) Snapshot from replica 1 showing catalytic distances for a plausible direct nucleophilic attack by Asp107. Colours are as in Fig. 4.

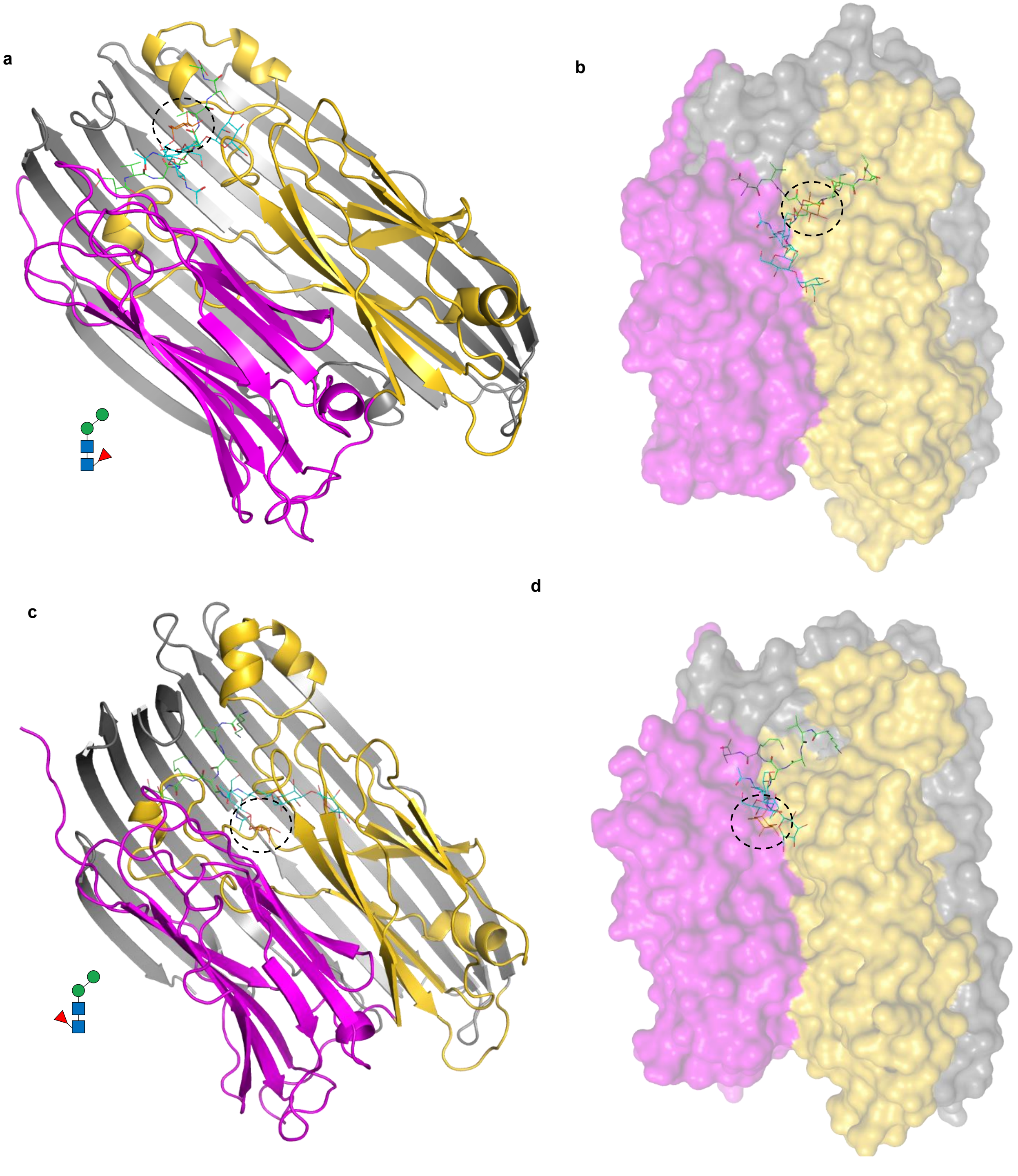

Supplementary Figure 17. Modelling of core α1,6 and α1,3-fucose in the PNGaseA^{Bif} G2-peptide complex. a) Cartoon representation of the PNGaseABif–G2-peptide complex with a modelled core α1,6-fucose, shown as orange sticks, attached to the innermost GlcNAc. Domain colours are as in Fig. 4, with β-sandwich 1 in magenta, β-sandwich 2 in gold, and the β-sheet cradle in grey. The model shows that the added fucose projects into a solvent-exposed region without obvious steric clashes with the protein. b) Surface representation of the same model, highlighting the solvent-exposed position of the modelled core α1,6-fucose within the binding cleft. This supports the structural interpretation that core fucosylation can be accommodated without major interference with ligand binding. c) Cartoon representation of the PNGaseABif–G2-peptide complex with a modelled core α1,3-fucose, also in orange sticks, attached to the innermost GlcNAc. Domain colours are as in Fig. 4, with β-sandwich 1 in magenta, β-sandwich 2 in gold, and the β-sheet cradle in grey. The model shows that the added fucose projects into a solvent-exposed region without obvious steric clashes with the protein. d) Surface representation of the same model, highlighting the solvent-exposed position of the modelled core α1,3-fucose within the binding cleft.

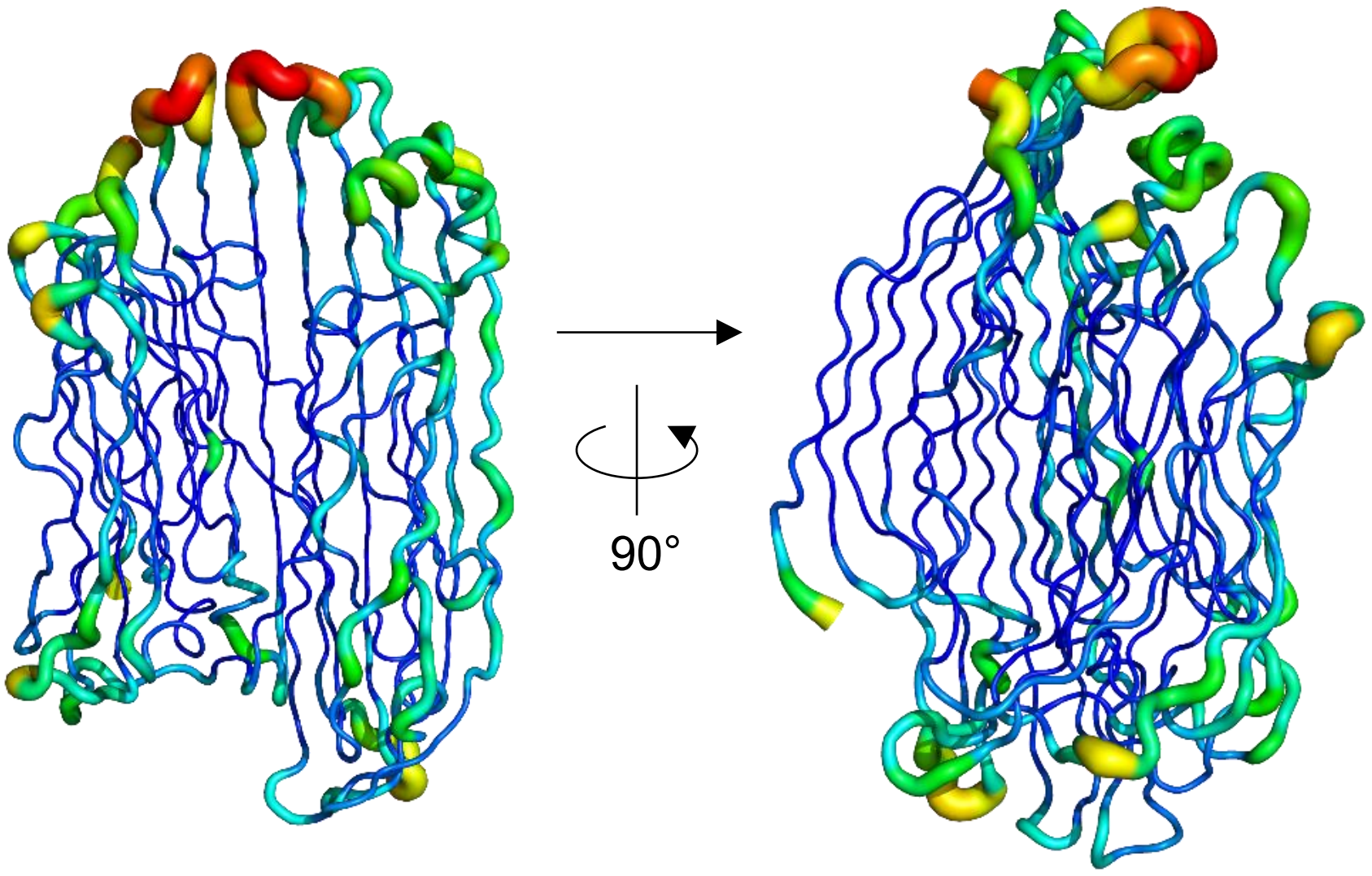

Supplementary Figure 18. Analysis of the PNGaseA^{Bif} structure. B-factors of the PNGaseA^{Bif} structure shows that the loops of the cradle nearest the active site have flexibility.

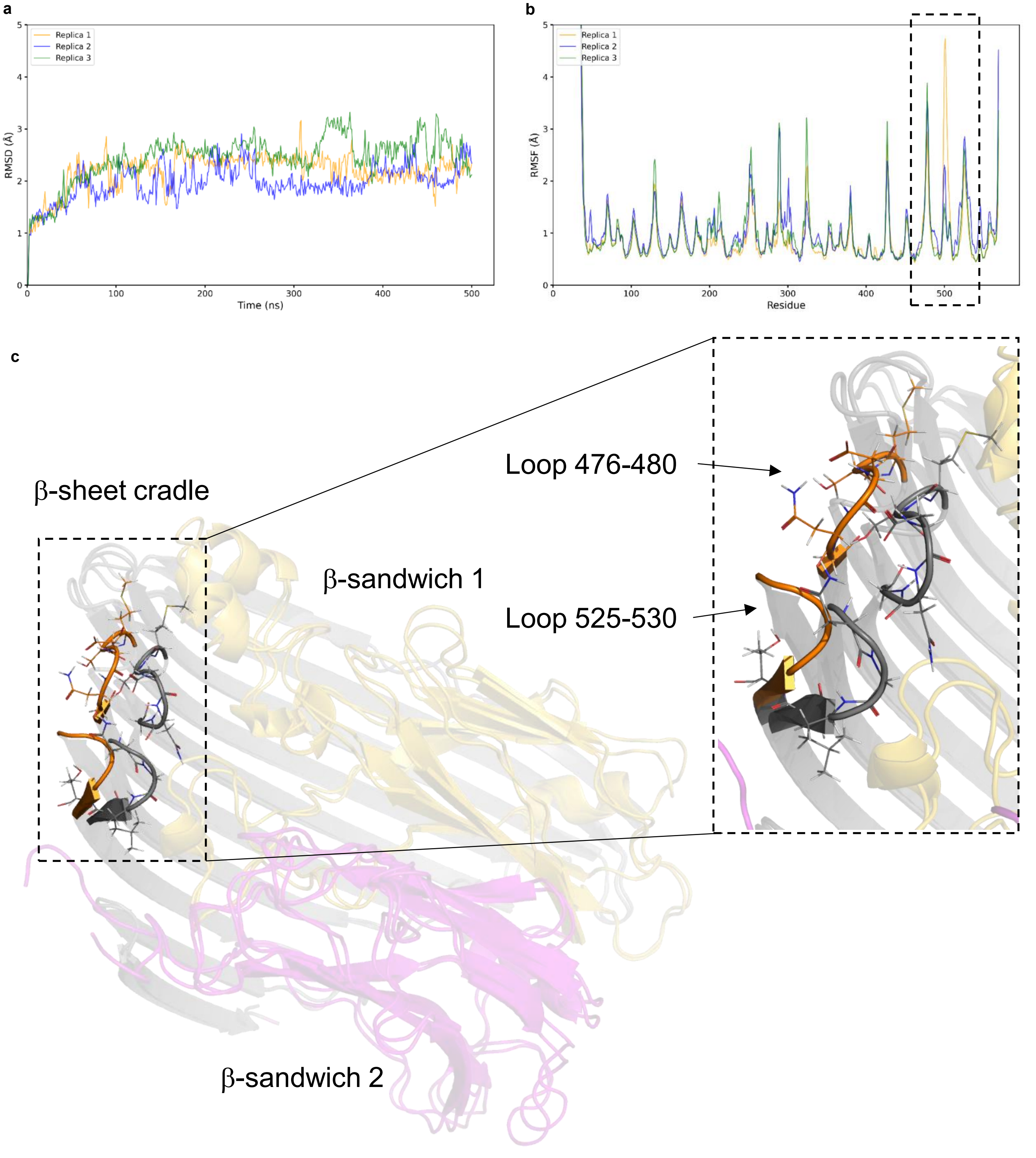

Supplementary Figure 19. Cradle-loop mobility in apo PNGaseABif during molecular dynamics simulations. Backbone RMSD (top, panel a) and per-residue RMSF (bottom, panel b) for three independent molecular dynamics simulations of the apo enzyme over 500 ns. Replicas are shown in orange, blue, and green, respectively. The dashed box in the RMSF plot highlights residues in the mobile cradle region, including loop 476–480 and loop 525–530. c) Superposition of frames 10 (grey cartoon) and 450 (orange cartoon) from apo replica 1, showing conformational changes in the β-sheet cradle relative to β-sandwich 1 and β-sandwich 2. The inset highlights the displacement of loop 476–480 and loop 525–530, which contribute to opening and closing motions of the cradle region.

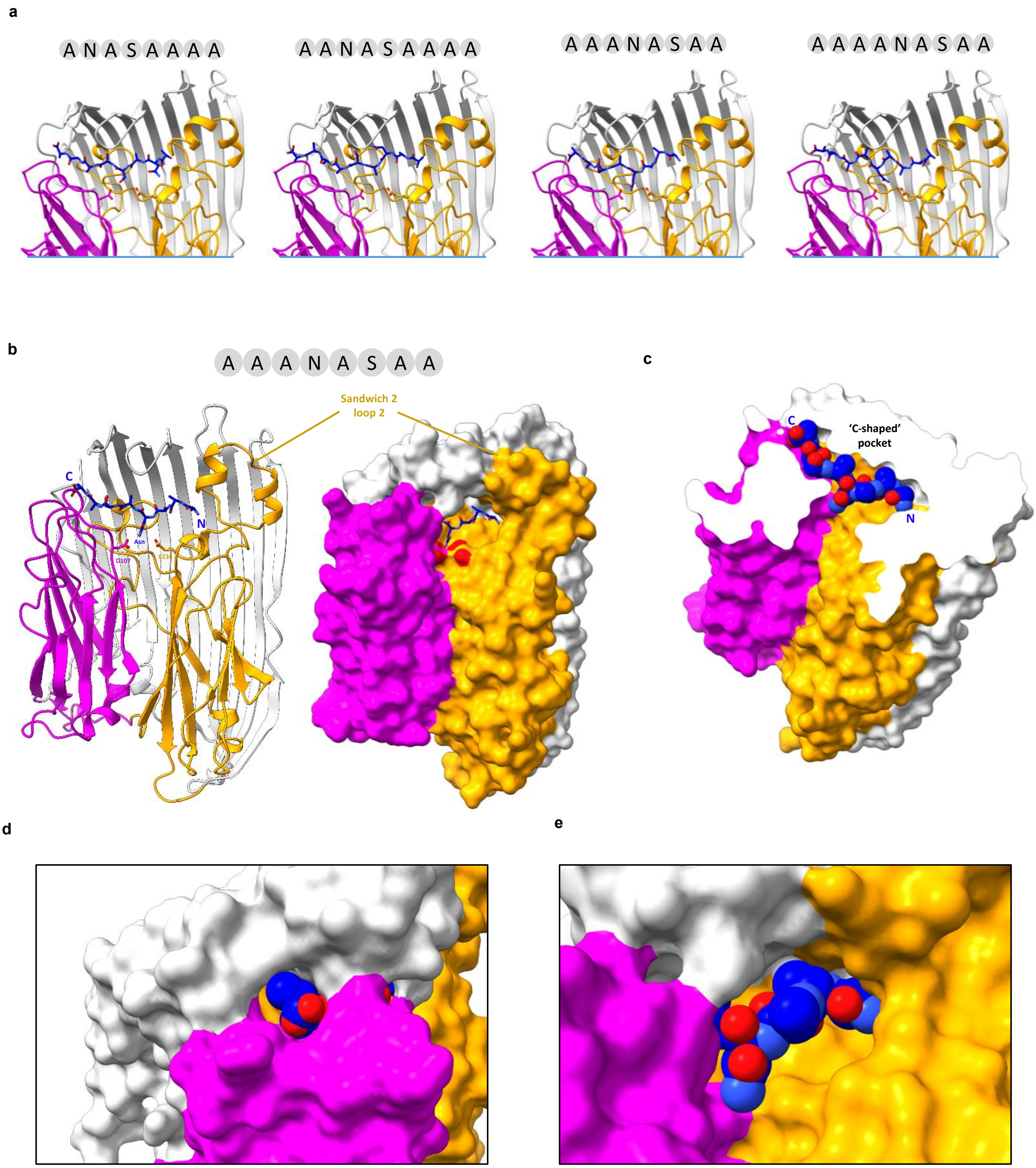

Supplementary Figure 20. Modelling of peptides into the C-shaped poket. a) Four Alphafold 3 models of different peptides (blue and hetero atom colouring) containing the core Asn-Ala-Ser motif. Four predictions are shown with different numbers of Ala residues N- and C-terminal to the NAS motif. Only models with three or four N-terminal residues allow for the N-glycan to reside in proximity to the catalytic residues D107 and E236. b) The AAANASAA peptide is shown in the context of the full protein and its encapsulation within the β-sheet cradle. c) A clipped model shows the peptide's accommodation within the 'C-shaped' pocket. The pocket would allow for small or hydrophobic N+1, N-2 and N-3 residues. d) The C-terminus of the peptide is observed exiting the pocket via the open end of the pocket. e) The N-terminus is buried within the closed end of the pocket formed by sandwich 2 loop 2.

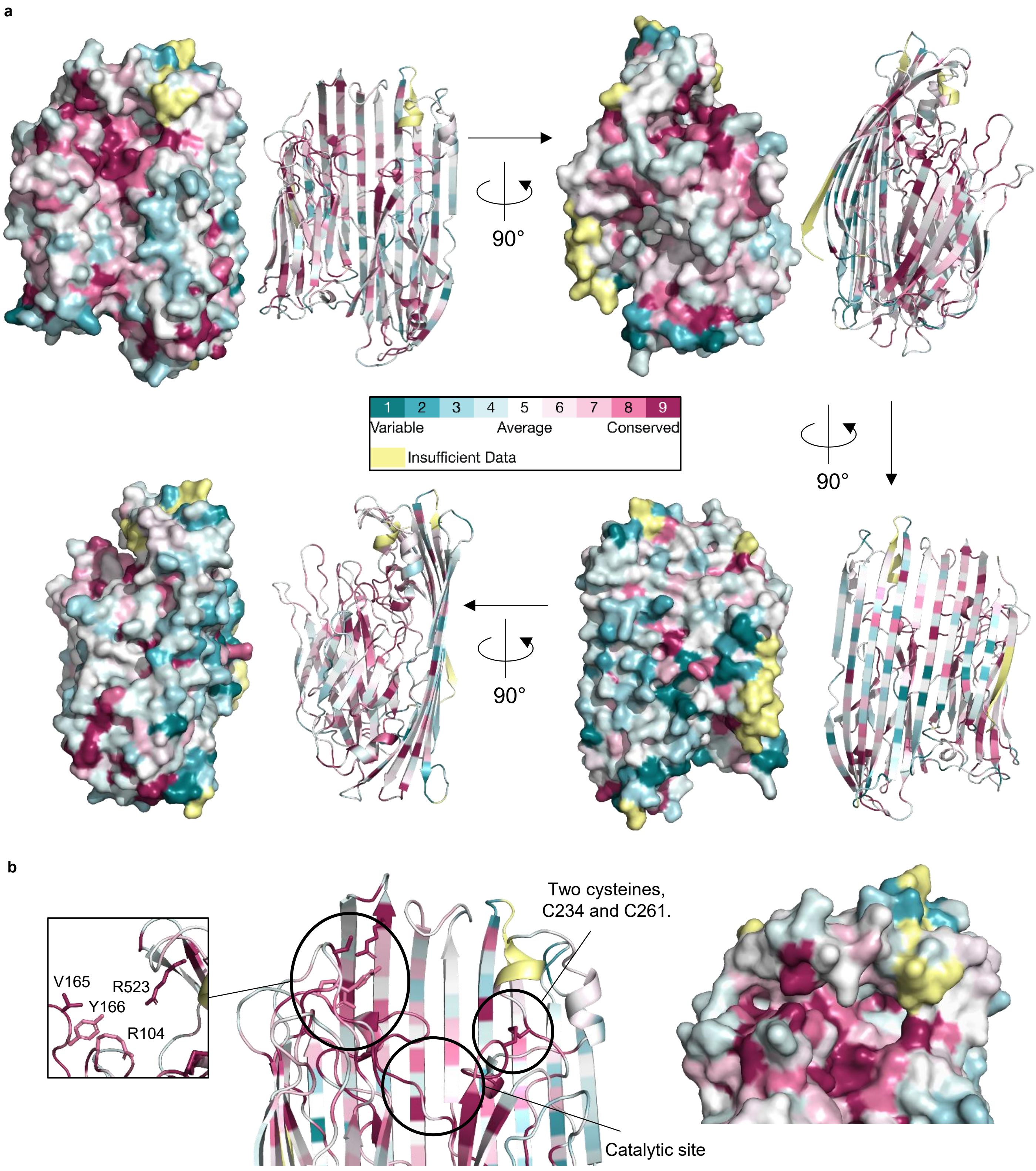

Supplementary Figure 21. Analysis of the conservation of residues across homologues mapped onto the PNGaseA^{Bif} structure. a) Consurf was used to analyse the conservation of residues over the PNGaseA^{Bif} structure. The highest conservation of residues is focussed on the active site. b) Consurf analysis of PNGaseA^{Bif} structure that indicates how highly residues are conserved throughout a structure. Three highly conserved regions were identified in the region of the protein where the active site is.

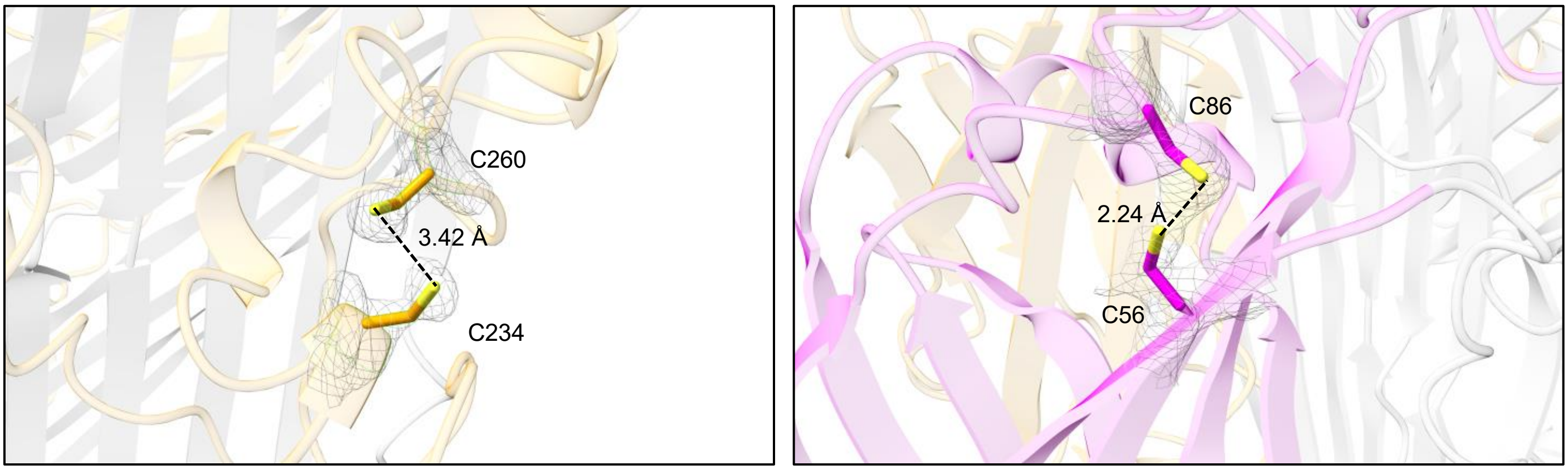

Supplementary Figure 22. Cysteine/Cystine within PNGase A^{bif}. a) The 2Fo-Fc electron density map (mesh) is shown at 2 σ within 2 Å distance of C260 and C234. The density and distance between the sulphur atoms do not support a disulphide bond. b) The 2Fo-Fc electron density map (mesh) is shown at 1.5 σ within 2 Å distance of C56 and C86. The density suggests multiple conformations of the Cys residues, with a 0.58:0.42 ratio between cystine and cysteine forms modelled.

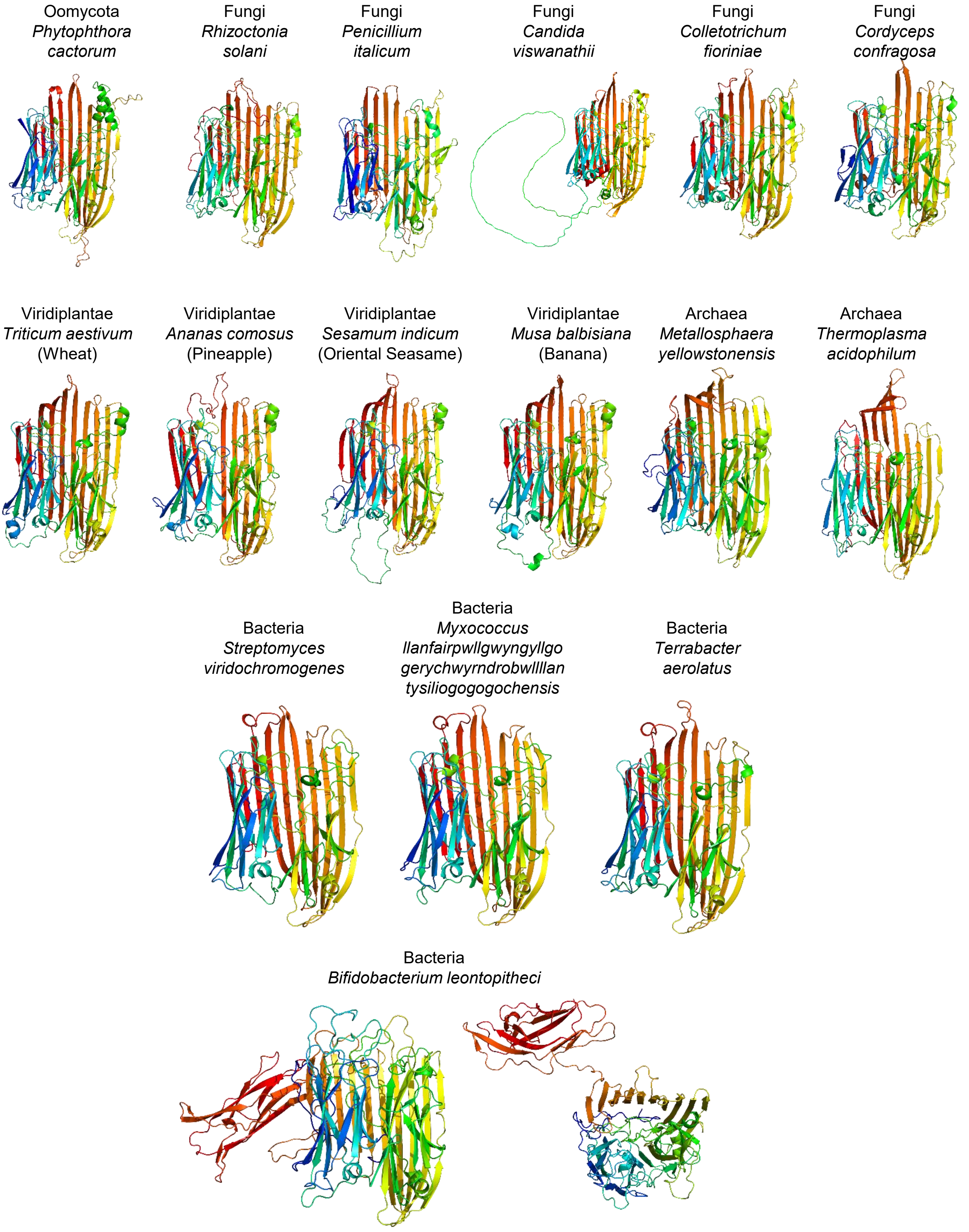

Supplementary Figure 23. Models of the structures of PNGaseA enzymes from a variety of organisms. The models are displayed as cartoons with the N- and C-termini colours blue to red, respectively. All structures show the canonical two eight-stranded β-sandwich catalytic module and also the cradle, analogous to the PNGaseA^{Bif} structure. Source data are provided in the Source Data file.

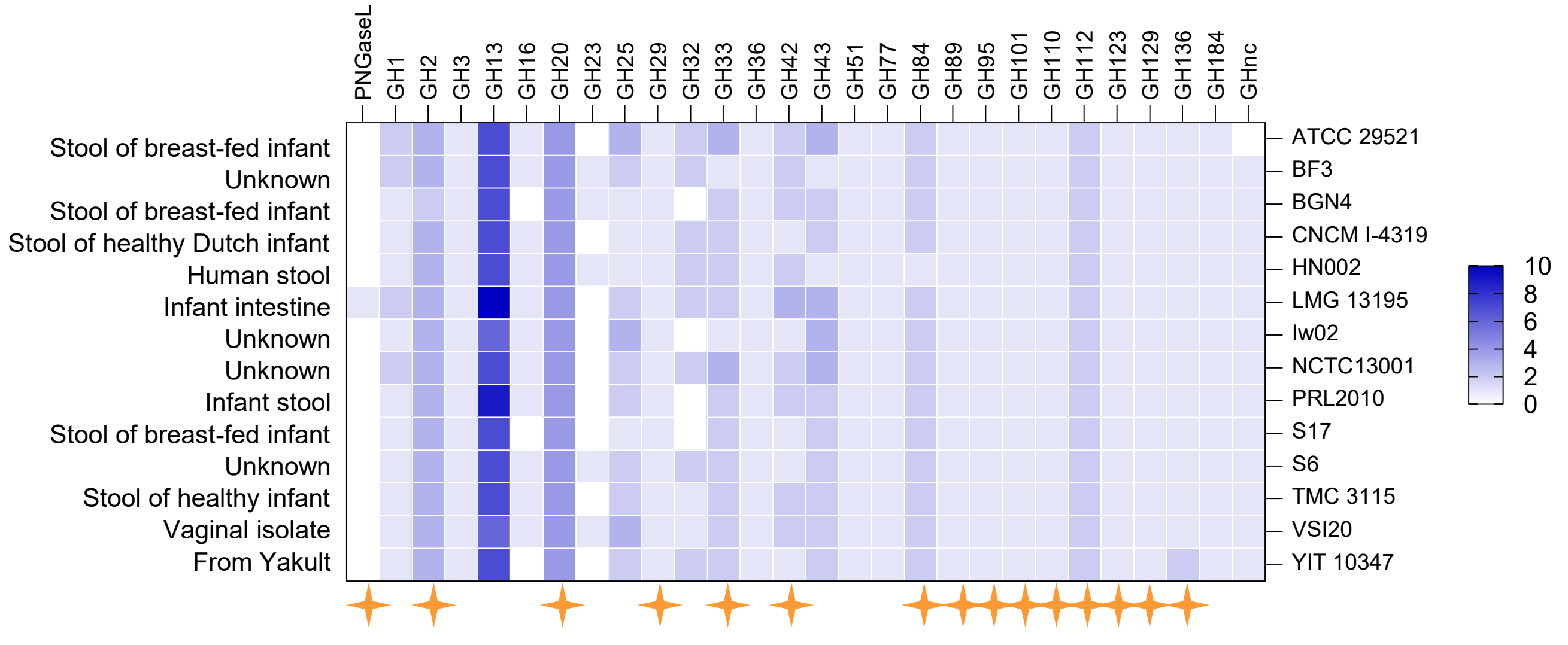

Supplementary Figure 24. Frequency of putative CAZymes across different *Bifidobacterium bifidum* strains. The heat map shows how many different GH and PNGase families are present across different strains (right). The origin of the strain is indicated on the left and the orange four-pointed star indicates the enzyme families associated with host glycan breakdown. Source data are provided in the Source Data file.

Supplementary Figure 25. Full disclosure of TLCs and SDS-PAGE gels.

Figure 1 & Supplementary Figure 3

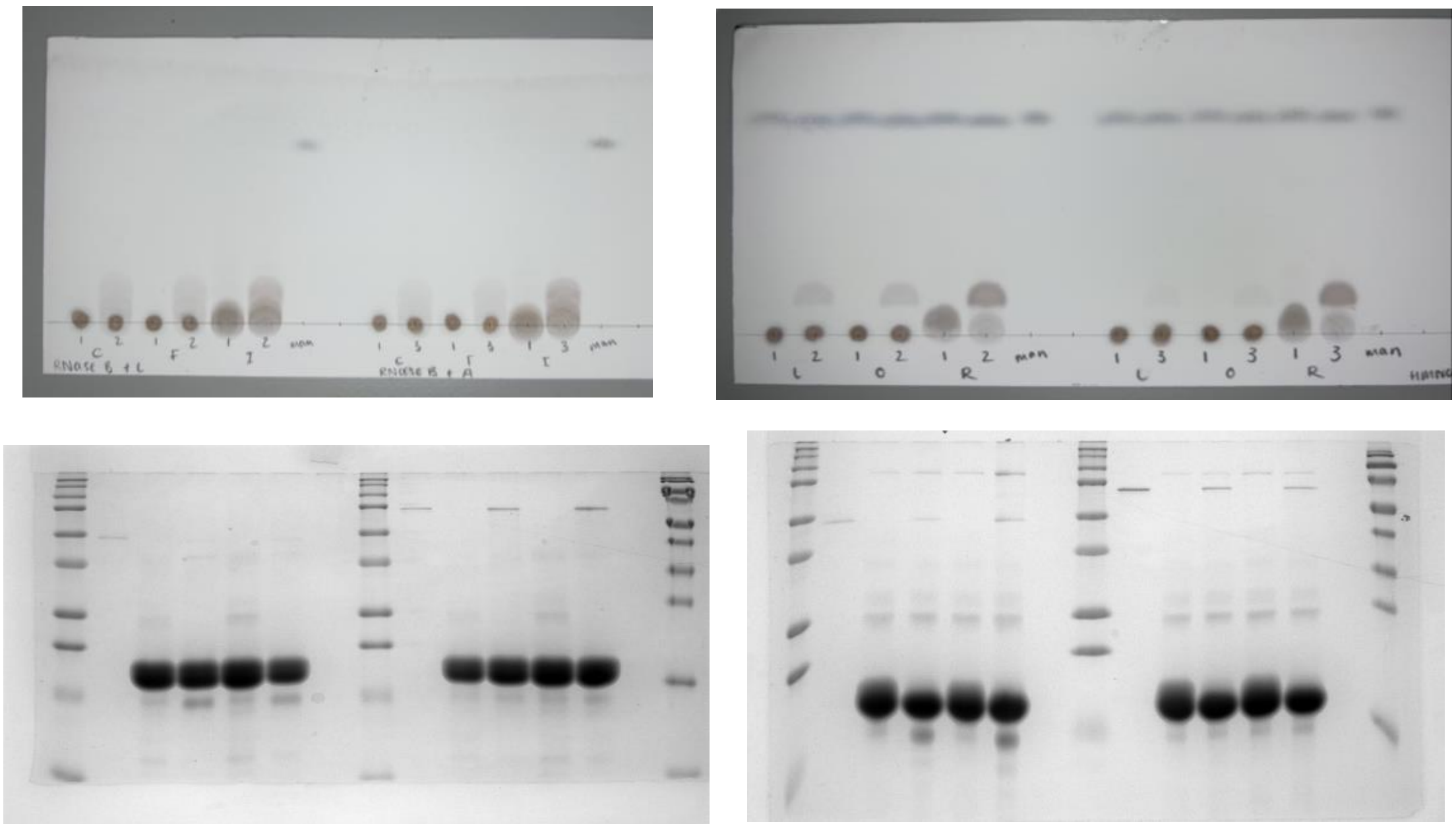

Figure 1 & Supplementary Figure 4

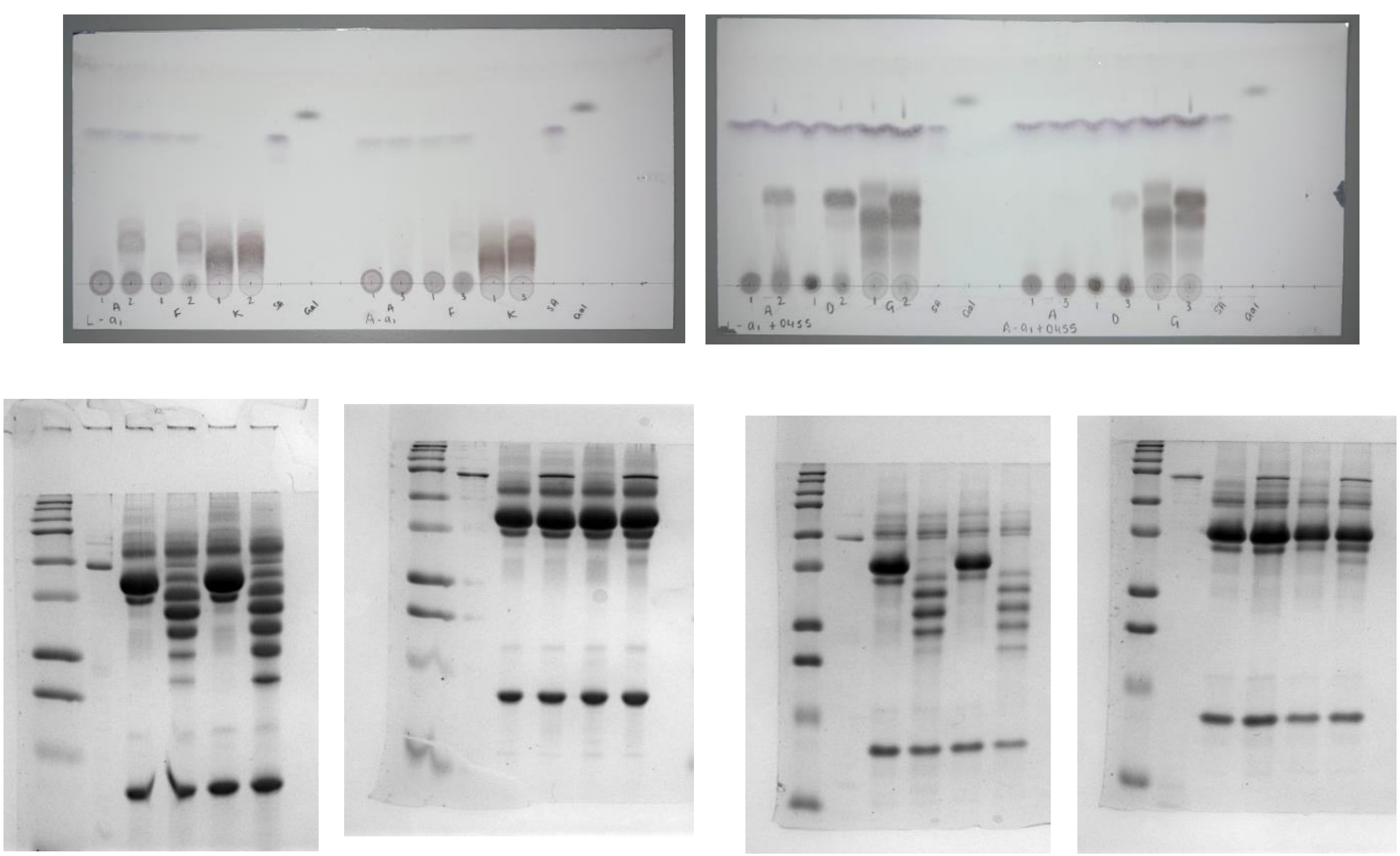

Figure 1 & Supplementary Figure 5

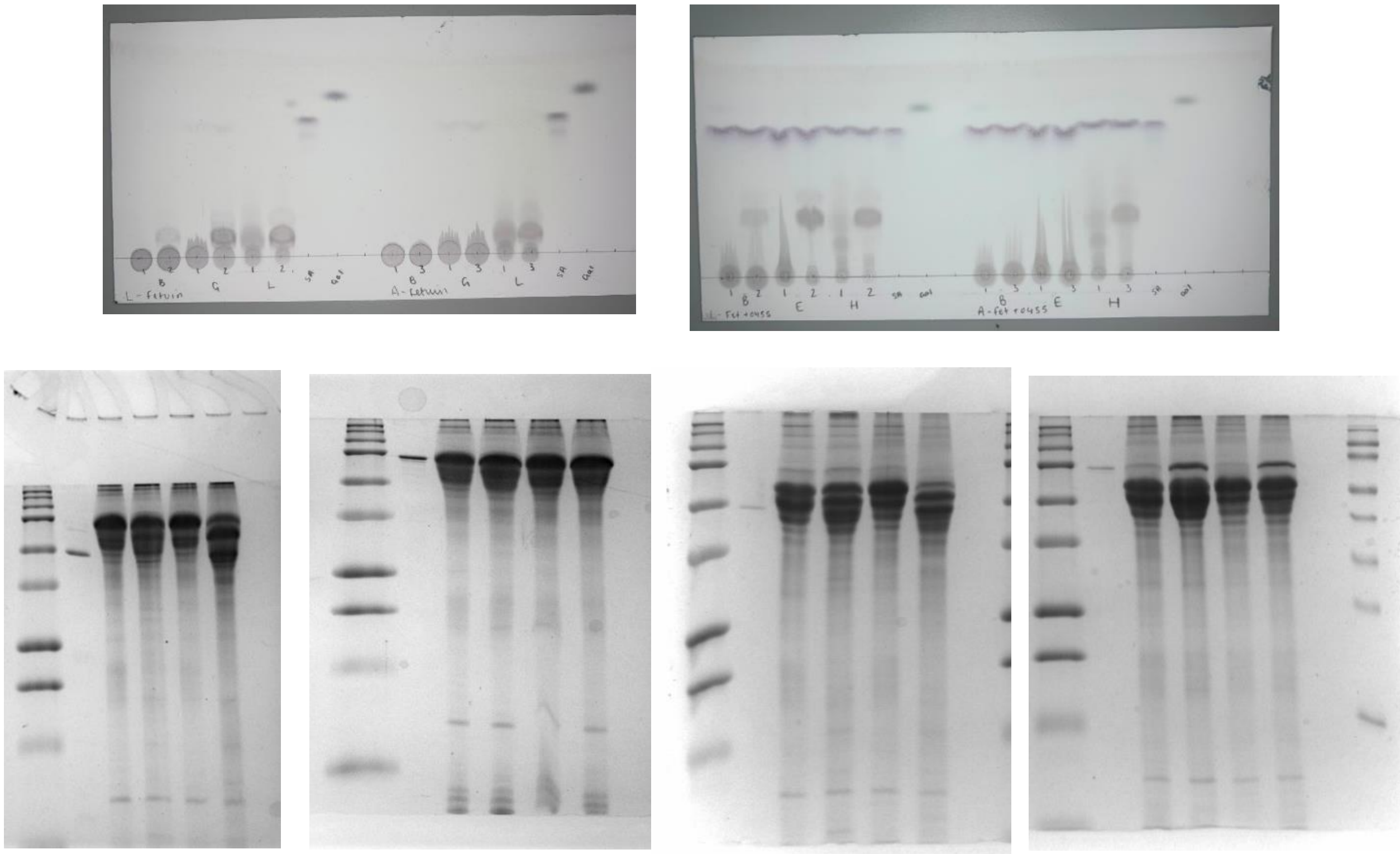

Supplementary Figure 8

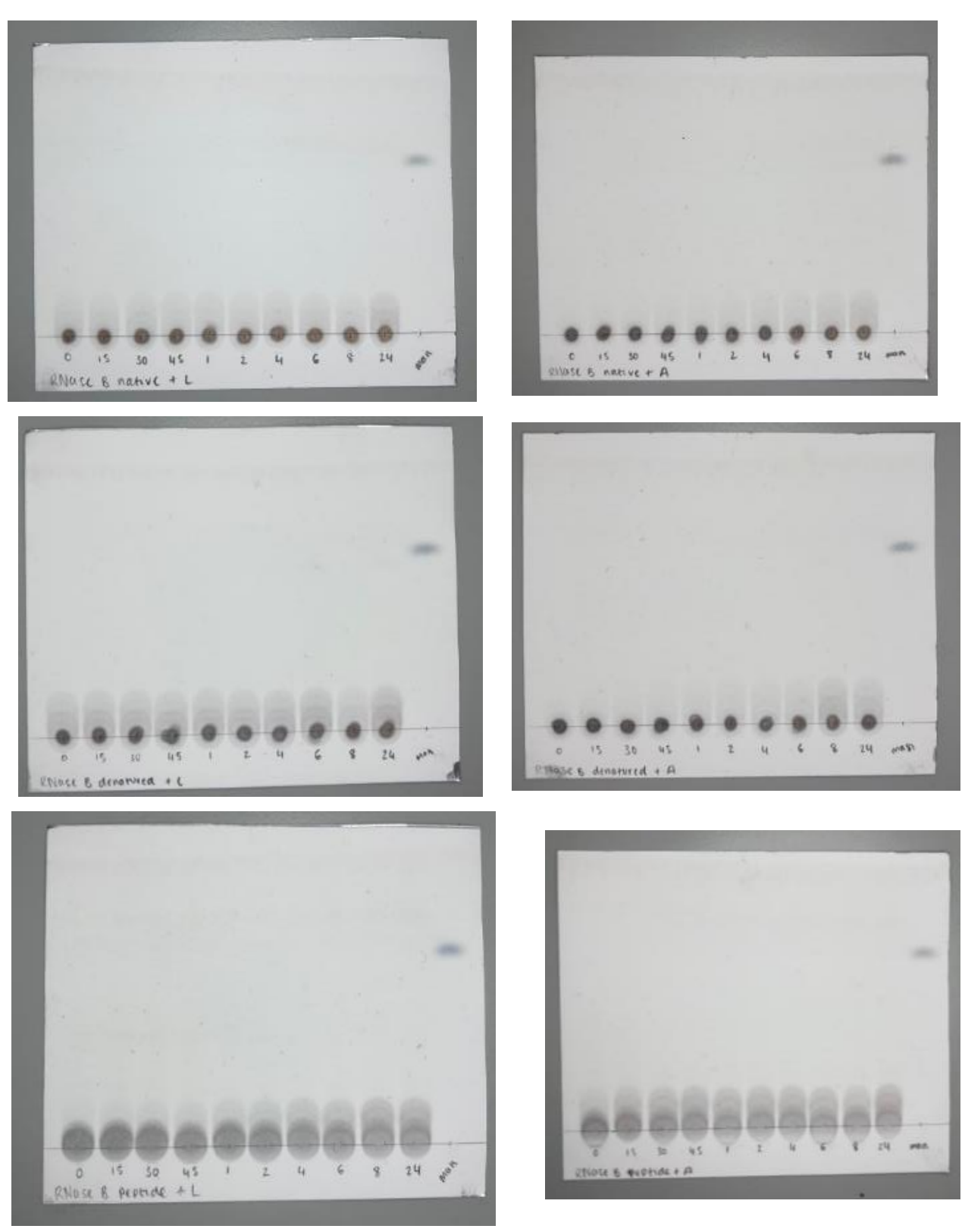

Supplementary Figure 9

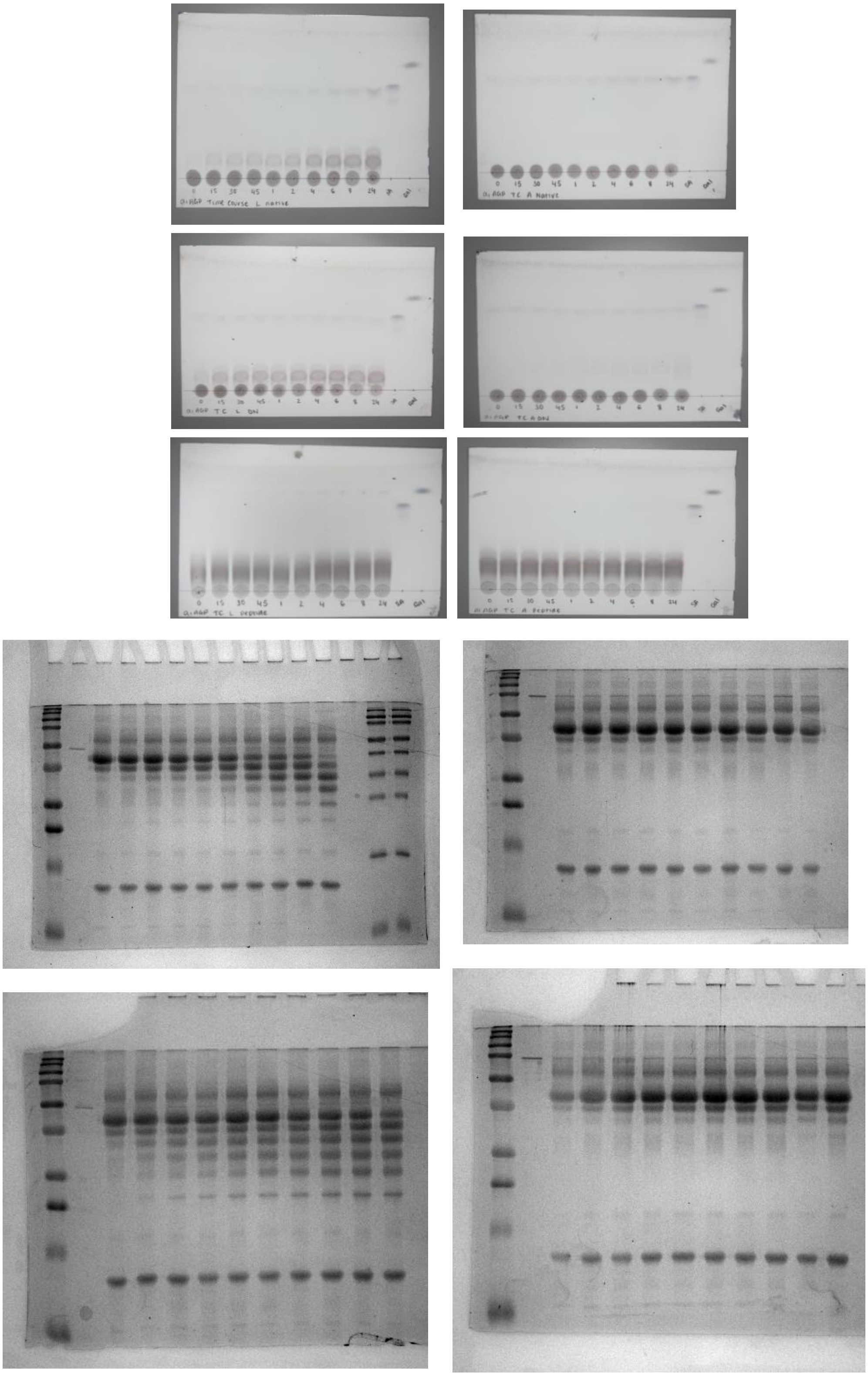

Supplementary Figure 11

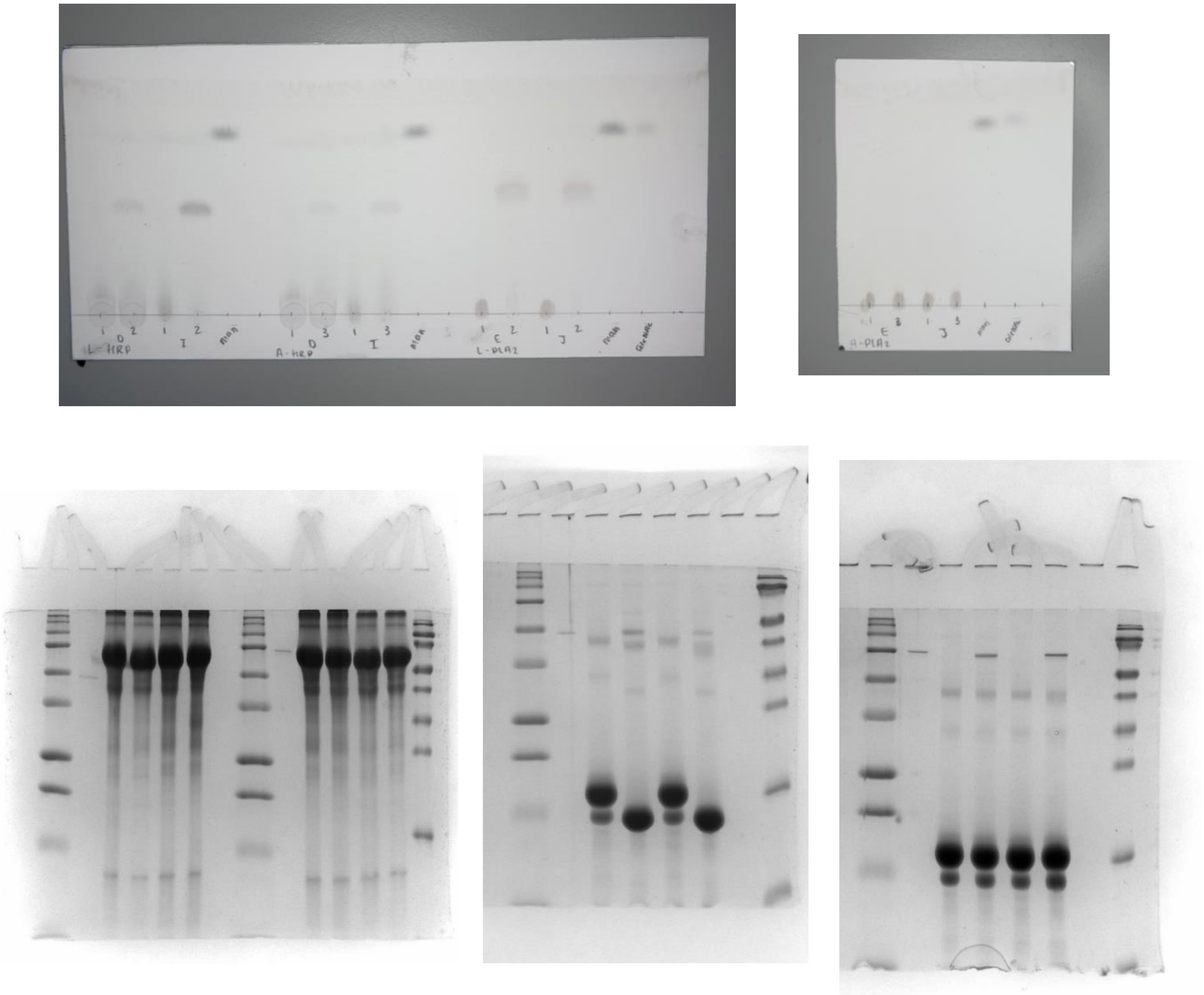

Supplementary Figure 13

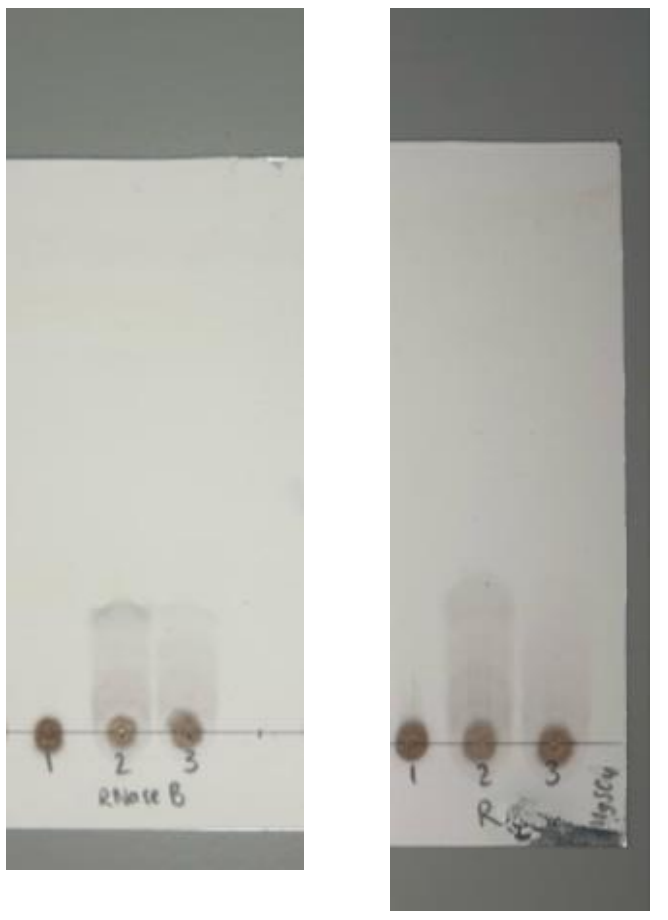

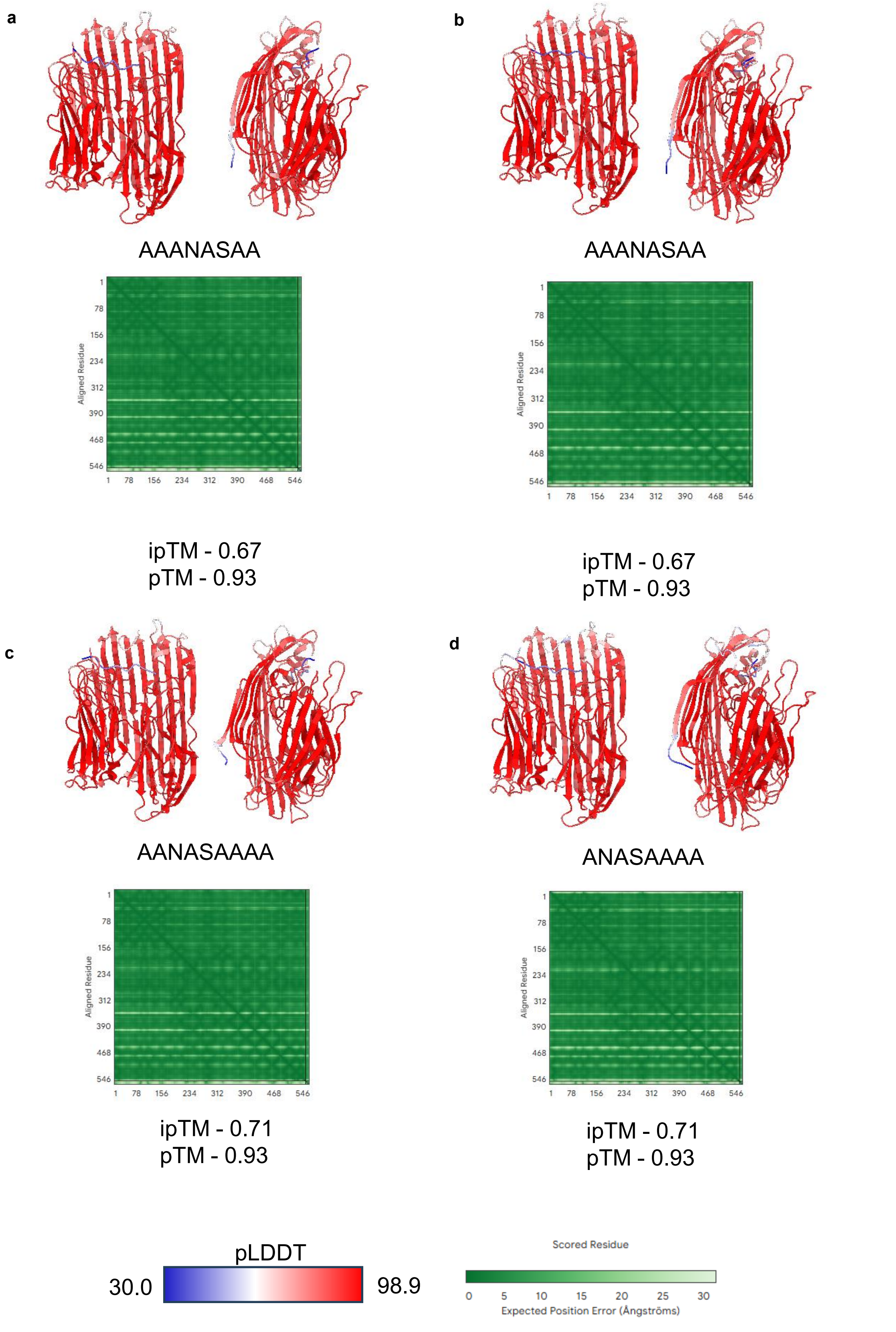

Supplementary Figure 26. Alphafold 3 modelling of substrate – pLDDT and predicted aligned error (PAE) disclosure. PNGaseA^{Bif} was modelled with 4 substrate mimic peptide sequences: A) AAANASAA. B) AAANASAA. C) AANASAAAA. D) ANASAAAA. All models are shown in cartoon form, with two orientations coloured by the pLDDT. The predicted aligned error is shown and pTM and ipTM scores are given for each modelled pair.

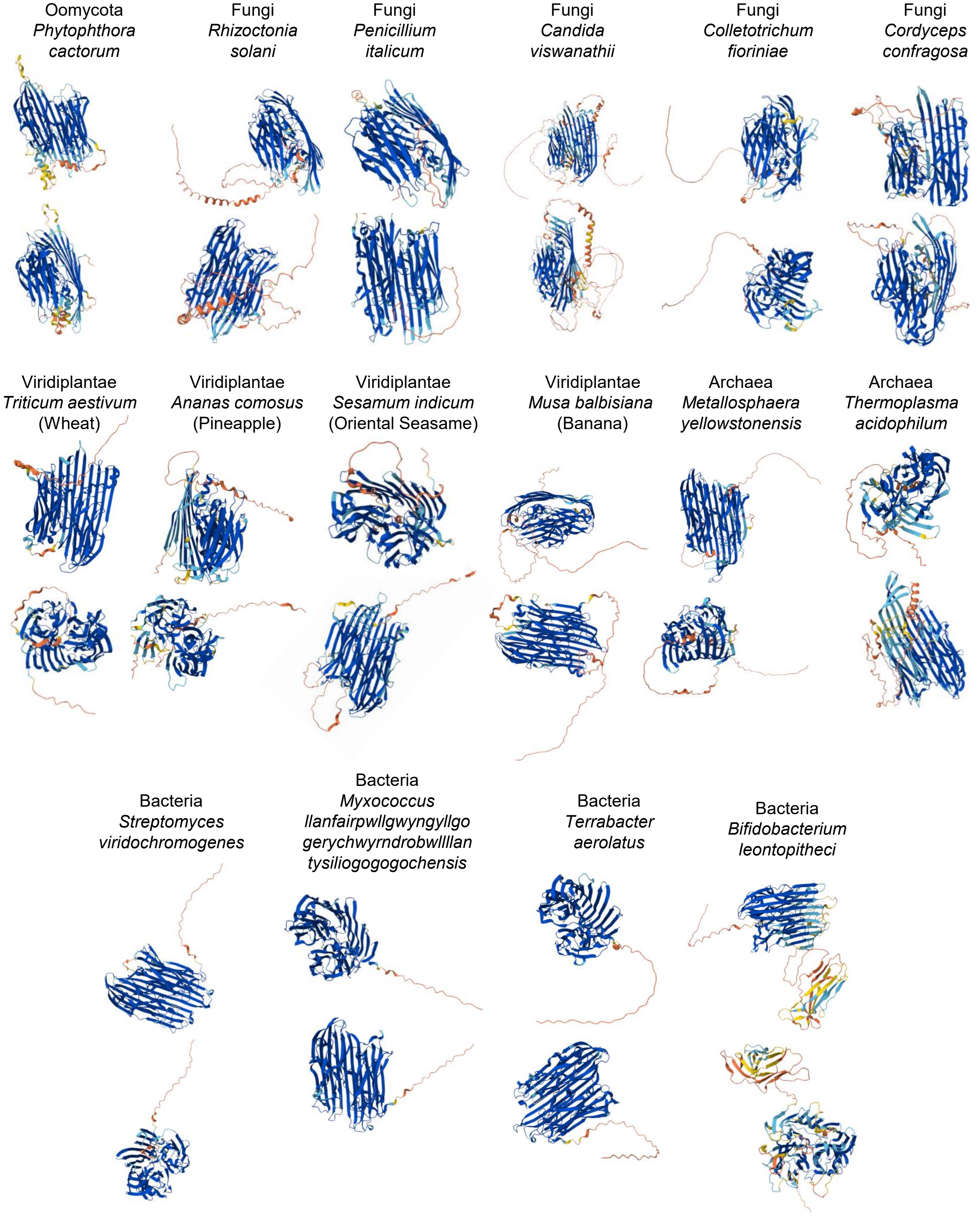

Supplementary Figure 27. pLDDT of models of the structures of PNGaseA enzymes from a variety of organisms for Supplementary Figure 23. The models are displayed as cartoons from two different angles.

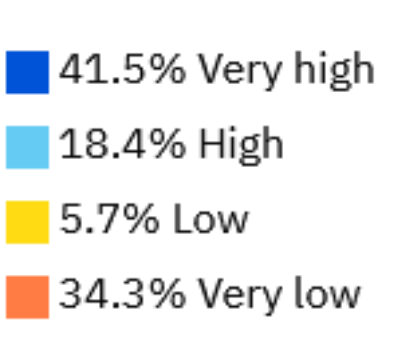

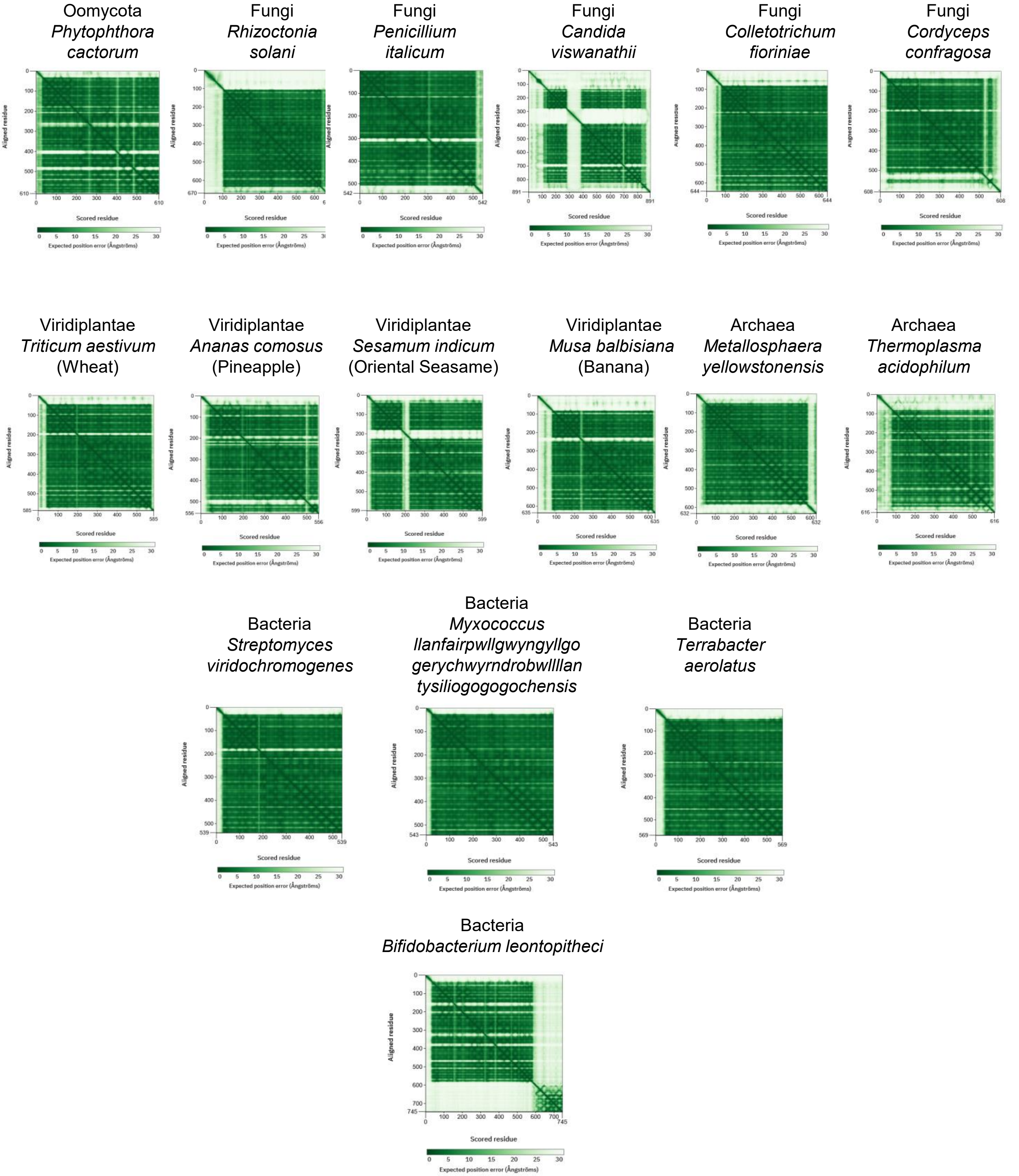

Supplementary Figure 28. Predicted aligned error (PAE) for each model Models of the structures of PNGaseA enzymes from a variety of organisms for Supplementary Figure 23.

Supplementary Table 1. *B. bifidum* strain information used to analyse the variable region where *pngaseA*^{bif} resides in LMG13195

Accession no.	Strain Name	NCBI Submitter	Genbank	Refseq	Date Published	Date Accessed
NZ_CP058603.1	CNCM I-4319	University College Cork	GCA_017894325.1	GCF_017894325.1	09-Apr-2021	22-May-2025
NZ_LR134344.1	NCTC13001	SC	GCA_900637095.1	GCF_900637095.1	20-Dec-2018	22-May-2025
NZ_AP012323.1	JCM 1255	Graduate School of Frontier Sciences, University of Tokyo	GCA_001025135.1	GCF_001025135.1	24-Mar-2015	22-May-2025
NZ_CP169560.1	Bg41221_3D10	Pathology and Immunology, Washington University School of Medicine in St. Louis	GCA_050308165.1	GCF_050308165.1	14-May-2025	04-Jun-2025
NZ_CP022723.1	S6	Korea Food Research Institute	GCA_003390735.1	GCF_003390735.1	15-Aug-2018	22-May-2025
NZ_LR698991.1	MGYG-HGUT-02396	EMG	GCA_902386775.1	GCF_902386775.1	10-Aug-2019	04-Jun-2025
NZ_CP149430.1	lw02	Peking University School and Hospital of Stomotology, Peking University	GCA_045290295.1	GCF_045290295.1	26-Nov-2024	04-Jun-2025
NZ_CP018757.1	PRI 1	Institute for Basic Science (IBS) and Pohang University of Science and Technology (POSTECH)	GCA_002845845.1	GCF_002845845.1	20-Dec-2017	04-Jun-2025
NZ_CP118071.1	VSI20	Seattle University	GCA_029011515.1	GCF_029011515.1	06-Mar-2023	04-Jun-2025
NZ_CP069279.1	HN002	Korea Research Institute of Bioscience and Biotechnology	GCA_016838705.1	GCF_016838705.1	09-Feb-2021	04-Jun-2025
NZ_AP031420.1	i03-0019-A5	Munehiro Furuichi Keio University school of medicine, Department of Microbiology and Immunology; Shinanomachi 35, Shinjuku-ku, Tokyo 160-8582, Japan	GCA_042848835.1	GCF_042848835.1	13-Oct-2024	04-Jun-2025

NZ_CP010412.1	BF3	Yonsei University	GCA_001281345.1	GCF_001281345.1	14-Sep-2015	04-Jun-2025
NZ_AP018132.1	TMC 3115	Graduate School of Biostudies, Kyoto University	GCA_003573895.1	GCF_003573895.1	03-Oct-2017	04-Jun-2025
NZ_AP024712.1	YIT 10347	Yakult Central Institute	GCA_020892075.1	GCF_020892075.1	03-Nov-2021	04-Jun-2025
NC_014638.1	PRL2010	Laboratory of Probiogenomics, Department of genetics, university of Parma, Italy	GCA_000165905.1	GCF_000165905.1	02-Nov-2010	04-Jun-2025
NC_017999.1	BGN4	Korea Research Institute of Bioscience and Biotechnology (KRIBB)	GCA_000265095.1	GCF_000265095.1	05-Jun-2012	04-Jun-2025
NC_014616.1	S17	Institute of Microbiology and Biotechnology, University of Ulm	GCA_000164965.1	GCF_000164965.1	19-Oct-2010	04-Jun-2025
AP018131.1	JCM 7004	Graduate School of Biostudies, Kyoto University	GCA_003573955.1	-	14-Sep-2017	22-May-2025

Supplementary Table 2. Data collection and refinement statistics.

Construct	WT	E236Q mutant	D107N-E236Q mutant-G2-peptide
Wavelength	0.87	0.97924	0.97924
Resolution range (Å)	68.46 - 1.9 (1.93 - 1.9)	75.61 – 1.8 (1.9 – 1.8)	20.00 – 1.82 1.92 – 1.82
Space group	C 1 2 1	C 1 2 1	P 4_3 2_1 2
Unit cell *a*, *b*, *c* (Å)<br>α, β, γ (°)	161.981 81.037 124.642<br>90 127.83 90	85.42 77.67 80.21<br>90.00 109.50 90.00	59.96 59.96 337.92<br>90.00 90.00 90.00
Total reflections	228381	153825	956026
Unique reflections	85024 (3341)	40665	57044
Multiplicity	2.7 (2.2)	3.8 (3.7)	16.8 (14.7)
Completeness (%)	85.6 (62.9)	88.9 (84.4)	99.9 (100.0)
Mean I/sigma(I)	6.4 (1.4)	7.7 (1.5)	6.4 (0.9)
R-merge	0.113 (0.562)	0.100 (1.018)	0.188 (2.677)
R-meas	0.140 (0.712)	0.116 (1.181)	0.194 (2.773)
R-pim	0.081 (0.429)	0.057 (0.591)	0.045 (0.715)
CC1/2	0.985 (0.667)	0.996 (0.534)	0.997 (0.480)
R_{work} / R_{free}	0.1863/0.2209	0.2008/0.2401	0.2289/0.2751
Number of protein molecules per asymmetric unit	2	1	1
**No. atoms**			
Protein	8274	4056	4057
SO4	-	5	5
G2-peptide	-	-	132
Waters	1114	74	73
***B*-factors (Å^2)**			
Protein	8274	32.316	47.578
SO4	-	58.124	-
G2-peptide	-	-	52.599
Waters	19.54	25.461	37.918
**R.m.s. deviations**			
Bond lengths (Å)	0.013	0.0084	0.0086
Bond angles (Å)	1.35	1.6598	1.795

Statistics for the highest-resolution shell are shown in parentheses.

Supplementary Table 3. A summary of the biochemical characterisations of CAZymes from *Bifidobacterium bifidum* LMG13195

Glycoside hydrolase family		CBMs in the same ORF	Locus tags	GenBank number	Predicted localisation (ref Sig6)	Predicted localisation (DeepLocPro)	Published activity?	Known or putative substrate	Conservation across *B. bifidum* strains assessed
GH1	1		BBJK_01169	BBA47815.1	No signal sequence		No published activity		
GH1	2		BBJK_01541	BBA48062.1	No signal sequence		No published activity		
GH2	3		BBJK_01655	BBA48139.1	No signal sequence	34 % extracellular 64 % cytoplasmic	Lactose not GOS[2]	β-galactose	>95 % identical across strains
GH2	4		BBJK_00662	BBA47484.1	No signal sequence	27 % extracellular 73 % cytoplasmic	Broad activity[2]	β-galactose	>97 % identical across strains
GH2	5	CBM32	BBJK_02214	BBA48495.1	SPI Sec	27 % extracellular 72 % cell wall	Lactose, LacNAc, LNnT[2]	β-galactose	>90 % identical across strains
GH3	6		BBJK_02595	BBA48753.1	No signal sequence	Cytoplasmic	No published activity		
GH13	7		BBJK_00049	BBA47087.1			No published activity	α-linked glucose	
GH13	8		BBJK_00050	BBA47088.1			No published activity	α-linked glucose	
GH13	9		BBJK_00052	BBA47089.1			No published activity	α-linked glucose	
GH13	10		BBJK_01640	BBA48130.1			No published activity	α-linked glucose	
GH13	11		BBJK_01063	BBA47754.1			No published activity	α-linked glucose	
GH13	12		BBJK_00541	BBA47407.1			No published activity	α-linked glucose	
GH13	13		BBJK_03218	BBA49152.1			No published activity	α-linked glucose	
GH13	14	CBM48	BBJK_00828	BBA47596.1			No published activity	α-linked glucose	
GH13	15	CBM48	BBJK_02658	BBA48796.1			No published activity	α-linked glucose	
GH13	16	CBM48	BBJK_02688	BBA48814.1			No published activity	α-linked glucose	
GH16	17	fragment	BBJK_02086	BBA48422.1	No signal sequence		No published activity		
GH20	18		BBJK_00630	BBA47460.1	SPI Sec	43 % extracellular 55 % cell wall	LnbB lacto-N-biosidase, LNT, GA1, Gb5[3]	β-HexNAc	>92 % identical across strains
GH20	19	2x CBM32	BBJK_00776	BBA47560.1	No signal sequence	38 % extracellular 61 % cell wall	BbhI β1,3lacto-N-triose,N-glycan GlcNAcβ1,2Man [4,5]	β-HexNAc	>99 % identical across strains
GH20	20	CBM32	BBJK_03032	BBA49032.1	No signal sequence	37 % extracellular 61 % cell wall	BbhII 6S-GlcNAc'ase[6]	β-HexNAc	>99 % identical across strains
GH20	21		BBJK_01451	BBA48002.1	No signal sequence	29 % extracellular 67 % cytoplasmic	No published activity	β-HexNAc	>98 % identical across strains
GH25	22	3xCBM13	BBJK_01493	BBA48030.1			No published activity	Peptidoglycan	
GH25	23	CBM50	BBJK_02952	BBA48982.1			No published activity	Peptidoglycan	
GH29	24		BBJK_00483	BBA47373.1	SPI Sec	31 % extracellular 69 % cell wall	α1,3/4 fucosidase[7]	α-fucose	>96 % identical across strains
GH32	25		BBJK_02265	BBA48527.1			No published activity		
GH32	26		BBJK_02267	BBA48528.1			No published activity		
GH33	27		BBJK_01327	BBA47922.1	SPI Sec	45 % extracellular 38 % cell wall	SiaBb2. Broad activity[8-10]	α-sialic acid	

GH33	28		BBJK_01326	BBA47921.1	SPI Sec	23 % extracellular 75 % cell wall	SiaBb1. Also has esterase activity[11]	α-sialic acid	
GH36	29		BBJK_01363	BBA47949.1	No signal sequence	Cytoplasmic			>98 % identical across strains
GH42	30		BBJK_00477	BBA47368.1		31 % Extracellular 68 % Cytoplasmic	B216_08266, Galβ1,3/4/6 linkages, 3FL[2]	β-galactose	>99 % identical across strains
GH42	31		BBJK_01679	BBA48156.1		Cytoplasmic	B216_08730, Galβ1,4 linkages[2]	β-galactose	>99 % identical across strains
GH43	32		BBJK_01432	BBA47992.1			No published activity		
GH43	33		BBJK_02021	BBA48381.1			No published activity		
GH43	34		BBJK_02023	BBA48382.1			No published activity		
GH51	35		BBJK_01448	BBA48000.1			No published activity		
GH77	36		BBJK_00833	BBA47600.1			No published activity	α-linked glucose	
GH84	37	3x CBM32	BBJK_00753	BBA47544.1	SPI Sec	23 % extracellular 75 % cell wall	BbhIV, Core 2, β1,3lacto-N-triose, N-glycan GlcNAcβ1,2Man [5]	β-GlcNAc	>98 % identical across strains
GH84	38		BBJK_00930	BBA47665.1	SPI Sec	26 % extracellular 67 % cell wall	BbhV, Core 2, β1,3lacto-N-triose, N-glycan GlcNAcβ1,2Man [5]	β-GlcNAc	>98 % identical across strains
GH89	39	4x CBM32	BBJK_00728	BBA47530.1	No signal sequence	68 % extracellular 31 % cell wall	Stomach group [12]	α-GlcNAc	>98 % identical across strains
GH95	40		BBJK_01721	BBA48185.1	SPI Sec	61 % extracellular 38 % cell wall	α1,2 fucosidase[13]	α-fucose	>97 % identical across strains
GH101	41	CBM32	BBJK_01844	BBA48266.1	No signal sequence	66 % extracellular	No published activity	Mucin cores	>98 % identical across strains
GH110	42	CBM51	BBJK_00385	BBA47302.1	SPI Sec	49 % extracellular 50 % cell wall	Acts on Blood group B[14]	α-galactose	>97 % identical across strains
GH112	43		BBJK_01792	BBA48232.1	No signal sequence	Cytoplasmic	No published activity	Lacto-N-biose	>99 % identical across strains
GH112	44		BBJK_03100	BBA49080.1	No signal sequence	Cytoplasmic	No published activity	Lacto-N-biose	>98 % identical across strains
GH123	45	GH33	BBJK_00867	BBA47625.1	SPI Sec	64 % extracellular 36 % cell wall	No published activity	β-GalNAc α-sialic acid	>98 % identical across strains
GH129	46		BBJK_01138	BBA47796.1	No signal sequence	Cytoplasmic	Acts on Tn antigen[15]	Exo-a-GalNAc'ase	>98 % identical across strains
GH136	47		BBJK_02171	BBA48469.1	SPI Sec	70 % cell wall	No published activity	Human milk oligosaccharides	>92 % identical across strains
GHnc	48		BBJK_02041	BBA48392.1	No signal sequence		No published activity		
PNGaseA	49				SPI Sec		This work	N-glycans	

Supplementary Discussion

A double mutant renders PNGaseA^{Bif} inactive

MALDI-TOF analysis of the G2-peptide following incubation with PNGaseA^{Bif}, PNGaseA^{Bif} E236Q, or PNGaseA^{Bif} D107N–E236Q confirmed that the single mutant retained partial activity whereas the double mutant was catalytically inactive (Supplementary Figure 11). The wild-type enzyme converted the substrate into product ions at approximately m/z 661.7 and 683.7, which are consistent with the deamidated peptide KVADKT detected as protonated and sodiated species, respectively. A signal at approximately m/z 1664.3 was also observed upon turnover and is compatible with a released glycan-associated species. Consistent with partial impairment rather than full inactivation, the E236Q single mutant still generated product peaks, although substantial intact N-glycopeptide remained. By contrast, the D107N–E236Q double mutant was dominated by the intact G2-peptide signal at approximately m/z 2306.6, consistent with loss of detectable turnover under these conditions. Thus, the mass data fit well with a reduced-activity single mutant and an inactive double mutant, making the latter suitable for trapping a substrate-bound state. Notably, the residual activity of E236Q contrasts with the strict requirement inferred for the equivalent glutamate in PNGaseF, indicating that although E236 contributes to catalysis in PNGaseA^{Bif}, its role may not be absolutely equivalent to that of the corresponding residue in PNGaseF, or may not be fully abolished by a glutamine substitution under the conditions tested.

The activity of PNGaseA^{Bif} is not metal-dependent

Notably, a magnesium ion was observed in the active site of the apo structure, and it is coordinated by waters molecules and four residues, both directly and indirectly, in an octahedral geometry (D107, T109, E126, E236; Supplementary Figure 7). The equivalent residues to D107 and E236 in PNGaseF (D60 and E206) have previously been shown to be essential for catalytic activity[1]. To our knowledge, metal has not been implicated in the catalytic activity of PNGases and equivalent metal binding has not been observed in other structural data for PNGase enzymes from other organisms. The structure of PNGaseF (1PNF) was solved with chitobiose in the active site and the superimposition of PNGaseF and PNGaseA^{Bif} shows that this magnesium ion would be in very close proximity to the reducing end of the core GlcNAc, so it likely an artifact of the crystallography conditions (Supplementary Figure 7). To assess the importance of metals to the activity of PNGaseA^{Bif}, activity assays were repeated with the addition of EDTA (Supplementary Figure 7). Activity was still observed, which confirms that metals are not a requirement for catalytic activity.

1. Kuhn, P. et al. Active site and oligosaccharide recognition residues of peptide-N4-(N-acetyl-beta-D-glucosaminyl)asparagine amidase F. *J Biol Chem* **270**, 29493-7 (1995).
2. Ambrogi, V. et al. Characterization of GH2 and GH42 beta-galactosidases derived from bifidobacterial infant isolates. *AMB Express* **9**, 9 (2019).
3. Gotoh, A. et al. Novel substrate specificities of two lacto-N-biosidases towards beta-linked galacto-N-biose-containing oligosaccharides of globo H, Gb5, and GA1. *Carbohydr Res* **408**, 18-24 (2015).
4. Miwa, M. et al. Cooperation of beta-galactosidase and beta-N-acetylhexosaminidase from bifidobacteria in assimilation of human milk oligosaccharides with type 2 structure. *Glycobiology* **20**, 1402-9 (2010).
5. Takada, H., Katoh, T., Sakanaka, M., Odamaki, T. & Katayama, T. GH20 and GH84 beta-N-acetylglucosaminidases with different linkage specificities underpin mucin O-glycan breakdown capability of Bifidobacterium bifidum. *J Biol Chem* **299**, 104781 (2023).
6. Katoh, T. et al. Identification and characterization of a sulfoglycosidase from Bifidobacterium bifidum implicated in mucin glycan utilization. *Biosci Biotechnol Biochem* **81**, 2018-2027 (2017).
7. Sakurama, H. et al. Differences in the substrate specificities and active-site structures of two alpha-L-fucosidases (glycoside hydrolase family 29) from Bacteroides thetaiotaomicron. *Biosci Biotechnol Biochem* **76**, 1022-4 (2012).
8. Sela, D.A. et al. An infant-associated bacterial commensal utilizes breast milk sialyloligosaccharides. *J Biol Chem* **286**, 11909-18 (2011).
9. Kiyohara, M. et al. An exo-alpha-sialidase from bifidobacteria involved in the degradation of sialyloligosaccharides in human milk and intestinal glycoconjugates. *Glycobiology* **21**, 437-47 (2011).
10. Nishiyama, K. et al. Two extracellular sialidases from Bifidobacterium bifidum promote the degradation of sialyl-oligosaccharides and support the growth of Bifidobacterium breve. *Anaerobe* **52**, 22-28 (2018).
11. Yamamoto, K. Biological analysis of the microbial metabolism of hetero-oligosaccharides in application to glycotechnology. *Biosci Biotechnol Biochem* **76**, 1815-27 (2012).
12. Shimada, Y. et al. alpha-N-Acetylglucosaminidase from Bifidobacterium bifidum specifically hydrolyzes alpha-linked N-acetylglucosamine at nonreducing terminus of O-glycan on gastric mucin. *Appl Microbiol Biotechnol* **99**, 3941-8 (2015).
13. Katayama, T. et al. Molecular cloning and characterization of Bifidobacterium bifidum 1,2-alpha-L-fucosidase (AfcA), a novel inverting glycosidase (glycoside hydrolase family 95). *J Bacteriol* **186**, 4885-93 (2004).
14. Wakinaka, T. et al. Bifidobacterial alpha-galactosidase with unique carbohydrate-binding module specifically acts on blood group B antigen. *Glycobiology* **23**, 232-40 (2013).
15. Kiyohara, M. et al. alpha-N-acetylgalactosaminidase from infant-associated bifidobacteria belonging to novel glycoside hydrolase family 129 is implicated in alternative mucin degradation pathway. *J Biol Chem* **287**, 693-700 (2012).
