# Supplementary material for: Structural basis for N-glycopeptide preference in an infant-derived Bifidobacterium bifidum PNGaseA
Source: Nat Commun. 2026 Nov 2;17:11119. doi: 10.1038/s41467-026-78972-1 (PMC13728245; doi:10.1038/s41467-026-78972-1)
Supplement: Supplementary file 3 [file 41467_2026_78972_MOESM5_ESM.pdf]

nature portfolio

Peer Review File

Structural basis for N-glycopeptide preference in an infant-derived Bifidobacterium bifidum PNGaseA

Corresponding Author: Dr Lucy Crouch

This manuscript has been previously reviewed at another journal. This document only contains information relating to versions considered at Nature Communications.

This file contains all reviewer reports in order by version, followed by all author rebuttals in order by version.

Version 0:

Reviewer comments:

Reviewer #1

(Remarks to the Author)
This manuscript reports the biochemical, structural computational characterization of a PNGaseA-family enzyme from Bifidobacterium bifidum LMG13195. The authors show that this enzyme acts on a broad range of N-glycan structures, but preferentially releases N-glycans from glycopeptide-like substrates rather than from denatured or native glycoproteins. The major advance of the study is the determination of the first structures of this PNGaseA-family enzyme, including a glycopeptide-bound complex, which reveals a distinctive β-sheet cradle that contributes to formation of a C-shaped binding pocket appears to support glycopeptide-induced closure around the peptide portion of the substrate. The work is relevant to microbial glycobiology, host-glycan foraging the nutritional ecology of infant-associated bifidobacteria.

Overall, the manuscript is interesting technically strong, principally at the structural perspective. The combination of enzymology, crystallography, molecular dynamics comparative genomic analysis provides a coherent appealing story. In particular, the glycopeptide-bound structure is an important contribution, as previous PNGase structures have not defined in this level of detail how the peptide component of an N-glycopeptide substrate is recognized. The manuscript clearly identifies a mechanistic distinction between PNGaseABif more open PNGase architectures, particularly PNGaseF-like enzymes, the proposed role of the β-sheet cradle in defining an N-glycopeptide-processing niche is compelling.

General comments

Substrate preference
A key conclusion of the manuscript is that PNGaseABif preferentially acts on N-glycans attached to short glycopeptides, rather than on native or denatured glycoproteins. The TLC HPAEC-PAD data support this conclusion, the structural data provide a plausible explanation for it. However, the substrate-preference claim is central to the manuscript would benefit from a more quantitative treatment.

I do not necessarily expect full Michaelis-Menten kinetics across the entire substrate panel, especially given the heterogeneity of glycoprotein-derived substrates the difficulty of normalizing glycan occupancy, peptide length protein context. However, the authors should, where feasible, provide a more quantitative comparison of product release from the existing assays. If such quantification is not feasible because of substrate heterogeneity or limitations of the assay format, the authors should make this clear terms such as “preferentially processes” or “shows higher apparent activity toward” would be more appropriate than implying a fully quantified catalytic preference.

β-sheet cradle
The identification of the β-sheet cradle is the most novel structural aspect of the study. The glycopeptide-bound complex shows that the peptide C-terminal region becomes buried between the catalytic core the cradle, the comparison between apo, mutant substrate-bound states supports a model of glycopeptide-induced cradle closure. This is a strong attractive mechanistic interpretation.

The functional role of the β-sheet cradle is supported primarily by structural comparisons among the apo, mutant

glycopeptide-bound states, together with molecular dynamics simulations. These data provide a convincing framework for the proposed cradle-closure mechanism. If the authors have already generated selected mutants, or can readily do so within the revision period, limited mutational validation of residues involved in peptide recognition or cradle movement would further strengthen the manuscript. However, I do not view this as essential for the main conclusions, since the glycopeptide-bound crystal structure the MD analyses already provide substantial support for the proposed model. A brief clarification of this point in the text would be sufficient if additional mutagenesis is not feasible.

Sequon recognition
The substrate-bound structure provides a valuable view of how PNGaseABif engages the peptide portion of the N-glycopeptide. The observed contacts with the glycosylated Asn especially the +2 Thr residue are convincing within the captured G2-peptide complex. This supports the idea that the enzyme recognizes features of the N-X-T sequon, rather than merely binding the glycan.

However, the current evidence comes from one glycopeptide sequence. Therefore, statements suggesting broad recognition of the canonical N-X-S/T sequon should be made cautiously. The enzyme may indeed recognize general sequon features, but the available structure specifically supports recognition of the N-X-T motif in the tested peptide.

You may consider addressing this point by refining the language, replacing broad statements with more precise wording.

Physiological relevance
The biological model proposed for B. bifidum LMG13195 is interesting helps place PNGaseABif within a broader host-glycan foraging strategy. The predicted extracellular membrane-associated localization of the enzyme is consistent with a role in processing glycopeptides at the cell surface, the broader CAZyme analysis supports the view that this strain has a rich surface-associated capacity for host-glycan degradation.

However, the direct physiological contribution of PNGaseABif to growth or glycan acquisition in B. bifidum is not demonstrated. I do not consider this a problem that needs to be experimentally resolved in the present study, since the main value of the work lies in its biochemical structural insights. Nevertheless, the authors should avoid overstating the in vivo role of the enzyme in the infant gut should clearly distinguish the experimentally supported biochemical function from the proposed ecological model.

Growth assays using N-glycopeptide-rich substrates, milk-derived glycopeptide fractions, or comparisons with PNGaseABif-negative strains would certainly be informative. However, such experiments are beyond the reasonable scope of this primarily biochemical structural study, I would not consider them essential for this nice relevant work.

Reviewer #2

(Remarks to the Author)
Referee comments for: Structural basis for N-glycopeptide preference in an infant-derived Bifidobacterium bifidum PNGaseA

The manuscript by Crouch colleagues investigates the structural basis for N-glycopeptide recognition by a PNGaseA enzyme originating from Bifidobacterium bifidum. Among its most notable strengths, the authors present crystallographic data of PNGaseA in complex with an N-glycopeptide (G2-peptide), which would be the first reported structure of a PNGase family member in complex with a glycopeptide substrate. Crouch et al. provide compelling crystallographic data that offers important insights into the structural determinants governing the release of N-glycans from proteins glycopeptides. The authors also present a thorough comparison between the PNGaseF PNGaseA structures, highlighting a unique beta-barrel cradle-loop mechanism in the latter that appears to be conserved across other PNGaseA enzymes. Additionally, the crystallographic evidence elegantly demonstrates the important role of the Thr +2 residue in substrate recognition by PNGaseA enzymes, as well as the structural basis for the ability of PNGaseA to tolerate fucose within its active site.

While the authors have done a substantial amount of work, certain aspects of the manuscript are less convincing. Specifically, the biochemical experiments exploring substrate scope, the proposed model of host glycan breakdown in B. bifidum LMG 13195. Regarding the glycan utilization model, the authors do not appear to offer any new rationales or mechanistic insight into host glycan utilization by this specific organism; rather, the section largely recapitulates what has already been established in the literature. To meaningfully strengthen this aspect of the work, in vivo experiments using this specific strain would be highly valuable. For example, targeted gene knockouts (including of the PNGaseA in question) to directly assess the impact on N-glycan utilization on growth. RNA transcript analyses of relevant genes would similarly provide experimental support for the glycoside hydrolases involved. With respect to the biochemical experiments, specific concerns are detailed in the comments below should be addressed prior to resubmission.

Overall, while the crystallographic data capturing a PNGase in complex with its glycopeptide substrate is impressive, some of the accompanying data requires further development. We are confident that, with appropriate revisions additional experiments, this work merits publication in Nature Communications; however, the authors should carefully address the comments herein before resubmission.

Comments

1. The authors mention that the unique beta-barrel cradle-loop is largely conserved across the PNGaseA family. It would be

informative if the authors discussed how this structural conservation correlates with the enzymatic activity observed among other PNGaseA members. Specifically, do most PNGaseA enzymes preferentially act on glycopeptides while showing limited activity toward intact proteins? Please address this point within manuscript.
2. The mechanistic discussion would be significantly strengthened by the inclusion of a figure illustrating the proposed reaction mechanisms in the main text to allow readers to follow the discussion more easily. Prior to this, in the introduction perhaps, the authors should summarise the state of knowledge of mechanisms within the PNGase family…..which is confusing as some seem to follow cysteine protease/amidase type mechanisms some the mechanisms proposed here….along with another proposing Thr as nucleophile. In the structure determined here, how far away (in Angstrom) is Asp 107 from the carbonyl carbon of the substrate amide??? Is it close enough for a direct attack??
3. Some of the structural figures are not as useful as we would hope. In particular Figure 5 a 5c. It is hard to see the interactions of the ligand with the protein…. Maybe an additional view or a cartoon would be helpful? The "spindly" structures do not help (but I guess they minimise occlusion of other parts).
4. Figure S5, the glycoprotein incubation experiments using the mammalian tri-antennary complex N-glycan require attention. The gel appearance differs noticeably from the other glycoprotein staining panels. The authors should clarify whether this discrepancy is due to any differences in sample preparation or labelling. Additionally, in the experiments where PNGaseA is incubated with glycopeptide substrates derived from Vibrio coralliilyticus treated glycoprotein, lane 3 (which should contain only sialidase), appears to show N-glycan release somewhat comparably to lane 4 which contains both PNGase sialidase. This is difficult to reconcile since incubation with the sialidase shouldn't result in release of N-glycans this likely reflects contamination. The authors should investigate address this inconsistency. Additionally, for best practice, the authors should really run monosaccharide standards alongside the samples in these TLCs as well…
5. In Figure 4b, the authors may want to visually emphasize the beta-sheet cradle in comparison to PNGaseF. One approach would be to render this structural element in grey, consistent with how it is depicted in Figure 4a/

Minor comments
1) The authors should include a TLC positive control for plant N-glycan release. If PNGaseL is unable to release plant N-glycans, an alternative PNGase previously reported to cleave plant N-glycans should be tested. The authors themselves have described such enzymes in prior work published in PNAS, referencing or employing those enzymes here would strengthen this.
2) The following sentence on page 3 contains a grammatical error: "These different preparations were – no pre-treatment (native), boiling (denatured) proteolytic digestion the highly proteolytic coral pathogen, Vibrio coralliilyticus, to remove most of the protein." Please revise to: "…and proteolytic digestion with the highly proteolytic coral pathogen, Vibrio coralliilyticus, to remove most of the protein."
3) The supplemental figures would benefit from clearer labeling to improve readability without requiring constant reference to the captions. For example, in Figure S3, lane labels such as "Control," "PNGase," "PNGase + Sialidase" would be far more informative than simply numbering them 1, 2, 3, etc. Alternatively, a legend within the figure would achieve the same effect.
4) Please indicate the molecular weight of PNGase any other enzymes used on the relevant gel figures.
5) Figure S6 effectively highlights the unique beta-barrel cradle-loop characteristic of PNGaseA would be a valuable addition to the main text. The authors should consider incorporating it there. Maybe incorporate this into Figure 4?
6) The use of "A" "B" to label the innermost outermost GlcNAc residues in the structure figures may cause confusion, as the same letter convention is also used for the peptide portion. The authors should consider adopting a distinct unambiguous notation…. for example, GlcNAc-1 GlcNAc-2 to avoid overlap with the peptide annotations.
7) The deglycosylation gel experiments could be improved in several respects. Loading less protein would help resolve small shifts resulting from deglycosylation. Additionally, the authors should aim to keep the molecular weight ranges zoom levels consistent across gel panels to facilitate comparison: for instance, Figures S4 S5 appear to be cropped at different regions relative to the positive controls shown within those same figures. The gels in Figure S3 represent a good standard to follow. It would also have been informative to include a fully deglycosylated control to provide a clear reference for complete deglycosylation, as this is currently lacking, Figure S4 would have been a good candidate for this.
8) In the TLC experiments, the authors indicate the presence of sialic acid mannose but do not include monosaccharide standards run alongside the samples. Appropriate monosaccharide controls should be included in all future TLC experiments to support these assignments.

Reviewer #3

(Remarks to the Author)
I co-reviewed this manuscript with one of the reviewers who provided the listed reports. This is part of the Nature Communications initiative to facilitate training in peer review to provide appropriate recognition for Early Career Researchers who co-review manuscripts.

Version 1:

Reviewer comments:

Reviewer #1

(Remarks to the Author)
The manuscript has been strengthened, the major concerns raised in my original review have been satisfactorily addressed.

The new LC-MS/MS analyses time-course experiments provide further additional support for the conclusion that

PNGaseABif preferentially acts on N-glycopeptide substrates. The authors also appropriately clarify the limitations associated with a fully quantitative kinetic analysis have moderated the relevant statements accordingly.

The discussion of the β-sheet cradle is now clearer better justified. The explanation regarding the challenges of mutational validation is reasonable, the structural molecular dynamics data provide sufficient support for the proposed cradle-closure mechanism.

I also appreciate the more careful treatment of sequon recognition, the improved mechanistic discussion, the new explanatory figure, the additional controls biochemical data included throughout the revised manuscript. These revisions improve both the rigor clarity of the work.

Overall, the authors have adequately addressed my comments.

Reviewer #2

(Remarks to the Author)
The authors have responded adequately to our suggestions concerns. The additional structural/mechanistic discussion will be very helpful to others interest in this enzyme class.
The changes made are adequate: we agree that gene knockout etc studies are outside the scope of the current manuscript. Congratulations to the authors...we look forward to seeing it in print.

Open Access This Peer Review File is licensed under a Creative Commons Attribution 4.0 International License, which permits use, sharing, adaptation, distribution and reproduction in any medium or format, as long as you give appropriate credit to the original author(s) and the source, provide a link to the Creative Commons license, and indicate if changes were made.
In cases where reviewers are anonymous, credit should be given to 'Anonymous Referee' and the source.
The images or other third party material in this Peer Review File are included in the article's Creative Commons license, unless indicated otherwise in a credit line to the material. If material is not included in the article's Creative Commons license and your intended use is not permitted by statutory regulation or exceeds the permitted use, you will need to obtain permission directly from the copyright holder.
To view a copy of this license, visit https://creativecommons.org/licenses/by/4.0/

Dear Reviewers,
The Editor suggested that we assess substrate preference more quantitatively and carry out mutational analysis of the β-sheet cradle. To address substrate preference, we now include two additional datasets — a time-course assay (Supplementary Figures 8-10) and LC-MS/MS analysis of the released glycans (Figure 2 and Supplementary Figures 6 and 7) — which further support the broad N-glycan specificity of the enzyme and its preference for glycopeptide substrates. A fully quantitative kinetic treatment is not feasible here, for the reasons set out in our detailed response below. On the cradle, Reviewer 1 noted that mutational analysis was not imperative for the main conclusions, and we agree, because the cradle residues that stabilise the closed state are largely the same residues that read the peptide, so that mutations intended to probe cradle movement would at the same time disrupt peptide recognition and could not cleanly separate the two effects. In our complex, for instance, Asp478 in the cradle and Arg104 in β-sandwich 1 both contact the +2 threonine of the glycopeptide while also forming the cradle–β-sandwich 1 contacts that close the pocket, and only a small number of interface residues such as Asn479 might be altered without directly affecting peptide binding, so that a meaningful mutational dissection of cradle dynamics is far from trivial and would in itself constitute a substantial study. We therefore consider the existing evidence sufficient, since the link between the β-sheet cradle and peptide recognition is well supported and cradle closure upon substrate binding is captured directly by our three crystal structures.

In summary, the main revisions are:

- LC-MS/MS data supporting substrate preference and the broad N-glycan specificity of the enzyme
- a time-course experiment supporting substrate preference
- re-running of all assays to improve consistency between figures
- a new figure illustrating the proposed reaction mechanism
- an improved active-site figure, with the ligand–protein interactions now shown in stereo for clarity

A full point-by-point response to all comments follows below.

Best wishes,
Lucy and Ramón

REVIEWER COMMENTS

Reviewer #1 (Remarks to the Author):

This manuscript reports the biochemical, structural and computational characterization of a PNGaseA-family enzyme from Bifidobacterium bifidum LMG13195. The authors show that this enzyme acts on a broad range of N-glycan structures, but preferentially releases N-glycans from glycopeptide-like substrates rather than from denatured or native glycoproteins. The major advance of the study is the determination of the first structures of this PNGaseA-family enzyme, including a glycopeptide-bound complex, which reveals a distinctive β-sheet cradle that contributes to formation of a C-shaped binding pocket and appears to support glycopeptide-induced closure around the peptide portion of the substrate. The work is relevant to microbial glycobiology, host-glycan foraging and the nutritional ecology of infant-associated bifidobacteria.
Overall, the manuscript is interesting and technically strong, principally at the structural perspective. The combination of enzymology, crystallography, molecular dynamics and comparative genomic analysis provides a coherent and appealing story. In particular, the glycopeptide-bound structure is an important contribution, as previous PNGase structures have not defined in this level of detail how the peptide component of an N-glycopeptide substrate is recognized. The manuscript clearly identifies a mechanistic distinction between PNGaseABif and more open PNGase architectures, particularly PNGaseF-like enzymes, and the proposed role of the β-sheet cradle in defining an N-glycopeptide-processing niche is compelling.
The authors thank the reviewer for taking the time to review our manuscript and provide their thoughtful perspective on several different aspects. Please see below for responses and actions to the specific topics.

General comments

Substrate preference
A key conclusion of the manuscript is that PNGaseABif preferentially acts on N-glycans attached to short glycopeptides, rather than on native or denatured glycoproteins. The TLC and HPAEC-PAD data support this conclusion, and the structural data provide a plausible explanation for it. However, the substrate-preference claim is central to the manuscript and would benefit from a more quantitative treatment.
I do not necessarily expect full Michaelis-Menten kinetics across the entire substrate panel, especially given the heterogeneity of glycoprotein-derived substrates and the difficulty of normalizing glycan occupancy, peptide length and protein context. However, the authors should, where feasible, provide a more quantitative comparison of product release from the existing assays. If such quantification is not feasible because of substrate heterogeneity or limitations of the assay format, the authors should make this clear and terms such as “preferentially processes” or “shows higher apparent activity toward” would be more appropriate than implying a fully quantified catalytic preference.
Response: We agree with the reviewer that full kinetics would be highly desirable for the characterisation of any enzyme. Overall, we find this quite tricky with our current lines of work due to the types of enzymes and substrates that we work with. Our difficulty in this case is primarily 1) determining exactly what our glycan concentration is (start and end), 2) making the concentrations the same at the start between different types of samples, 3) heterogeneity (as you say), 4) expensive substrates, 5) distinguishing between glycosylation sites.
Action: As a compromise, we offer LC-MS/MS data and time course assays of PNGaseL vs PNGaseA against different substrates.

β-sheet cradle
The identification of the β-sheet cradle is the most novel structural aspect of the study. The glycopeptide-bound complex shows that the peptide C-terminal region becomes buried between the catalytic core and the cradle, and the comparison between apo, mutant and substrate-bound states supports a model of glycopeptide-induced cradle closure. This is a strong and attractive mechanistic interpretation.
Response: The authors thank the reviewer for their evaluation of the work.

The functional role of the β-sheet cradle is supported primarily by structural comparisons among the apo, mutant and glycopeptide-bound states, together with molecular dynamics simulations. These data provide a convincing framework for the proposed cradle-closure mechanism. If the authors have already generated selected mutants, or can readily do so within the revision period, limited mutational validation of residues involved in peptide recognition or cradle movement would further strengthen the manuscript. However, I do not view this as essential for the main conclusions, since the glycopeptide-bound crystal structure and the MD analyses already provide substantial support for the proposed model. A brief clarification of this point in the text would be sufficient if additional mutagenesis is not feasible.
Response: We thank the reviewer for this constructive suggestion and for noting that mutational validation is not essential to the main conclusions. The functional role of the β-sheet cradle is established by the direct comparison of the apo, ligand-free E236Q and D107N–E236Q–G2-peptide structures, which capture the open-to-closed transition as crystallographic snapshots, and by the molecular dynamics simulations already presented, which show that the cradle loops are intrinsically mobile in the apo state and retract toward an open conformation upon removal of the G2-peptide. The closed state is further stabilised by specific cradle–β-sandwich 1 contacts (Asp478–Tyr166 and Arg104–Asn479) resolved in the complex. We would also note that a clean mutational dissection of cradle movement is not straightforward, because the residues that stabilise the closed state largely coincide with those that read the peptide: Asp478 and Arg104 each contact the +2 threonine of the glycopeptide while also forming the cradle–β-sandwich 1 contacts that close the pocket, so mutating them would disrupt peptide recognition and cradle closure simultaneously rather than separating the two. Only a few interface residues, such as Asn479, might be altered without directly affecting peptide binding, so meaningful validation would require combinations of mutations across different PNGases and would amount to a substantial study in its own right. We therefore feel that the cradle-closure mechanism is well supported by the available structural and computational data, and, as the reviewer suggests, we have added a brief clarification to the text noting that the model rests on these analyses and that direct mutational validation was not pursued in this study.
Action: we have inserted the following text after the sentence ending “…..the side-chain carbonyl group of Asn479 in the β-sheet cradle."

Together, the three crystallographic states, apo, ligand-free E236Q and the D107N–E236Q–G2-peptide complex, show the cradle in increasingly closed conformations, and the molecular dynamics simulations are consistent with this open-to-closed transition, supporting a glycopeptide-induced cradle-closure mechanism. Although this model rests on structural and computational evidence, direct mutation of the individual cradle contacts would provide independent validation and is a clear objective for future work.

Sequon recognition
The substrate-bound structure provides a valuable view of how PNGaseABif engages the peptide portion of the N-glycopeptide. The observed contacts with the glycosylated Asn and especially the +2 Thr residue are convincing within the captured G2-peptide complex. This supports the idea that the enzyme recognizes features of the N-X-T sequon, rather than merely binding the glycan.
However, the current evidence comes from one glycopeptide sequence. Therefore, statements suggesting broad recognition of the canonical N-X-S/T sequon should be made cautiously. The enzyme may indeed recognize general sequon features, but the available structure specifically supports recognition of the N-X-T motif in the tested peptide.
You may consider addressing this point by refining the language, replacing broad statements with more precise wording.
Response: we agree that the structural evidence comes from a single NXT glycopeptide (KVANKT) and have changed the manuscript to reflect this. Broad references to the NXS/T sequon have been replaced with the specific NXT motif present in the tested substrate, and the statements about general sequon recognition are now framed as an inference that still needs to be tested for Ser-containing sequons.
Action: we have made the following changes throughout the manuscript (changes are highlighted in blue):

- Abstract (line ~38): "…concentrated around the +2 Thr residue of the NXT sequon of the bound glycopeptide,**…"**
- Results header (line ~257): "Recognition of the peptide is predominantly driven by the asparagine and the +2 threonine"
- Line ~259: "Recognition of the peptide is concentrated around the glycosylated Asn and, especially, the +2 Thr of the NXT sequon present in the N-glycopeptide".
- Line ~274–276: "…selectively reads the Asn and +2 Thr residues of the NXT sequon in the captured complex**."**
- Discussion (line ~501–503): "…recognition is concentrated around the glycosylated Asn and the +2 Thr of the bound glycopeptide. Since the bound glycopeptide carries a Thr at the +2 position, our data define recognition of an NXT motif, leaving open whether Ser-containing NXS sequons are recognised in the same way."
- Discussion (line ~505–509): "…instead reads key elements of the N-glycosylation sequon itself. This suggests that the same NXS/T logic that guides N-glycan installation by oligosaccharyltransferases and soluble bacterial N-glycosyltransferases may also be exploited here during N-glycan removal, although this is based on a single NXT glycopeptide and would need additional substrates to confirm for NXS sequons."

Physiological relevance
The biological model proposed for B. bifidum LMG13195 is interesting and helps place PNGaseABif within a broader host-glycan foraging strategy. The predicted extracellular and membrane-associated localization of the enzyme is consistent with a role in processing glycopeptides at the cell surface, and the broader CAZyme analysis supports the view that this strain has a rich surface-associated capacity for host-glycan degradation.
However, the direct physiological contribution of PNGaseABif to growth or glycan acquisition in B. bifidum is not demonstrated. I do not consider this a problem that needs to be experimentally resolved in the present study, since the main value of the work lies in its biochemical and structural insights. Nevertheless, the authors should avoid overstating the in vivo role of the enzyme in the infant gut and should clearly distinguish the experimentally supported biochemical function from the proposed ecological model.
Response: The authors thank the reviewer in pointing out that we should tread carefully in our wording.
Action: When reviewing the text, we kept this in mind.

Growth assays using N-glycopeptide-rich substrates, milk-derived glycopeptide fractions, or

comparisons with PNGaseABif-negative strains would certainly be informative. However, such experiments are beyond the reasonable scope of this primarily biochemical and structural study, and I would not consider them essential for this nice and relevant work.
Response: The reviewer makes a very valid point. We usually do combine *in vivo* and *in vitro* experiments in our publications. In this case, we would prefer not to include this data in this particular publication.
Action: No action taken.

Reviewer #2 (Remarks to the Author):

Referee comments for: Structural basis for N-glycopeptide preference in an infant-derived Bifidobacterium bifidum PNGaseA

The manuscript by Crouch and colleagues investigates the structural basis for N-glycopeptide recognition by a PNGaseA enzyme originating from Bifidobacterium bifidum. Among its most notable strengths, the authors present crystallographic data of PNGaseA in complex with an N-glycopeptide (G2-peptide), which would be the first reported structure of a PNGase family member in complex with a glycopeptide substrate. Crouch et al. provide compelling crystallographic data that offers important insights into the structural determinants governing the release of N-glycans from proteins and glycopeptides. The authors also present a thorough comparison between the PNGaseF and PNGaseA structures, highlighting a unique beta-barrel cradle-loop mechanism in the latter that appears to be conserved across other PNGaseA enzymes. Additionally, the crystallographic evidence elegantly demonstrates the important role of the Thr +2 residue in substrate recognition by PNGaseA enzymes, as well as the structural basis for the ability of PNGaseA to tolerate fucose within its active site.
The authors thank the reviewer for taking the time to review our manuscript and provide their thoughtful perspective on several different aspects. Please see below for responses and actions to the specific topics.

While the authors have done a substantial amount of work, certain aspects of the manuscript are less convincing. Specifically, the biochemical experiments exploring substrate scope, and the proposed model of host glycan breakdown in B. bifidum LMG 13195. Regarding the glycan utilization model, the authors do not appear to offer any new rationales or mechanistic insight into host glycan utilization by this specific organism; rather, the section largely recapitulates what has already been established in the literature.
Response: The reviewer's assessment is correct; the aim of the model was to put everything together in one place to summarise *B. bifidum* glycobiology for the community. We believe that we have highlighted areas that are also unexplored. To our knowledge, this has not been done before.
Action: Updated the figure with further information of recent publications.

To meaningfully strengthen this aspect of the work, in vivo experiments using this specific strain would be highly valuable. For example, targeted gene knockouts (including of the PNGaseA in question) to directly assess the impact on N-glycan utilization and on growth. RNA transcript analyses of relevant genes would similarly provide experimental support for the glycoside hydrolases involved. With respect to the biochemical experiments, specific concerns are detailed in the comments below and should be addressed prior to resubmission.
Response: We completely agree with the reviewer on the value of having *in vivo* work to accompany enzymology and mechanistic insights (see comment above). It is also not possible to complete gene knockouts within the timeframe of this manuscript.

Overall, while the crystallographic data capturing a PNGase in complex with its glycopeptide substrate is impressive, some of the accompanying data requires further development. We are confident that, with appropriate revisions and additional experiments, this work merits publication in Nature Communications; however, the authors should carefully address the comments herein before resubmission.
The authors thank the reviewer for their detailed reflection of our work and endorsement.

Comments

1. The authors mention that the unique beta-barrel cradle-loop is largely conserved across the

PNGaseA family. It would be informative if the authors discussed how this structural conservation correlates with the enzymatic activity observed among other PNGaseA members. Specifically, do most PNGaseA enzymes preferentially act on glycopeptides while showing limited activity toward intact proteins? Please address this point within manuscript.

Response: Great question! In the description of the Results section "PNGaseABif preferentially cleaves N-glycans from glycopeptides" the starting sentence is "Previously characterised members of the PNGaseA superfamily exhibit a preference for N-glycans that are covalently linked to short peptide rather than native proteins". This refers predominantly to the commercially-available PNGaseA purified from almond, which has been characterised to have this activity. As far as we are aware, no one else has compared between substrates with changes to the substrates protein component for PNGaseA family members. There are a couple of examples of comparing the activities of different PNGaseA enzymes, so partial investigations into specificities. I think trying to compare our results to that would be inconclusive.

2. The mechanistic discussion would be significantly strengthened by the inclusion of a figure illustrating the proposed reaction mechanisms in the main text to allow readers to follow the discussion more easily. Prior to this, in the introduction perhaps, the authors should summarise the state of knowledge of mechanisms within the PNGase family…..which is confusing as some seem to follow cysteine protease/amidase type mechanisms and some the mechanisms proposed here….along with another proposing Thr as nucleophile. In the structure determined here, how far away (in Angstrom) is Asp 107 from the carbonyl carbon of the substrate amide??? Is it close enough for a direct attack??

Response: We thank the reviewer for these suggestions, which strengthen the mechanistic part of the manuscript. We have added a new figure to the main text showing the two possible reaction mechanisms, so that readers can follow the discussion more easily. We have also added a short summary in the Introduction of what is currently known about catalysis across peptide enzymes and related glycoamidases. This now makes clear that cytoplasmic PNGases (Png1/NGLY1) use a cysteine protease-like, transglutaminase-type mechanism with a Cys-His-Asp triad, whereas PNGaseF and the PNGaseA enzyme studied here rely on acidic residues. We also clarify that related N-terminal nucleophile hydrolases, including glycosylasparaginases, use a processed N-terminal residue, often Thr, as the catalytic nucleophile, and that this mechanism has previously been distinguished from that of PNGaseF. Regarding the distance requested by the reviewer, in the structure determined here the OD1 atom of Asn107 in the D107N–E236Q complex, which approximates the position of one Asp107 carboxylate oxygen after reversion to the wild-type residue, is 3.2 Å from the carbonyl carbon of the scissile amide. This distance is compatible with a direct nucleophilic attack. Because the crystal structure carries the D107N substitution, we also relied on molecular dynamics simulations after reverting the mutations, in which the Asp107 carboxylate-to-carbonyl carbon distance samples values around 3–4 Å, within the range expected for a direct nucleophilic attack. Nevertheless, because distance alone does not define the complete catalytic geometry and no catalytic water is observed in the crystal structure, we present both the water-mediated and direct Asp107-assisted mechanisms as plausible scenarios rather than assigning a definitive catalytic pathway.

Action: we have made the following changes:

1.-) New figure (two catalytic mechanisms). We added a new figure to the main text (Figure 5) showing the two proposed catalytic mechanisms, cited at line 371 ("…the current structural and computational data support two plausible catalytic scenarios…").

2.-) Introduction paragraph. We added the following paragraph in line 60 ("Among PNGases, structural....."):

Among peptide:N-glycosidases (PNGases) and related glycoamidases, catalysis is not uniform, and several different mechanisms have been described or proposed. The cytoplasmic PNGases Png1/NGLY1, which act on misfolded glycoproteins during ER-associated degradation, belong to the transglutaminase-like superfamily and use a cysteine protease-type mechanism, with a Cys-His-Asp catalytic triad in which cysteine acts as the nucleophile [PMID: 15964983]. By contrast, secreted PNGaseF uses an acidic-residue-based catalytic centre. Structural understanding of this class has been dominated by PNGaseF, whose crystal structures revealed a catalytic core formed by two β-sandwich domains and, in a complex with chitobiose, identified Asp60, Glu118 and Glu206 as key residues for catalysis and glycan recognition (PMID: 7881905; PMID: 7493989). For comparison, the related glycosylasparaginases use a mechanistically distinct route in which a processed N-terminal threonine acts as both the base and the nucleophile, a hallmark of the N-terminal nucleophile (Ntn) hydrolase family (PMID: 8953276). This mechanism was previously argued to differ from that of

PNGaseF (PMID: 7493989). However, because these PNGaseF studies did not capture a full N-glycopeptide substrate, it remained unclear how secreted PNGases recognize.....
3.-) Asp107–carbonyl geometry and MD analysis. We added the following after ~ line 353 ("…corresponding in the wild-type enzyme to Asp107 and Glu236."):
The substrate-bound complex also provides insight into the catalytic mechanism. The scissile Asn is positioned between residues 107 and 236, corresponding in the wild-type enzyme to Asp107 and Glu236. In the D107N–E236Q–G2-peptide complex, the OD1 atom of Asn107, which approximates the position of one Asp107 carboxylate oxygen after reversion to the wild-type residue, is 3.2 Å from the carbonyl carbon of the scissile amide. This distance places residue 107 within a range compatible with direct nucleophilic attack. Because the Asn and Gln substitutions are expected to preserve the overall hydrogen-bonding geometry of the native acidic residues,....

We also expanded the corresponding MD analysis to assess whether Asp107 samples a geometry compatible with direct nucleophilic attack:
To assess the structural stability of the substrate-bound complex and probe the participation of Asp107 in catalysis, MD simulations of 500 ns were performed on the PNGaseABif–G2-peptide complex after reverting the D107N and E236Q mutations to the wild-type residues. Backbone RMSD of the protein remained consistently stable across all replicas (Supplementary Figure 16a). The distance between the Asp107 carboxylate oxygen and the carbonyl carbon of the scissile amide was monitored to test the catalytic competence of a direct nucleophilic attack (Supplementary Figure 16b,e). Although this distance tends to increase during the early phases of the simulations, it transiently visits values of ~3–4 Å, within the expected range for this attack to occur (~3 Å). Consistent with this interpretation, the E236Q substitution does not abolish activity, supporting a primary role for Glu236 in substrate polarization rather than direct catalytic turnover.

3. Some of the structural figures are not as useful as we would hope. In particular Figure 5 a and 5c. It is hard to see the interactions of the ligand with the protein…. Maybe an additional view or a cartoon would be helpful? The "spindly" structures do not help (but I guess they minimise occlusion of other parts).
Response: We agree that the original Figure 5a and 5c made it hard to see the interactions between the ligand and the protein. We have remade both panels in stereo, which gives a clearer view of these contacts while keeping the occlusion of other parts to a minimum. We hope the reviewer finds the revised panels easier to follow.
Action: We have remade these (now Figure 4a and 4c) in stereo to make the ligand–protein interactions clearer.

4. Figure S5, the glycoprotein incubation experiments using the mammalian tri-antennary complex N-glycan require attention. The gel appearance differs noticeably from the other glycoprotein staining panels. The authors should clarify whether this discrepancy is due to any differences in sample preparation or labelling.
Response: Yes, this is true. In powerpoint of the original figures the image looks fine. The PDF process must have had a weird effect here that we missed. Many apologies.
Action: Regardless, these experiments were re-run (see below).

Additionally, in the experiments where PNGaseA is incubated with glycopeptide substrates derived from Vibrio coralliilyticus treated glycoprotein, lane 3 (which should contain only sialidase), appears to show N-glycan release somewhat comparably to lane 4 which contains both PNGase and sialidase. This is difficult to reconcile since incubation with the sialidase shouldn't result in release of N-glycans and this likely reflects contamination. The authors should investigate and address this inconsistency. Additionally, for best practice, the authors should really run monosaccharide standards alongside the samples in these TLCs as well…
Response: Yes, we agree with the reviewer.
Action: We have repeated the assays and analysis to provide greater conformity across the different figures. Relevant monosaccharides included.

5. In Figure 4b, the authors may want to visually emphasize the beta-sheet cradle in comparison to PNGaseF. One approach would be to render this structural element in grey, consistent with how it is depicted in Figure 4a/
Response: We thought that the figure was already adequate regarding displaying the uniqueness of

the cradle.
Action: However, we have completed what the reviewer has asked.

Minor comments
1) The authors should include a TLC positive control for plant N-glycan release. If PNGaseL is unable to release plant N-glycans, an alternative PNGase previously reported to cleave plant N-glycans should be tested. The authors themselves have described such enzymes in prior work published in PNAS, and referencing or employing those enzymes here would strengthen this.
Response: Thank you for highlighting this, PNGaseL is able to remove plant and insect N-glycans.
Action: We repeated assays on the plant and insect substrates available. We cannot generate the peptide version of this substrate.

2) The following sentence on page 3 contains a grammatical error: "These different preparations were – no pre-treatment (native), boiling (denatured) and proteolytic digestion the highly proteolytic coral pathogen, Vibrio coralliilyticus, to remove most of the protein." Please revise to: "…and proteolytic digestion with the highly proteolytic coral pathogen, Vibrio coralliilyticus, to remove most of the protein."
Action: Changed

3) The supplemental figures would benefit from clearer labeling to improve readability without requiring constant reference to the captions. For example, in Figure S3, lane labels such as "Control," "PNGase," and "PNGase + Sialidase" would be far more informative than simply numbering them 1, 2, 3, etc. Alternatively, a legend within the figure would achieve the same effect.
Action: We have changed the labelling

4) Please indicate the molecular weight of PNGase and any other enzymes used on the relevant gel figures.
Action: A PNGase-only control was run on all gels for clarity and the molecular weights are stated in the legends.

5) Figure S6 effectively highlights the unique beta-barrel cradle-loop characteristic of PNGaseA and would be a valuable addition to the main text. The authors should consider incorporating it there. Maybe incorporate this into Figure 4?
Response: We are not sure which supplementary figure or image the reviewer is referring to? If the reviewer still holds their view, then please elaborate further.

6) The use of "A" and "B" to label the innermost and outermost GlcNAc residues in the structure figures may cause confusion, as the same letter convention is also used for the peptide portion. The authors should consider adopting a distinct and unambiguous notation…. for example, GlcNAc-1 and GlcNAc-2 to avoid overlap with the peptide annotations.
Response: We agree that the previous labelling could potentially cause confusion. Rather than changing the glycan nomenclature, we have retained the A, B, etc. identifiers but now display them as superscripts to the corresponding monosaccharide names (e.g. $GlcNAc^{A}$ and $GlcNAc^{B}$). This clearly distinguishes the glycan residue labels from those used for the peptide.
Action: We have revised the structural figures so that the A, B, etc. glycan identifiers are shown as superscripts to the corresponding sugar names, avoiding possible confusion with the peptide labels while retaining the existing glycan nomenclature.

7) The deglycosylation gel experiments could be improved in several respects. Loading less protein would help resolve small shifts resulting from deglycosylation. Additionally, the authors should aim to keep the molecular weight ranges and zoom levels consistent across gel panels to facilitate comparison: for instance, Figures S4 and S5 appear to be cropped at different regions relative to the positive controls shown within those same figures.
The gels in Figure S3 represent a good standard to follow. It would also have been informative to include a fully deglycosylated control to provide a clear reference for complete deglycosylation, as this is currently lacking, Figure S4 would have been a good candidate for this.
Response: We originally cropped the gels to focus in on the relevant area for the reader.
Action: We have used the protein ladder to crop the gels consistently across substrates rather than honing in on the relevant areas. The fully de-glycosylated control is produced using our positive control PNGaseL, which is included throughout.

8) In the TLC experiments, the authors indicate the presence of sialic acid and mannose but do not include monosaccharide standards run alongside the samples. Appropriate monosaccharide controls should be included in all future TLC experiments to support these assignments.
Action: For all new TLCs performed for these corrections, we have included appropriate controls.

Reviewer #3 (Remarks to the Author):

I co-reviewed this manuscript with one of the reviewers who provided the listed reports. This is part of the Nature Communications initiative to facilitate training in peer review and to provide appropriate recognition for Early Career Researchers who co-review manuscripts.
Thank you for spending time reading our manuscript – we hope you have found the process useful.

Dear Reviewers,
Thank you very much for taking another look at our manuscript and the positive endorsement. We appreciate the time and effort.
Best wishes
Lucy and Ramón

Reviewer #1 (Remarks to the Author):
The manuscript has been strengthened, and the major concerns raised in my original review have been satisfactorily addressed.
The new LC-MS/MS analyses and time-course experiments provide further additional support for the conclusion that PNGaseABif preferentially acts on N-glycopeptide substrates. The authors also appropriately clarify the limitations associated with a fully quantitative kinetic analysis and have moderated the relevant statements accordingly.
The discussion of the β-sheet cradle is now clearer and better justified. The explanation regarding the challenges of mutational validation is reasonable, and the structural and molecular dynamics data provide sufficient support for the proposed cradle-closure mechanism.
I also appreciate the more careful treatment of sequon recognition, the improved mechanistic discussion, the new explanatory figure, and the additional controls and biochemical data included throughout the revised manuscript. These revisions improve both the rigor and clarity of the work.
Overall, the authors have adequately addressed my comments.

Reviewer #2 (Remarks to the Author):
The authors have responded adequately to our suggestions and concerns. The additional structural/mechanistic discussion will be very helpful to others interest in this enzyme class.
The changes made are adequate: we agree that gene knockout etc studies are outside the scope of the current manuscript.
Congratulations to the authors…we look forward to seeing it in print.
